# Inverse-IMPRESSION: A Graph-Based Platform for Molecular Structure Elucidation from Experimental NMR Spectroscopic Properties

**DOI:** 10.1021/jacs.6c14119

**Published:** 2026-10-31

**Authors:** Zheqi Jin, Grace Armitage, Richard Cox, Ben Honoré, Mohammad Golbabaee, Craig Butts

**Affiliations:** † School of Chemistry, 1980 University of Bristol, Cantock’s Close, Bristol BS8 1TS, United Kingdom; ‡ School of Engineering Mathematics and Technology, University of Bristol, Ada Lovelace Building, Tankard’s Cl, Bristol BS8 1TW, United Kingdom

## Abstract

Here, we present a platform built on our inverted graph transformer network, IMPRESSION-G2, which can accurately and rapidly reconstruct molecular bonding directly from experimental nuclear magnetic resonance (NMR) spectroscopic information. It comprises three interconnected stages: a one-shot model that reads in NMR parameters and uses these to predict bond connectivity between atoms; a structure correction stage that corrects the predicted structures by removing uncertain bonds and iteratively reassigning them; and noise-augmented multishot prediction, generating an ensemble of candidate structures, which are ranked to identify the best-fit structure. By integrating a range of ^1^H and ^13^C NMR data, including two-dimensional (2D) experiments such as COSY, HSQC, and HMBC, the inverse-IMPRESSION platform correctly identifies the structures of 77.8% of molecules with up to 30 heavy atoms (H, C, N, O, and F) using simulated NMR data, and 11 of 19 (58%) molecules using experimental NMR data. The experimental structures solved are representative of the complex structures in synthetic and natural products that routinely challenge chemists, assessed by high nSPS scores (greater than 40) for successfully elucidated structures and molecular weights of up to 480 Da. The inverse-IMPRESSION framework thus provides a highly effective approach for automated molecular structure elucidation by using graph-based machine learning on experimental data.

## Graphical abstract

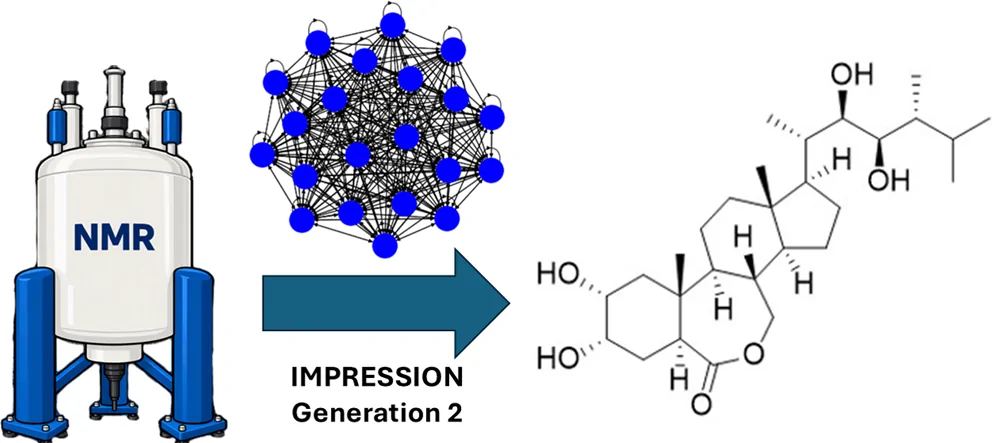

## Introduction

Accurate and rapid molecular structure elucidation is a fundamental task across fields, such as organic synthesis, materials science, and drug discovery. Among available analytical techniques, nuclear magnetic resonance (NMR) spectroscopy plays a central role because of its ability to provide detailed information on local chemical environments, including atomic connectivity, functional groups, and stereochemistry. However, interpreting NMR spectra remains a challenging and time-consuming process, with the primary bottleneck arising from the combinatorial explosion of possible molecular structures. As the molecular size increases, the number of feasible candidate structures grows exponentially, rendering exhaustive enumeration and manual selection impractical on the order of 10^60^ molecules.^1^ Therefore, computer-assisted approaches are essential to systematically manage this combinatorial complexity and enable practical and scalable molecular structure elucidation.

Traditional computer-assisted structure elucidation (CASE) systems^2–5^ that determine molecular structure from spectroscopic data, especially NMR data, typically rely on logical-combinatorial algorithms to derive molecular fragments from experimental spectra and assemble them into candidate structures, which are subsequently ranked based on their agreement with the input data. Modern CASE systems have incorporated density functional theory (DFT),^6^ statistical tools,^7–9^ and genetic algorithms,^10^ to improve the accuracy and efficiency of structure generation and ranking. However, the reliance on exhaustive structure generation and filtering often results in the ranking of highly similar structures at a high computational cost, limiting full automation for complex molecules.

Recent advances in artificial intelligence (AI) and machine learning have driven the emergence of data-driven approaches to molecular structure elucidation. These approaches are at an early stage and require large training data sets to effectively capture structure-spectrum relationships; however, they have the potential to enable fully automated and substantially faster predictions compared to traditional rule-based methods. Current data-driven methods of this type can be broadly categorized into large language model (LLM)-based methods^11–20^ and graph-based methods.^21–24^

Graph-based methods represent molecules explicitly as graphs, in which atoms correspond to nodes and bonds, or atom pairs correspond to edges. This representation enables atom- and pair-specific information, such as chemical shifts and coupling constants, to be naturally incorporated as node and edge features, providing a flexible and chemically intuitive framework for representing both the molecular structure and spectroscopic data. Graph neural networks (GNNs) iteratively aggregate information from neighboring nodes and edges to update the molecular graph, enabling property prediction in structure elucidation tasks, including bond orders and interatomic distances. Several representative efforts highlight both the promise and the limitations of current graph-based approaches. Sridharan et al.^23^ proposed a stepwise strategy combining Monte Carlo tree search (MCTS) with GNNs but largely restricted to small molecules containing fewer than ten heavy atoms. More recently, Yang et al.^24^ integrated diffusion models with GNNs to iteratively refine molecular bonding patterns, conditioning the generation process on the embedded NMR spectra. This approach achieved a top-1 accuracy of 62% for simulated NMR data, but the performance decreased substantially to 11% for experimental data comprising molecules with up to 25 heavy atoms. We recently reported a proof-of-concept graph-based approach^25^ using an inverted implementation of the IMPRESSION-G2 machine learning architecture, which demonstrated that graph-based methods can achieve near 100% success if idealized simulated NMR data are available, but that drops to 33% with ablated data. The IMPRESSION-G2 model^26^ was originally developed to predict chemical shifts and scalar coupling constants from 3D molecular structures represented by interatomic distances. In its inverted form,^25^ IMPRESSION-G2 predicted interatomic distances (and hence molecular structure) directly from the NMR data. This provides important evidence that molecular structure elucidation can be formulated as an edge prediction task on molecular graphs and demonstrates that, in the hypothetical limit of complete NMR information, structures can be reconstructed reliably.

On the other hand, LLM-based approaches typically depend on Transformer encoder-decoder architectures and represent molecules using SMILES (Simplified Molecular-Input Line-Entry System) style strings, with NMR data encoded as text sequences. This compact representation facilitates efficient storage and enables training on large collections of molecular and spectroscopic data, which can overcome the lack of a more task-relevant molecular graph representation. While it is challenging to directly compare the performance of models due to variability in methods, representations, and training and test data sets reported in the literature, the reported performances of these LLM models are broadly comparable to those achieved by GNN-based methods. For example, Hu et al.^17^ reported accuracies of 55% on simulated ^1^H and ^13^C NMR spectra and 21% on experimental data using a pretraining and fine-tuning strategy. Similarly, the NMRMind system^13^ achieved 92% accuracy on “comprehensive” simulated 1D and 2D NMR data and 71% on ablated NMR data. On a set of 12 experimental molecules, it achieved Top-1 accuracy of 33%, with the largest solved molecule having a mass of 399.5 Da. The recently reported NMR-Solver^14^ achieved a Top-1 accuracy of 60% on experimentally synthesized molecules; however, it incorporated prior knowledge of reactant structures as additional constraints, which thus explores a substantially reduced chemical space relative to methods that rely solely on spectroscopic data. In addition to these peer-reviewed studies, several recent preprints and workshop papers, including NMIRacle,^18^ NMRPeak^19^ and NMRAgent,^20^ have also reported promising performance in NMR-based molecular structure elucidation.

Herein, we present an alternative framework for our inverse-IMPRESSION platform in which edge prediction can be expressed as prediction of bond probabilities, that is, the likelihood that two atoms are bonded given a set of NMR data. This simplified expression more directly maps onto the skeletal molecular structure prediction task, where the primary objective is to infer atomic connectivity without explicitly resolving 3D features such as stereochemistry or conformation. This inverse-IMPRESSION platform comprises interconnected models based on the IMPRESSION-G2 architecture, each employing distinct node and edge features and being responsible for a specific component of the structure elucidation process. A noise augmentation multishot strategy is applied to allow the model to generate multiple candidate structures for each prediction event, which are subsequently ranked to identify the best-fit structure. The noise levels are selected to reflect the experimental variability in the NMR measurements (arising from differences in solvent, temperature, concentration, etc.) as well as the uncertainty coming from the DFT calculations used to generate the training data. Together, this framework operates as a unified platform to produce 2D molecular structures with assigned bond orders and demonstrates reliability and robustness across both simulated and experimental NMR data sets. Notably, it successfully predicts complex organic molecules, including experimental examples that represent challenging real-world tasks for experienced structure elucidation experts (exemplified by high molecular mass and nSPS structures, *vide infra*).

## Methods

### Data Sets

A data set comprising 122,000 pharmaceutically relevant molecules containing H, C, N, O, and F atoms, each with no more than 30 non-hydrogen atoms, was derived from the public PubChem^27^ (72 998 molecules) and ChEMBL^28^ (49,002 molecules) databases. To assess potential molecular overlap and data leakage between training and testing data sets, all stereochemical information was removed from the molecular structures, which were subsequently converted to canonical SMILES strings by RDKit.^29^ The resulting canonical SMILES strings were compared to ensure that no molecules share the same 2D connectivity.

For each molecule, 100 conformers were generated and optimized using the Merck Molecular Force Field^30^ (MMFF) as implemented in RDKit,^29^ and their corresponding MMFF energies were computed. NMR parameters, including chemical shifts and scalar coupling constants, were subsequently predicted for each conformer using the IMPRESSION-G2 model,^26^ which provides DFT-level accuracy at a substantially lower computational cost. The final molecular NMR parameters were obtained as Boltzmann-weighted averages over all conformers using their MMFF energies (Supporting Figure 3).

Molecular structures were encoded as fully connected graphs (Supporting Figure 2), with atomic numbers assigned as node features and atom pair labels, e.g., H–C or C–O, assigned as edge features.

The experimental NMR parameters used by the model were the corresponding equivalent information to NMR Data Set B (^1^H and ^13^C chemical shifts, COSY, HSQC, HMBC correlations). Data for compounds 2, 4–8, and 12–17 were taken directly from ref 32, while experimental data for compounds 1, 3, and 9–11 were measured in this work, processed using standard automated spectrum processing (Fourier transform, baseline correction, phasing), and peak-picked to extract the relevant spectroscopic properties before model input. For all compounds, the extracted chemical shifts were incorporated as node features, while scalar coupling correlations were assigned as edge features.

Coupling correlations were assigned a value of 0 when the corresponding scalar coupling constants were below 2 Hz, as such couplings would give rise to very weak or unobservable NMR correlation peaks under the typical experimental conditions. The remaining coupling constants were converted into categorical labels representing correlations (peaks) arising from different 2D NMR experiments: 1 for ^1^H–^1^H COSY, 2 for ^1^H–^13^C HSQC, and 3 for ^1^H–^13^C HMBC.

This data set was split into a training set of 120,000 molecules (4,974,382 nodes and 107,317,458 edges) and a test set of 2000 molecules (86,645 nodes and 1,923,615 edges). To distinguish these data sets from the fragment training subset, they are termed the full-molecule training set and full-molecule test set, respectively. From the full-molecule training set, 60,000 molecules containing at least two oxygen atoms were randomly sampled and used to construct a fragment training subset for the stepwise model. For each selected molecule, two molecular fragments were generated using a breadth-first search (BFS) algorithm, yielding a total of 120,000 molecular fragments for training (see Supporting Section 1.3.2 for details).

### NMR Data Sets

Three sets of NMR parameters were constructed to train the one-shot and stepwise models in the inverse-IMPRESSION platform, each mimicking a different experimental NMR scenario. NMR data sets A and B are discussed in the main text, whereas the results obtained using NMR data set C are primarily presented in the Supporting Information.

NMR Data Set A represents an idealized control scenario where NMR chemical shifts and scalar coupling constants (rather than the coupling correlations used in NMR Data Set B) are available for all nuclei, including those that are not experimentally observable or routinely measurable. Although unrealistic, this setting provides an estimate of the upper bound of the reconstruction accuracy.

NMR data set B represents an experimentally relevant scenario in which only the most commonly used NMR experiments are available. It includes chemical shifts for ^1^H and ^13^C nuclei and scalar coupling correlations for H–H and C–H atom pairs, corresponding to ^1^H–^1^H COSY, ^1^H–^13^C HSQC, and ^1^H–^13^C HMBC spectra.

NMR data set C represents a more comprehensive experimental scenario in which all experimentally accessible NMR experiments are available. It includes chemical shifts for ^1^H, ^13^C, ^15^N, and ^19^F nuclei and scalar coupling correlations for H–H, C–H, N–H, and F–C atom pairs, corresponding to ^1^H–^1^H COSY, ^1^H–^13^C HSQC, ^1^H–^13^C HMBC, ^1^H–^15^N HSQC, ^1^H–^15^N HMBC, ^19^F–^13^C HSQC, and ^19^F–^13^C HMBC spectra.

Unless otherwise specified, COSY, HSQC, and HMBC hereafter refer to ^1^H–^1^H COSY, ^1^H–^13^C HSQC, and ^1^H–^13^C HMBC, respectively.

### Models in the Inverse-IMPRESSION Platform

The inverse-IMPRESSION platform (Figure 1a) comprises four Graph Transformer Network (GTN) models, which share the same neural network architecture (Supporting Section 1.2.1 and 1.2.3) as the previously reported IMPRESSION-G2 model.^26^ Each model was trained separately by using task-specific node and edge features (details below). Full details on training methodology can be found in Supporting Section 1.2.4–1.2.6.

**Figure 1:**
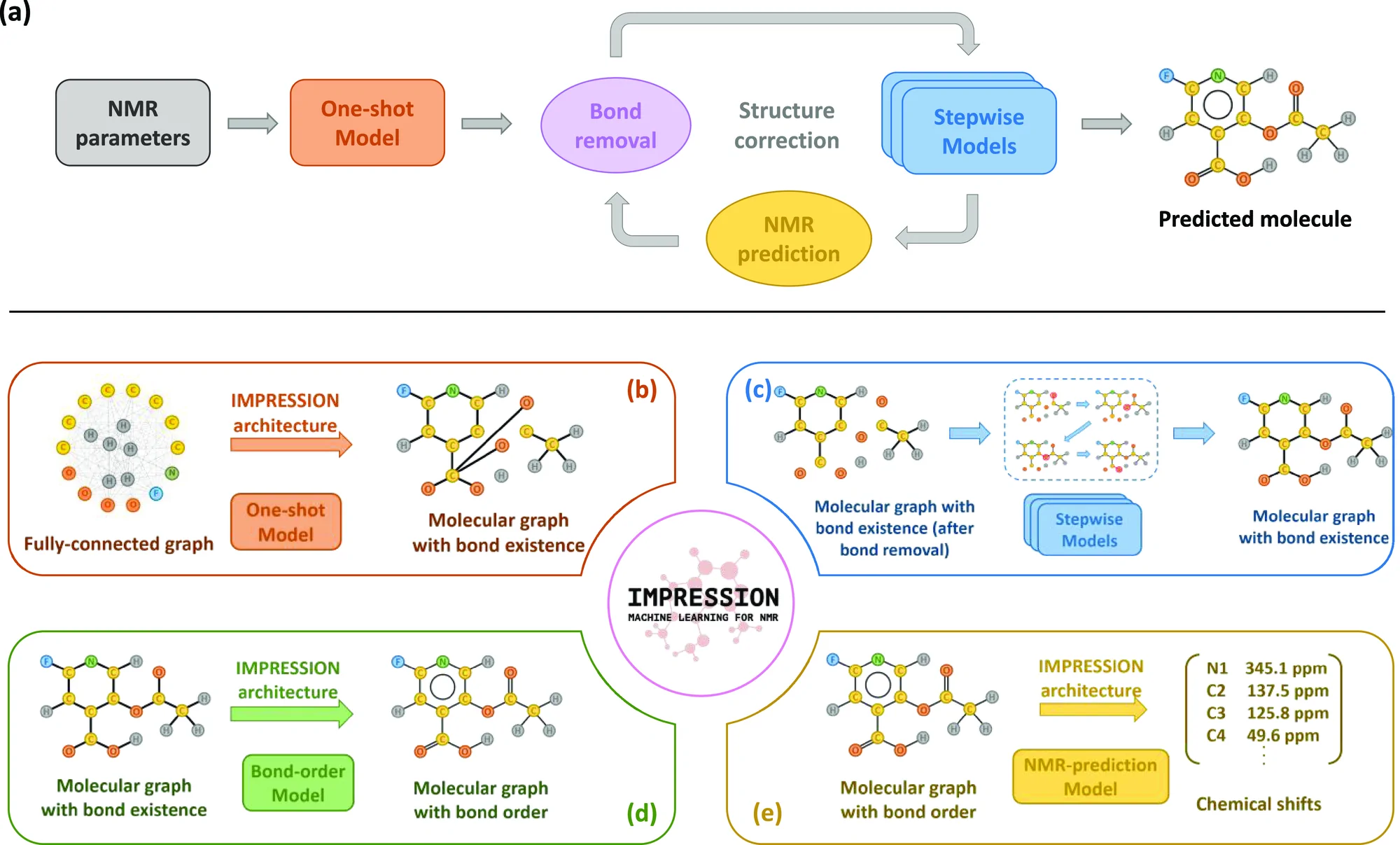
Structure elucidation using the inverse-IMPRESSION framework and the roles of its four component models. (a) Overview of the structure elucidation workflow. (b) One-shot model, which predicts bond connectivity between atoms simultaneously. (c) Stepwise model, which iteratively assigns bonds to partially constructed structures. (d) Bond-order model, which assigns bond orders to structures generated by the one-shot or stepwise models. (e) NMR-prediction model, which predicts chemical shifts for the resulting structures and identifies atoms with incorrect bonding assignments.

### One-Shot Model

The one-shot model (Figure 1b) predicts all bonds within a molecule simultaneously from NMR spectroscopic inputs. Trained on the full-molecule training set, it employs atomic numbers and chemical shifts concatenated as node features and scalar coupling correlations or constants as edge features. These input features are processed through the message-passing scheme of the IMPRESSION graph transformer-based architecture, followed by linear layers applied to each edge and a final sigmoid activation layer, producing a graph in which the updated edge features represent the probabilities of the bond existence between atom pairs. A threshold of 0.5 is applied to convert these probabilities into binary bond predictions to balance the assignment between the presence and absence of bonds.

### Stepwise Model

The stepwise model (Figure 1c) performs iterative bond prediction on molecular fragments generated from earlier-stage outputs. These fragments are obtained either by removing bonds to all heteroatoms following the one-shot model, or by removing bonds around carbon atoms whose predicted chemical shifts (see ‘NMR-Prediction Model’ section below) show large deviations from the experimental input data. At each iteration, the model focuses on a subvalent atom and predicts bond probabilities to all other atoms, thereby identifying the most likely attachment atom within the current fragment(s). Bonds are then added sequentially to the focused atom until no further suitable attachment is found, after which the model proceeds to the next subvalent atom. This process is repeated until all subvalent atoms have been considered, which is equivalent to the generation process of the fragment training subset visualized in Supporting Figure 4.

The stepwise model incorporates the same node and edge features as those of the one-shot model. As it operates on molecular fragments, additional labels are concatenated to the node and edge features, respectively, to indicate which atoms need to be focused on during stepwise iterations. Following the IMPRESSION architecture and a sigmoid activation layer, the model outputs bond probabilities between the focused atom and all of the other atoms in the fragment. The atom with the highest bond probability exceeding a predefined threshold of 0.5 is selected as the attachment atom, and a bond is formed accordingly. If no probabilities exceed this threshold, the model proceeds to focus on the next subvalent atom.

### Bond-Order and NMR-Prediction Models

The bond-order model (Figure 1d) assigns bond orders to structures generated by the one-shot or stepwise models, enabling their use in the subsequent NMR-prediction step. It is applied after each generation step but is not explicitly shown in Figure 1a for clarity. The NMR-prediction model (Figure 1e) then predicts chemical shifts for the resulting structures, and by comparison to the given NMR measurements, it identifies atoms with incorrect bonding assignments to be corrected in the later structure correction stage. Both models were trained on the full-molecule data set; their architectures and input features are described in detail in Supporting Section 1.2.5.

### Multishot Noise-Augmented Molecule Reconstruction

During testing, structural candidates were generated using a multishot noise augmentation strategy to enable Top-k sampling across a distribution of experimental variability in the NMR spectroscopic chemical shift and scalar coupling data. Gaussian noise was added to the input chemical shifts and scalar coupling constants, with standard deviations corresponding to realistic uncertainties between experiment and our simulation method, reflecting the experimental variability in the NMR measurements (arising from differences in solvent, temperature, concentration, etc.) as well as the uncertainty coming from the DFT calculations used to generate the training data (0.3 ppm for ^1^H, 2.5 ppm for ^13^C, and 0.4 Hz for all coupling constants between NMR-active nuclei, unless otherwise stated). Following noise augmentation, edges associated with coupling constants greater than 2 Hz were assigned the corresponding coupling correlation type (see ‘Data set’ section), whereas all others were assigned as uncorrelated (value of 0). Each noise-perturbed input produced a single predicted structure, yielding an ensemble of candidate structures for a given set of NMR parameters. These candidates were subsequently ranked using performance metrics described below.

### Performance Metrics

The performance of the inverse-IMPRESSION framework was evaluated using two metrics: (1) single-prediction accuracy, defined as the fraction of test molecules that were correctly predicted from the input NMR parameters without noise augmentation; and (2) top-k accuracy, defined as the fraction of test molecules in which the correct structure is ranked within the top-k predictions generated via noise augmentation. For experimental data, candidate structures were ranked using the mean absolute error (MAE) between the experimental ^13^C chemical shifts and those predicted for each candidate using forward IMPRESSION-G2.^26^ For simulated data, candidate structures were ranked by the frequency with which a given structure was predicted among all noise-augmented predictions. NOTE: A more informed ranking, such as MAE between simulated chemical shifts for candidates and the true structure, was not used during testing on simulated data to avoid the potential for bias from circular validation, *i.e*., the ranking metric being derived from the same simulation method as used to generate the simulated testing data.

## Results

### One-Shot Prediction and Node Degeneracy

The first stage of the prediction pipeline employed a “one-shot” model (Figure 1b) that aimed to predict all bonds within a molecule simultaneously. When trained and tested on the complete set of theoretically available NMR parameters (NMR Data set A), comprising all ^1^H, ^13^C, ^15^N, ^17^O, and ^19^F chemical shifts together with all pairwise scalar coupling constants, the model achieved excellent performance, correctly reconstructing 99.4% of molecules in the test set (Table 1, entry 1) in single-prediction events, with an average prediction time of 0.18 s per molecule (Supporting Table 2). This result is consistent with our recent proof-of-concept work^25^ on predicting interatomic distances and demonstrates the ability of the inverse-IMPRESSION graph transformer network (GTN) architecture to effectively learn the relationship between NMR spectroscopic data encoded in graph-based representations and underlying molecular structure—in this case reflected by bond probabilities.

**Table 1:** Performance of Inverse-IMPRESSION Models as a Function of the NMR Data Set Ablation^a^

Entry	Model	NMR data set	NMR node input features	NMR edge input features	Single prediction accuracy	Top-1 accuracy	Top-3 accuracy	Top-10 accuracy	Top-200 accuracy
1	One-shot	A	δ^1^H, δ^13^C, δ^15^N, δ^17^O, δ^19^F	*J*_HCNOF–HCNOF_	99.4%	-	-	-	-
2	One-shot	B	δ^1^H, δ^13^C	*J*_CH–H_ correlations	5.3%	-	-	-	-
3	Stepwise	B	δ^1^H, δ^13^C	*J*_CH–H_ correlations	49.6%	74.2%	81.2%	82.7%	82.8%
4	Stepwise with structure correction	B	δ^1^H, δ^13^C	*J*_CH–H_ correlations	53.4%	77.8%	86.4%	88.7%	89.2%

a δ^1^H, δ^13^C, δ^15^N, δ^17^O and δ^19^F are chemical shifts of the corresponding nuclei. *J*_HCNOF–HCNOF_ refers to scalar coupling constants between all pairs of the five nuclei, and *J*_CH–H_ correlations are coupling correlations between C–H and H–H atom pairsa

However, NMR data set A represents an experimentally unrealistic scenario, as many of the included NMR parameters such as ^17^O chemical shifts or very small scalar couplings (below 2 Hz) are generally impractical to measure using routine solution-state NMR methods. To address this, a model was trained under more experimentally realistic conditions, using only commonly measured parameters: ^1^H and ^13^C chemical shifts, together with ^1^H–^1^H and ^1^H–^13^C scalar couplings greater than 2 Hz, which were then converted to coupling correlation labels (NMR Data Set B). Under these conditions, the one-shot model achieved a single-prediction accuracy of only 5.3% (Table 1, entry 2). While the molecule-level prediction accuracy appears low, this corresponds to an edge prediction accuracy of 99.0%, *i.e*., 99.0% of edges were correctly assigned as bonds (or not).

In order to understand the reason for the 5.3% molecular accuracy, despite the 99% edge-level accuracy, we analyzed the one-shot model performance as a function of molecular composition (Supporting Section 2.3.1) using NMR Data set B. This reveals that molecules containing two or more nitrogen atoms or two or more oxygen atoms consistently fail to yield any correct structures. This behavior is also in line with that previously observed^25^ and can be rationalized by the degeneracy of the node/edge information for such heteroatoms; i.e., all atoms of these types contain identical (null) node/edge features. This is demonstrated by a representative failure case in Figure 2. The ground-truth structure, 2-hydroxy-2-methylbutanoic acid, contains three oxygen atoms (hydrogens and bond orders omitted for clarity), each bonded to one of two adjacent carbons (C1 and C2; Figure 2e). While the one-shot model correctly predicts the carbon framework (Figure 2a), it incorrectly assigns all three oxygen atoms to C1 and leaves C2 unbound to any oxygen (Figure 2d). This behavior arises because the corresponding bond existence probabilities show identical values for all three oxygen atoms (66% for bonding to C1 and 33% to C2; Figure 2b,2c). This uniformity reflects the absence of NMR information for all oxygen (as well as N and F) atoms in NMR Data Set B, resulting in identical (null) node and edge features for these atoms in the input graph. Consequently, the model assigns indistinguishable and chemically unrealistic bonding probabilities across the predicted structures.

**Figure 2:**
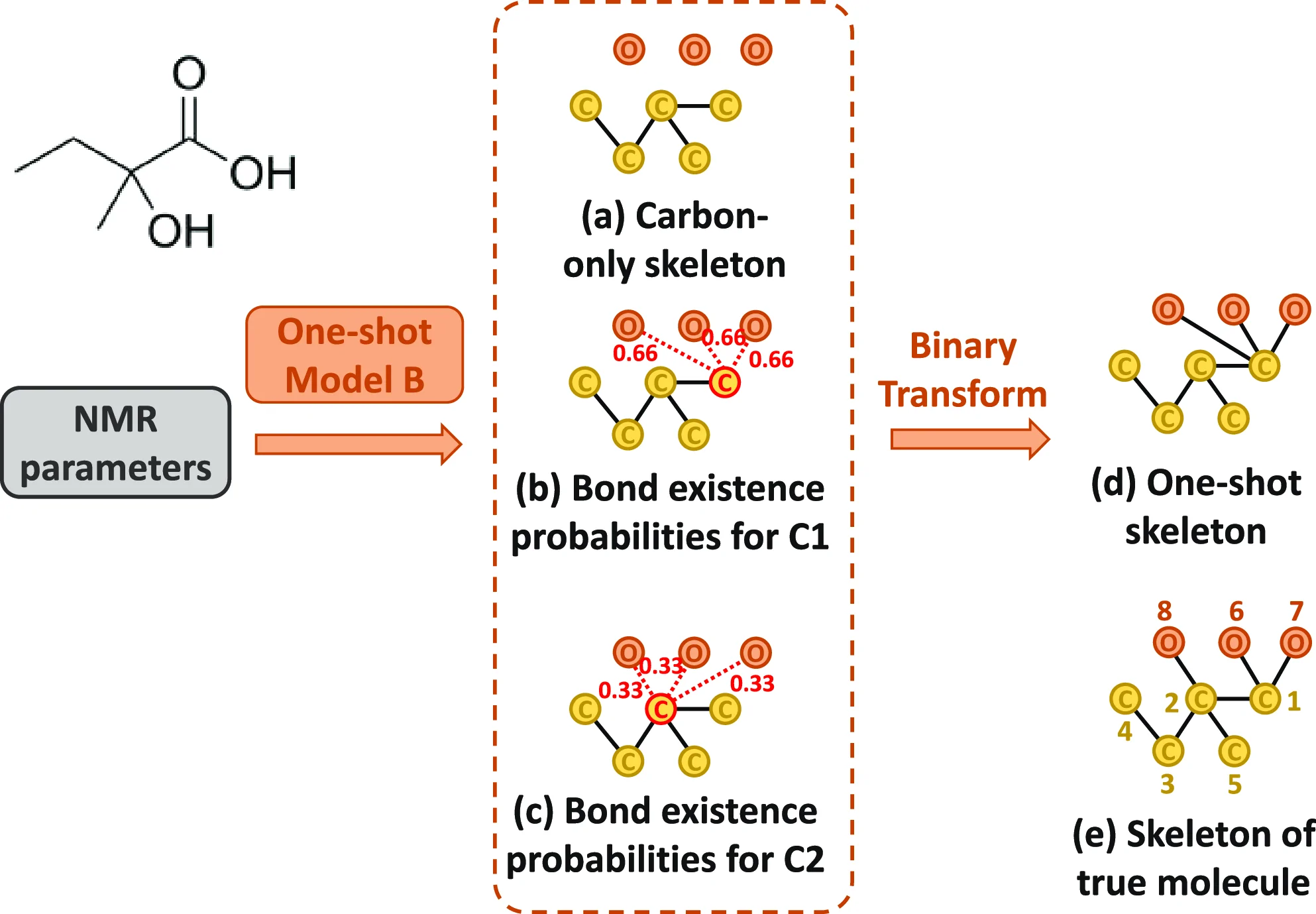
Incorrect prediction of 2-hydroxy-2-methylbutanoic acid by the one-shot model trained on NMR data set B. Predicted bond existence probabilities and corresponding molecular structures are shown (hydrogens and bond orders omitted for clarity). (a) Predicted carbon skeleton, with high bond existence probabilities for C–C and C–H bonds. (b, c) Predicted bond existence probabilities between the oxygen atoms and C1/C2. (d) Predicted molecular structure obtained by binarising the bond existence probabilities from (b) and (c). (e) Carbon–oxygen skeleton of the ground-truth molecule, with carbon and oxygen atoms indexed.

### Structure Correction

To address node degeneracy, a second stepwise stage is introduced to refine the structures generated by the initial one-shot model. Following the initial prediction, all bonds involving degenerate atoms (N, O, and F in NMR Data set B) are removed. A “stepwise” model (Figure 1c) then iteratively focuses on one subvalent atom at a time and predicts bonding probabilities for that focused atom to each other atom in the graph. A bond is then assigned to the edge with the highest predicted probability, and the process is repeated until there is no bond probability predicted to be greater than 0.5, at which point the next focused atom is selected.

This stepwise procedure substantially improves performance, increasing the single-prediction accuracy to 49.6% across the simulated full-molecule test set (Table 1, Entry 3), with an average prediction time of 0.84 s per molecule (Supporting Table 2). Further improvement was achieved through multishot noise augmentation, in which each prediction was repeated 200 times with small perturbations applied to the input spectroscopic data. The resulting candidate structures were then ranked according to their frequency of occurrence across the ensemble of 200 structures. Under these conditions, the combined “one-shot + stepwise” workflow achieved a Top-1 accuracy of 74.2%, with the correct structure appearing within the Top-3 and Top-10 in 81.2% and 82.7% of cases, respectively, and among all the 200 generated candidates in 82.8% of cases.

To correct edges that were misassigned for reasons other than degeneracy, an NMR-prediction stage of structure correction was added. ^13^C chemical shifts are predicted for any fully valent candidate structure output by the stepwise model, and these are compared to the input chemical shift values. Carbon atoms exhibiting large prediction errors (mean absolute error (MAE) > 10 ppm), together with their neighboring N, O, and F atoms, have their bonds removed, after which the stepwise model is again applied to reassign bonds for the resulting subvalent atoms. Incorporation of this further structure correction procedure increased the Top-1 accuracy to 77.8% for NMR Data set B (Table 1, entry 4), with corresponding Top-3, Top-10, and Top-200 accuracies of 86.4%, 88.7%, and 89.2%, respectively. The average prediction time was 1.66 s per molecule for a single noise-augmented input (Supporting Table 2).

Notably, the overall performance of this platform on simulated data sets is robust with increasing molecular size (Figure 3b,c) and provides consistently high Top-k accuracy as a function of molecular complexity, as quantified by the normalized spatial score (nSPS)^31^ (Figure 3d). The ranking method in particular performs well, with top-3 to top-200 accuracies exceeding 95% for molecules containing five or fewer heteroatoms, suggesting that node degeneracy has been substantially mitigated.

**Figure 3:**
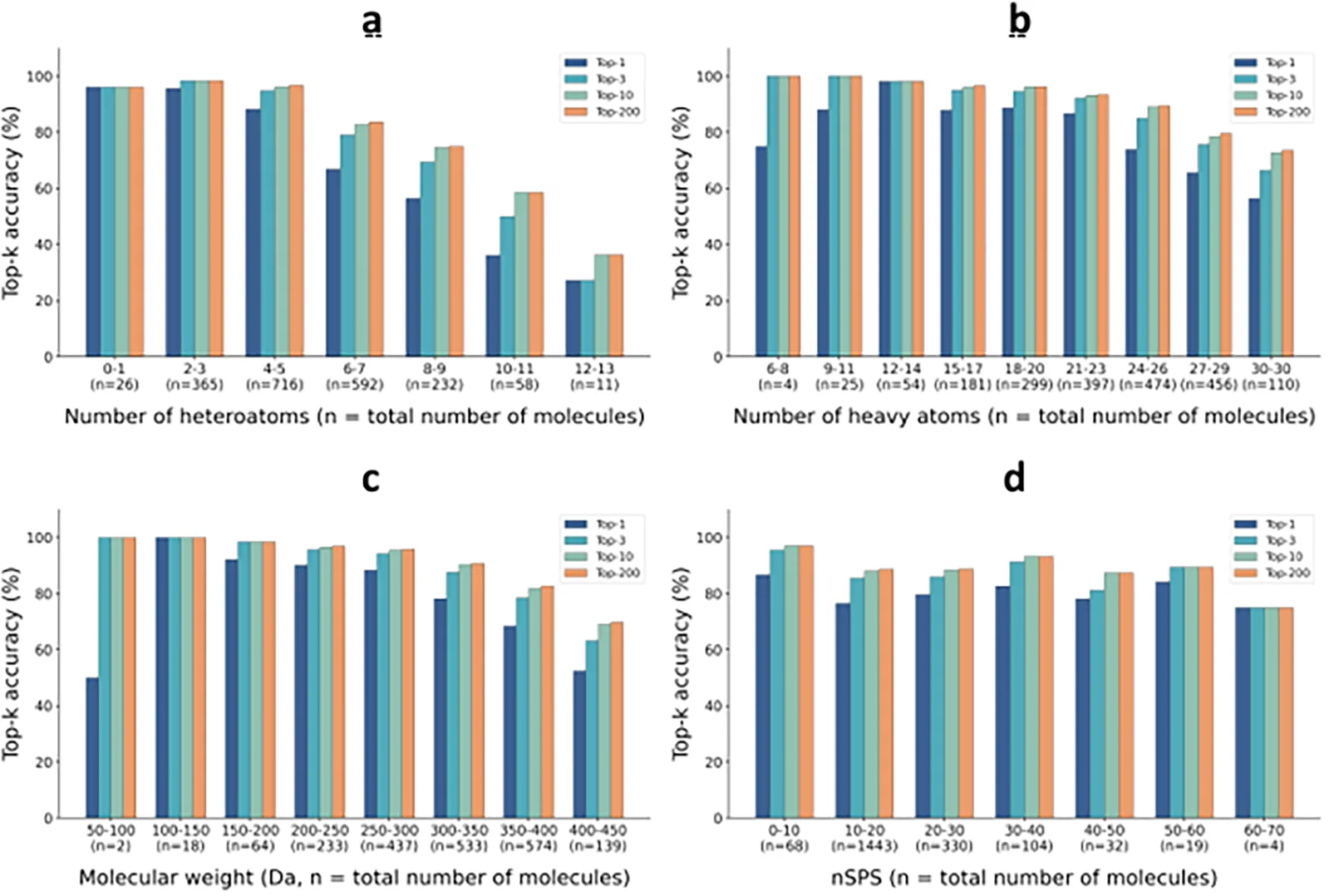
Effect of molecular properties on Top-*k* (*k* = 1, 3, 10 and 200) accuracy after structure correction with NMR data set B. The total number of molecules within each property range is indicated below the corresponding bars. (a) Dependence on heteroatom count (N, O and F). (b) Dependence on heavy atom count (C, N, O and F). (c) Dependence on molecular weight. (d) Dependence on molecular complexity, quantified by normalized spatial score (nSPS).

However, these accuracies decrease to below 60% for molecules with more than ten heteroatoms (Figure 3a). This is attributed to the low density of NMR data available for molecules rich in nitrogen, oxygen, and fluorine, which reduces the information available to inform the bond probability prediction around these atoms, thereby limiting model performance, even when node degeneracy is addressed. This sensitivity to heteroatoms can be reduced further by the inclusion of ^15^N and ^19^F NMR data, which are experimentally accessible but rarely used in complex structure elucidation. This is readily demonstrated by retraining the platform to include these nuclei, resulting in over 80% of Top-3 to Top-200 accuracy on structures containing up to 13 heteroatoms (structure correction model with NMR Data Set C, Supporting Information 2.3.4).

### Performance on Experimental Data

Having established robust performance on simulated data, the complete inverse-IMPRESSION platform was then evaluated on an experimental data set comprising 19 molecules (*M*_R_ = 143.2–608.7 g/mol, nSPS = 9.6–59.2), representative of the types of complex and challenging organic molecular structures which chemists working in pharmaceutical and natural products chemistry may be interested in solving. The experimental NMR data were obtained from the literature^32^ or generated in this work. As shown in Figure 4, 11 structures were correctly identified by the platform. Compounds 1–7 and 11 were recovered using 2000-shot predictions with the same noise parameters as in the test on simulated data above. Where no valid candidate structures were obtained with low mean absolute error (<5 ppm) between the experimental and predicted ^13^C chemical shifts, additional prediction shots or a higher standard deviation of the coupling constant noise were applied to expand the search space. Thus, compound 8 was identified after an additional 5000 shots, and two further compounds (9–10) were found by increasing the standard deviation of the coupling constant noise to explore a greater region of spectroscopic data space. The largest and most complex successful examples include the polycyclic steroids 24-epibrassinolide 7 (*M*_R_ = 480.7 g/mol, *n*SPS = 48.1) and betulin 6 (*M*_R_ = 442.7 g/mol, *n*SPS = 56.5), which, to our knowledge, represent among the most challenging and structurally complex molecules solved using machine learning approaches to date.

**Figure 4:**
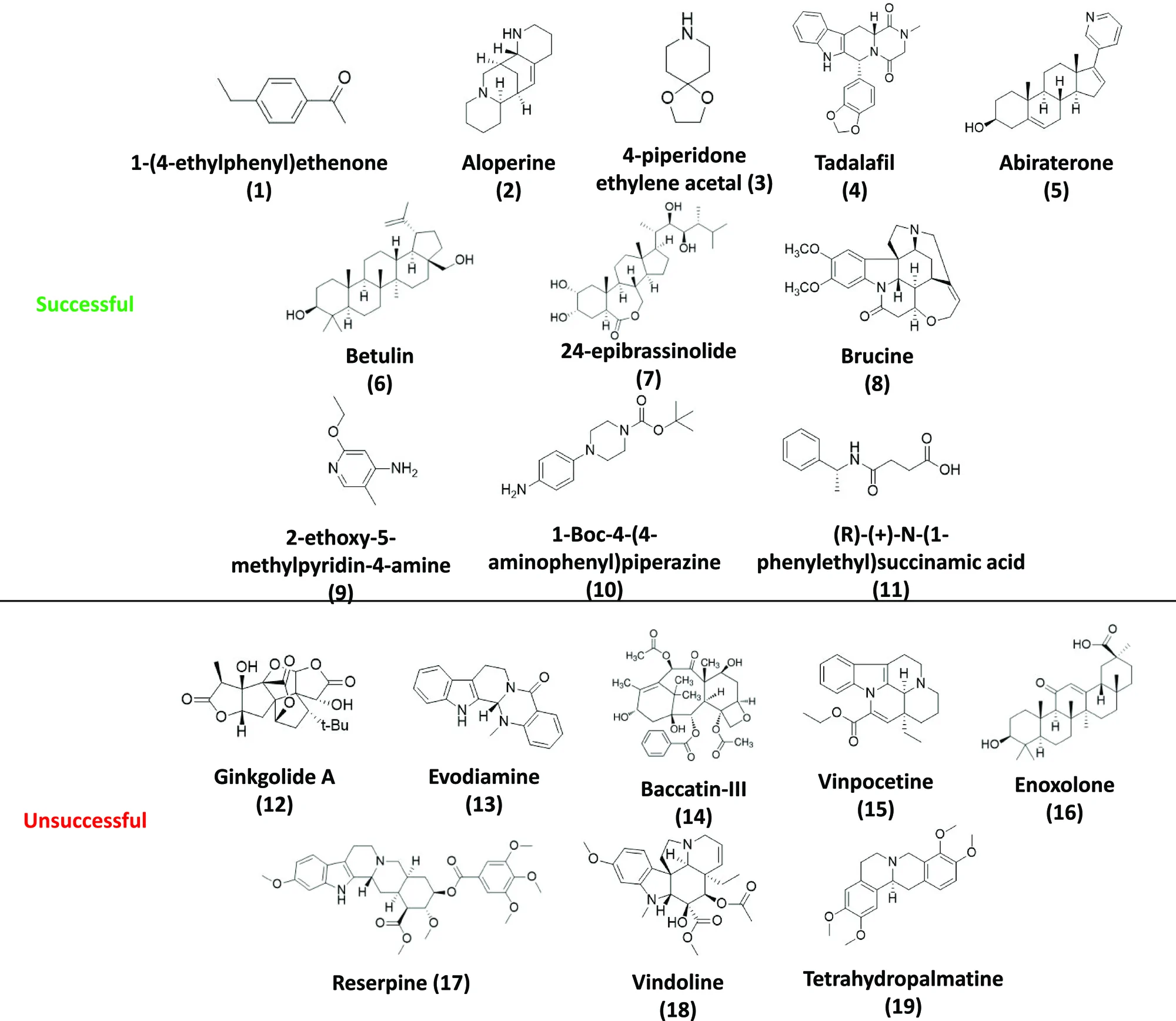
Structures of 19 molecules with experimental NMR spectroscopic data, of which 11 were successfully elucidated and 8 were unsuccessful.

In our experimental testing, correct structures were consistently and reliably identified as Top-1 by ranking according to the MAE between experimental and predicted ^13^C chemical shifts, computed using IMPRESSION-G2 (Supporting Section 2.4.1). Using this criterion, the correct structure was ranked first in all cases where it was generated among the noise-augmented candidates. In each case, the corresponding MAE values for the correct structure were below 4.4 ppm, which is in line with the benchmarked performance of the NMR-prediction tool, IMPRESSION-G2, against experimental data. By contrast, in all eight unsuccessful cases (Compounds 12–19), the incorrect structures exhibited substantially higher MAE values (≥5.3 ppm), enabling clear discrimination from correct predictions (Supporting Section 2.4.2) and highlighting that the MAE ranking criteria offers a substantial indicator of confidence for incorrect predictions made by this machine learning platform.

In line with the performance on simulated data described in Figure 3, accuracy on experimental data was relatively, but not completely, insensitive to molecular size and complexity. Successful cases spanned *M*_R_ = 143.2–480.7 g/mol and *n*SPS = 9.6–56.5, while the unsuccessful cases spanned *M*_R_ = 303.4–608.7 g/mol and *n*SPS = 18.4–59.2. Additional factors influencing prediction success are summarized in Supporting Table 4. As expected, performance decreased when less 2D NMR data were available and when the target structure contained more quaternary-quaternary carbon bonds and heteroatoms. These factors highlight the importance of both the quantity and, importantly, the distribution of NMR spectroscopic information across the molecular structure that is being solved; that is, molecules that contain regions described by minimal NMR information, such as quaternary centers and heteroatom-rich substructures, are inherently more difficult to resolve with NMR data alone. This can also be seen when ablating input data arising from certain experimental NMR methods is done from the input graph. Removing HSQC and HMBC data led to no structures being correctly elucidated. Removing COSY data had less impact, with some structures, particularly those with fewer quaternary centers, still being solvable. These limitations are well recognized in human-led NMR-based structure elucidation: if insufficient NMR spectroscopic connectivity information is available for a substructure, accurate structure prediction becomes challenging for human analysts and unsurprisingly also for this machine learning system as well.

## Conclusions

Herein, we demonstrate that the GTN-based inverse-IMPRESSION platform can be adapted to the task of molecular structure elucidation directly from measurable NMR parameters, including chemical shifts and scalar coupling correlations in two-dimensional NMR spectra. We overcome the limitations of degeneracy for nodes and atoms that have no associated NMR data with an iterative bond prediction strategy. When combined with noise-augmented multishot prediction and frequency ranking, the resulting workflow achieves strong performance on simulated data sets containing only ^1^H and ^13^C NMR information, achieving a Top-1 accuracy exceeding 77%. This simulation-trained model is effectively transferred to testing on experimentally measured NMR data without additional fine-tuning, correctly identifying 11 out of 19 molecules (58%), including complex natural-product-like and synthetic structures that challenge expert chemists to solve. These cutting-edge results demonstrate the feasibility of automated structure elucidation from routine experimental NMR measurements using graph neural network models for molecules that can represent challenging tasks (nSPS > 40, *M*_R_ > 400 Da) for experienced structure elucidation specialists, opening new opportunities for applications across fields where NMR-based structure elucidation is critical.

## Supplementary Material



## Data Availability

All data supporting the findings of this study are available within the article and the Supporting Information. Code associated with this work is publicly available via Zenodo at 10.5281/zenodo.20432239.

## Notes

Model architecture, training procedures, data set construction, computational workflows, and detailed benchmarking results (PDF)
