# Supplementary material for: Inverse-IMPRESSION: A Graph-Based Platform for Molecular Structure Elucidation from Experimental NMR Spectroscopic Properties
Source: J Am Chem Soc. 2026 Oct 31;148(44):48054–62. doi: 10.1021/jacs.6c14119 (PMC13761277; doi:10.1021/jacs.6c14119)
Supplement: Supplementary file 1 [file ja6c14119_si_001.pdf]

Supporting Information

Inverse-IMPRESSION: A Graph-based Platform for Molecular Structure Elucidation from Experimental NMR Spectroscopic Properties

Zheqi Jin[1], Grace Armitage[1], Richard Cox[1], Ben Honoré[1], Mohammad Golbabaee[2] and Craig Butts[1,*]

[1]School of Chemistry, University of Bristol, Cantock's Close, Bristol, BS8 1TS, U.K.

[2]School of Engineering Mathematics and Technology, University of Bristol, Ada Lovelace Building, Tankard's Cl, Bristol BS8 1TW

*Correspondence to: Craig.Butts@bristol.ac.uk

Table of Contents

1 Methods 4

1.1 Hardware and Software Availability 4

1.2 Inverse-IMPRESSION platform 5

1.2.1 Graph Transformer Network architecture 5

1.2.2 Graph Representation 7

1.2.3 Multi-head Attention Mechanism 8

1.2.4 Training elements 9

1.2.4.1 Noise-Augmented Training 9

1.2.4.2 LAMBW Optimiser 9

1.2.4.3 CyclicLR scheduler 9

1.2.4.4 Hyperparameters 9

1.2.5 Inverse-IMPRESSION models 10

1.2.6 Loss Function 11

1.2.6.1 Binary cross-entropy (BCE) loss 11

1.2.6.2 Categorical cross-entropy loss 11

1.2.6.3 L1 loss 11

1.3 Datasets 12

1.3.1 Full-Molecule Training and Test Set 12

1.3.2 Fragment Training Subset 14

2 Results and Discussion 16

2.1 Prediction Performance of Inverse-IMPRESSION Models 16

2.2 Edge-level Analysis on One-Shot Models 19

2.3 Top-k Accuracy Trends for Inverse-IMPRESSION Models 20

2.3.1 One-Shot Model B 20

2.3.2 One-Shot Model C 24

2.3.3 Stepwise Model B With Structure Correction 28

2.3.4 Stepwise Model C With Structure Correction 32

2.4 Application of Inverse-IMPRESSION on experimental molecules 36

2.4.1 Correct Predictions (Top MAE Rank) 37

2.4.2 Incorrect Predictions 43

2.4.3 Properties of Experimental molecules 51

3 Figures for dataset distributions 52

4 Experimental NMR Spectra 59
4.1 1-(4-ethylphenyl)ethenone 59
4.2 4-piperidone ethylene acetal 62
4.3 2-ethoxy-5-methylpyridin-4-amine..65
4.4 1-Boc-4-(4-aminophenyl)piperazine..68
4.5 (R)-(+)-N-(1-phenylethyl)succinamic acid 71
References 74

1 Methods

1.1 Hardware and Software Availability

All computations were performed on a single NVIDIA GH200 Grace Hopper Superchip hosted on the Isambard-AI Phase 2 high-performance computing system, running SUSE Linux Enterprise Server 15 SP6. The superchip integrates a 72-core NVIDIA Grace CPU and an NVIDIA Hopper H100 Tensor Core GPU, providing 115 GB of CPU memory and 96 GB of GPU memory.

The computational environment was managed using Conda (v25.7.0) with Python 3.12.11. GPU acceleration was provided by NVIDIA CUDA (v12.6). The main software dependencies included Numpy (v2.3.3), Pandas (v2.3.2), RDKit (v2025.03.6), Scipy (v1.16.2), PyTorch (v2.6.0) and PyTorch Geometric (v2.6.1).

Three in-house Python packages were employed in this study. IMPRESSION-G2,[1] previously developed and reported in our published work, was used to predict chemical shifts and scalar coupling constants for 3D molecular structures, thus generating the training and test datasets used in this work. Efficient Molecular Storage (EMS) was developed for molecular and nuclear magnetic resonance (NMR) data processing and storage and was additionally used for conformer generation and geometry optimization via RDKit.[2] Inverse-IMPRESSION was developed based on IMPRESSION-G2, aiming at extending the original framework to multiple learning tasks. The four models investigated in this study (one-shot, stepwise, bond-order, and NMR-prediction models) were all trained using the Inverse-IMPRESSION platform.

The code for EMS and inverse-IMPRESSION, and the SDF files of 19 molecules with experimental NMR parameters are available at: https://github.com/Buttsgroup and https://doi.org/10.5281/zenodo.20432239

1.2 Inverse-IMPRESSION platform

1.2.1 Graph Transformer Network architecture

The Graph Transformer Network (GTN) architecture of inverse-IMPRESSION is an adapted version of the solution presented in the CHAMMPS Kaggle competition[3] and deployed in IMPRESSION-G2.[1] All models used in inverse-IMPRESSION platform share the same GTN architecture but differ in their input and output node/edge features, and therefore have distinct learned weights and biases. The layers of the GTN architecture are described as follows (Supplementary Figure 1):

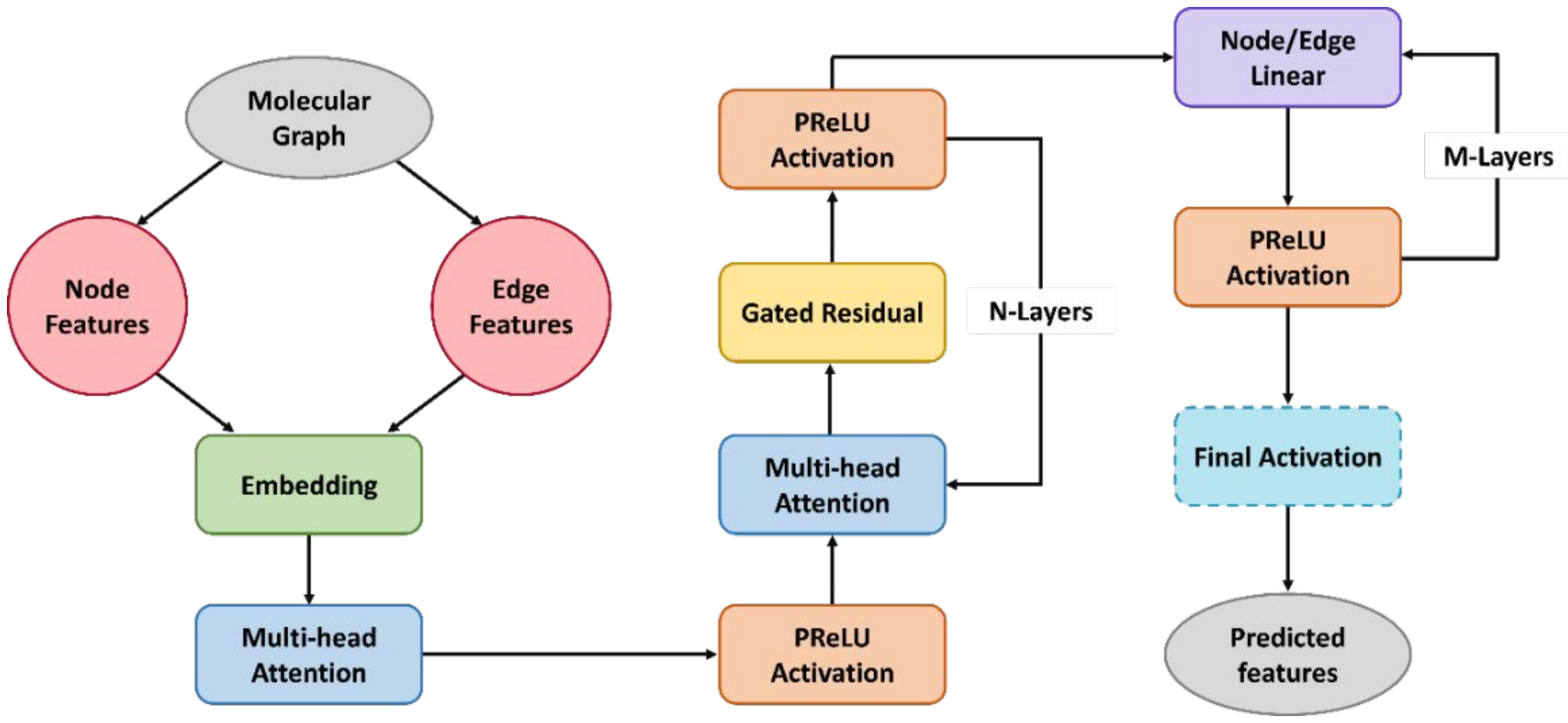

Supplementary Figure 1. The GTN architecture of the inverse-IMPRESSION platform

1. **Molecular Graph:** The input molecular structure or NMR spectroscopic data is represented as a fully-connected graph (Supplementary Figure 2), in which atoms correspond to nodes and atom pairs correspond to edges. Each node and edge is associated with a feature vector encoding the relevant atomic or pairwise information. The input and output node/edge features for each model are discussed in Supplementary Section 1.2.5.

2. **Embedding Layer:** Embeddings provide continuous vector representation for discrete data such as categorical labels and words, which are not directly usable by neural networks. These representations are learnable and can be updated during training to capture task-specific relationships. The node and edge features that are transformed into embeddings for each model are discussed in Supplementary Section 1.2.5.

3. **Multi-head Attention Layer:** After categorical features are transformed into embeddings and all node and edge features are concatenated, they are passed to the multi-head attention layer, which is described in Supplementary Section 1.2.3.

4. **PReLU Activation Layer:** An activation layer introduces non-linearity to the model, enabling it to learn complex patterns and relationships within the data. In inverse-IMPRESSION, PReLU[4] (Parametric Rectified Linear Unit) is used as the activation function following the multi-head attention layers, gated residual layers and linear layers. The PReLU function is defined as:

$$PReLU(x) = max(0,x) + \boldsymbol{a} \cdot min(0,x) \quad \text{(S1)}$$

where $\boldsymbol{a}$ is a learnable parameter that controls the slope of the negative part of the function.

5. **Gated Residual layer:** The concept of the gated residual layer builds on the gating mechanism introduced in gated recurrent units[5] (GRUs) and has been extended to graph neural networks (GNNs). These layers incorporate learnable gates to control the flow of information between representations from previous and current layers, enabling the model to adaptively determine which features should be retained or discarded. In inverse-IMPRESSION, the gated residual layer adopts an update-gate mechanism inspired by GRUs, which is defined as follows:

$$h_t = z_t \odot \tilde{h}_t + (1 - z_t) \odot h_{t-1} \tag{S2}$$

$$\tilde{h}_t = f(h_{t-1}) \tag{S3}$$

$$z_t = \sigma(\boldsymbol{W}_t h_{t-1} + \boldsymbol{U}_t \tilde{h}_t + \boldsymbol{b}_t) \tag{S4}$$

where h_t is the output node or edge representation of the current hidden layer, h_{t-1} is the representation of the previous hidden layer, and $\odot$ denotes element-wise multiplication. The candidate representation $\tilde{h}_t$ is computed by applying a transformation function f to h_{t-1}. The function f represents a message passing neural network (MPNN), which is implemented as a multi-head attention layer in inverse-IMPRESSION. The update gate z_t is calculated by applying a sigmoid function σ to a linear transformation of both h_{t-1} and $\tilde{h}_t$, producing a gating matrix with values between 0 and 1. Here, $\boldsymbol{W}_t$ and $\boldsymbol{U}_t$ are learnable weight matrices and $\boldsymbol{b}_t$ is a learnable bias vector.

6. **Node/Edge Linear layer:** The linear layer is a fundamental component of all neural networks. It performs a linear transformation on the input features:

$$y = \boldsymbol{W}x + \boldsymbol{b} \tag{S5}$$

where x is the input feature, y is the output feature, $\boldsymbol{W}$ is a learnable weight, and $\boldsymbol{b}$ is a learnable bias vector.

7. **Final Activation Layer:** The final activation layer is the last layer in the inverse-IMPRESSION models and is used to map the output features to task-specific values. The sigmoid function is employed as the final activation layer in the one-shot and stepwise models to predict bond existence probabilities and atom connectivity probabilities. The softmax function is applied in the bond-order model to predict bond order probabilities across five classes: no bond, single bond, double bond, triple bond, and aromatic bond. No final activation layer is applied in the NMR-prediction model.

   The sigmoid function maps values to a range between 0 and 1 and can be converted into binary outputs using a threshold of 0.5. It is therefore commonly used in binary classification tasks (NB: tests of alternatve thresholds between 0.1 and 0.9 did not substantively change the accuracy of the models). The sigmoid function is defined as:

$$\sigma(x) = \frac{1}{1 + e^{-x}} \tag{S6}$$

   The softmax function maps a vector of values to a probability distribution over multiple classes, such that the outputs are non-negative and sum to one. The resulting probability distribution can then be converted into a class prediction, typically by selecting the class with the highest probability. It is therefore commonly used in multi-class classification tasks. The softmax function (K classes) is defined as:

$$softmax(x_i) = \frac{exp(x_i)}{\sum_{j=1}^{K} exp(x_j)} \tag{S7}$$

1.2.2 Graph Representation

In molecular modelling, graph representations are particularly useful as they naturally mirror the topological structure of molecules. Atoms are represented as nodes and are connected by edges, which can correspond to chemical bonds or any type of atomic interactions. Each node and edge is associated with a feature vector, allowing the encoding of relevant information such as atom type, chemical shift, bond order, and scalar coupling correlations. In the inverse-IMPRESSION platform, the graph representation is extended to a fully-connected graph, where every pair of atoms is connected by an edge (Supplementary Figure 2). This design is motivated by the structure elucidation task, in which the molecular structure is unknown. Inverse-IMPRESSION aims to infer which edges correspond to true chemical bonds.

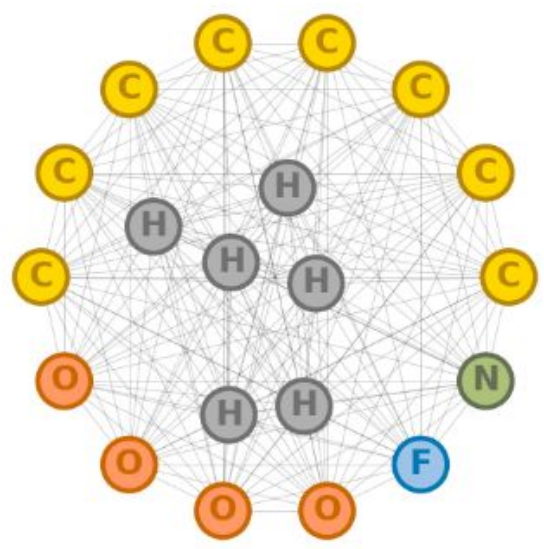

Supplementary Figure 2. A fully-connected graph with 20 nodes (6 H atoms, 8 C atoms, 1 N atom, 4 O atoms, and 1 F atom). Each pair of atoms (nodes) is connected by an edge (grey lines).

1.2.3 Multi-head Attention Mechanism

All four models in the inverse-IMPRESSION platform are built according to the Graph Transformer Network (GTN) architecture,[6] which integrates the Transformer framework[7] into GNNs. The Transformer architecture relies on the attention mechanism, allowing the model to weight the importance of information aggregated from neighbouring nodes and edges, and thus capture relational information within the graph. The multi-head attention mechanism extends this capability by employing multiple independent attention heads, enabling different aspects of node and edge features, as well as their relationships, to be captured by different heads.

The multi-head attention mechanism in inverse-IMPRESSION is implemented as follows:

$$z_{uv}^{(h)} = \left[h_u^{(t-1,h)} \| e_{uv}^{(t-1,h)} \| h_v^{(t-1,h)} \right] \tag{S8}$$

$$\alpha_{uv}^{h} = softmax_u\left(\boldsymbol{w}^{(h)^{\mathrm{T}}} z_{uv}^{h} \right) \tag{S9}$$

$$\tilde{h}_u^{(h)} = \alpha_{uv}^{(h)} \cdot h_u^{(t-1,h)} \tag{S10}$$

$$m_{u\to v}^{(h)} = \tilde{h}_u^{(h)} \odot e_{uv}^{(t-1,h)} \tag{S11}$$

$$m_v^{(h)} = \sum_{u\in\mathcal{N}(v)} m_{u\to v}^{(h)} \tag{S12}$$

$$h_v^{(t,h)} = h_v^{(t-1,h)} + m_v^{(h)} \tag{S13}$$

$$e_{uv}^{(t,h)} = \boldsymbol{W}_E^{(h)}\left[h_u^{(t,h)} \| e_{uv}^{(t-1,h)} \| h_v^{(t,h)} \right] \tag{S14}$$

1. **Attention calculation (Supplementary Eqs. 8-9):** The source node features $h_u^{(t-1,h)}$, edge features $e_{uv}^{(t-1,h)}$ and target node features $h_v^{(t-1,h)}$ in the $(t-1)$-th layer and h-th attention head are first concatenated to form a triplet vector $z_{uv}^{(h)}$ for each edge (u,v). The attention weight α_{uv}^{h} is then computed by applying a linear transformation with a learnable weight vector $\boldsymbol{w}^{(h)}$ to z_{uv}^{h}, followed by a softmax operation. The softmax operation is applied over the source nodes u for each target node v, ensuring that the attention weights sum to 1 across all neighboring nodes of v.

2. **Message function (Supplementary Eqs. 10-11):** The source node features $h_u^{(t-1,h)}$ are weighted by the attention weights α_{uv}^{h} to form a weighted node feature vector $\tilde{h}_u^{(h)}$. The message $m_{u\to v}^{(h)}$ is then computed by multiplying the weighted node feature vector $\tilde{h}_u^{(h)}$ with the edge feature $e_{uv}^{(t-1,h)}$.

3. **Aggregation function (Supplementary Eq. 12):** The messages $m_{u\to v}^{(h)}$ from all neighboring nodes $\mathcal{N}(v)$ are aggregated by sum operation to form a single message $m_v^{(h)}$ for the target node v.

4. **Node update function (Supplementary Eq. 13):** The target node feature $h_v^{(t,h)}$ is updated by adding the aggregated message $m_v^{(h)}$ to the target node feature $h_v^{(t-1,h)}$ in the previous layer.

5. **Edge update function (Supplementary Eq. 14):** The edge feature $e_{uv}^{(t,h)}$ is updated by applying a linear transformation with a learnable weight matrix $\boldsymbol{W}_E^{(h)}$ to the concatenated vector. This allows the edge features to be updated based on the new node features after the attention mechanism.

1.2.4 Training elements

1.2.4.1 Noise-Augmented Training

Random Gaussian noise was added to the chemical shifts and scalar coupling constants at each training epoch for both the one-shot and stepwise models to improve robustness. The standard deviations applied to the chemical shifts were 0.3 ppm for ^{1}H, 2.5 ppm for ^{13}C, 7.5 ppm for ^{15}N, and 7.5 ppm for ^{19}F nuclei. For all scalar coupling constants, a standard deviation of 0.4 Hz was used. Coupling constants were subsequently converted into integer-encoded scalar coupling correlations using a threshold of 2 Hz. The encoding scheme was defined as follows: *^{1}H-^{1}H* COSY = 1, *^{1}H-^{13}C* HSQC = 2, *^{1}H-^{13}C* HMBC = 3, *^{1}H-^{15}N* HSQC = 4, *^{1}H-^{15}N* HMBC = 5, *^{19}F-^{13}C* HSQC = 6, and *^{19}F-^{13}C* HMBC = 7.

1.2.4.2 LAMBW Optimiser

The LAMBW (Layer-wise Adaptive Moments optimiser for Batching training with Weight decay) optimiser[8] is used to improve the convergence speed and training stability when the inverse-IMPRESSION models are trained on large datasets. The weight decay mechanism is incorporated in the optimiser to prevent overfitting by penalizing large weights, which helps the model generalise better to unseen data.

1.2.4.3 CyclicLR scheduler

The CyclicLR (Cyclic Learning Rate) scheduler[9] is used to vary the learning rate between a lower and an upper bound during training. The learning rate periodically increases and decreases over a fixed number of epochs, which helps the models escape local minima and saddle points in the loss landscape. In the inverse-IMPRESSION platform, the CyclicLR scheduler is applied for a single cycle, after which the learning rate is fixed at the lower bound.

1.2.4.4 Hyperparameters

Hyperparameters are predefined settings that control the training process and model architecture. The four inverse-IMPRESSION models share the same tuneable hyperparameters:

- **Batch Size**: Number of molecular graphs per training iteration, set to**15**.
- **Epochs**: Total number of times the entire training dataset passes over the model, set to **100**.
- **Attention Heads**: Number of attention heads in the multi-head attention layer, set to **16**.
- **Gated Residual Layers**: Number of gated residual layers to update node and edge features, set to **6**.
- **Embedding Dimension**: Dimension of the atom and edge embeddings, set to **48**.
- **Weight Decay**: Weight decay factor in the LAMBW optimiser, set to **0.05**.
- **Minimum Learning Rate**: Lower bound of learning rate in the CyclicLR scheduler, set to **0.001**.
- **Maximum Learning Rate**: Upper bound of learning rate in the CyclicLR scheduler, set to **0.01**.
- **CyclicLR Up Epochs**: Number of epochs the learning rate increases in the CyclicLR scheduler, set to **15**.
- **CyclicLR Down Epochs**: Number of epochs the learning rate decreases in the CyclicLR scheduler, set to **15**.

1.2.5 Inverse-IMPRESSION models

The four models of inverse-IMPRESSION platform are introduced in the main text. Their key features are summarised in Supplementary Table 1.

Supplementary Table 1. Summary of four inverse-IMPRESSION models

Model	One-shot	Bond-order	Stepwise	NMR-prediction
**Input Node Feature**	• atom type<br>• chemical shift	• atom type	• atom type<br>• chemical shift<br>• atom-in-fragment label	• atom type
**Input Edge Feature**	• edge type<br>• scalar coupling correlation	• edge type<br>• bond existence	• edge type<br>• scalar coupling correlation<br>• bond-in-fragment label	• edge type<br>• bond order
**Output Node Feature**	/	/	• atom connectivity probability	• chemical shift
**Output Edge Feature**	• bond existence probability	• bond order probability	/	/
**Final Activation Layer**	Sigmoid	Softmax	Sigmoid	/
**Loss Function**	Binary cross-entropy	Categorical cross-entropy	Binary cross-entropy	L1 loss

The features to be embedded are summarised below:

- **Atom type**: encoded by atomic number (H = 1, C = 6, N = 7, O = 8, F = 9)
- **Edge type**: integer-encoded by atom-pair category (H-H = 0, C-H = 1, C-C = 2, N-H = 3, N-C = 4, N-N = 5, O-H = 6, O-C = 7, O-N = 8, O-O = 9, F-H = 10, F-C = 11, F-N = 12, F-O = 13, F-F = 14)
- **Scalar coupling correlation**: integer-encoded by 2D NMR peaks (^{1}H-^{1}H COSY = 1, ^{1}H-^{13}C HSQC = 2, ^{1}H-^{13}C HMBC = 3, ^{1}H-^{15}N HSQC = 4, ^{1}H-^{15}N HMBC = 5, ^{19}F-^{13}C HSQC = 6, and ^{19}F-^{13}C HMBC = 7)
- **Bond existence**: binary-encoded bond indicator (0 = non-bond, 1 = bond)
- **Atom-in-fragment label**: categorical indicator of atom state (0 = outside the molecular fragment, 1 = within the molecular fragment, 2 = focused atom)
- **Bond-in-fragment label**: binary-encoded bond indicator (0 = outside the molecular fragment, 1 = within the molecular fragment)
- **Bond order**: categorical bond type label (0 = non-bond, 1 = single, 2 = double, 3 = triple, 4 = aromatic)

1.2.6 Loss Function

The loss function is used to quantify the discrepancy between the predicted values and the ground truth, thereby guiding the training process. The choice of loss function depends on the specific task and the final activation layer of each model.

1.2.6.1 Binary cross-entropy (BCE) loss

Binary cross-entropy (BCE) loss is applied in the one-shot and stepwise models for the binary classification task. The BCE loss is defined as:

$$\mathcal{L} = -\frac{1}{N}\sum_{i=1}^{N}\left(y_i log(\hat{y}_i) + (1 - y_i) log(1 - \hat{y}_i)\right) \quad \text{(S15)}$$

where N denotes the number of edges (one-shot model) or nodes (stepwise model) in a batch, y_i is the ground-truth label (0 or 1) for edge or node i, and $\hat{y}_i$ denotes the predicted probability of bond existence (one-shot model) or atom connectivity (stepwise model). This loss function computes the average loss over all edges or nodes within a single batch.

1.2.6.2 Categorical cross-entropy loss

Categorical cross-entropy loss is applied in the bond-order model for the multi-class classification task. It is defined as:

$$\mathcal{L} = -\frac{1}{N}\sum_{i=1}^{N}\sum_{k=1}^{K} y_{i,k} log\left(\hat{y}_{i,k}\right) \quad \text{(S16)}$$

where N denotes the number of edges, K is the number of bond types, $y_{i,k}$ is the ground-truth label of bond order (0 or 1) and $\hat{y}_{i,k}$ denotes the predicted probability for each bond type. This loss function computes the average loss over all edges within a single batch.

1.2.6.3 L1 loss

L1 loss (mean absolute error) is applied in the NMR-prediction model to measure the absolute difference between the predicted and ground-truth chemical shift values. It is defined as

$$\mathcal{L} = \frac{1}{N}\sum_{i=1}^{N} | y_i - \hat{y}_i | \quad \text{(S17)}$$

where N denotes the number of nodes, y_i is the ground-truth chemical shift and $\hat{y}_i$ is the predicted chemical shift. This loss function computes the average loss over all nodes within a single batch.

1.3 Datasets

1.3.1 Full-Molecule Training and Test Set

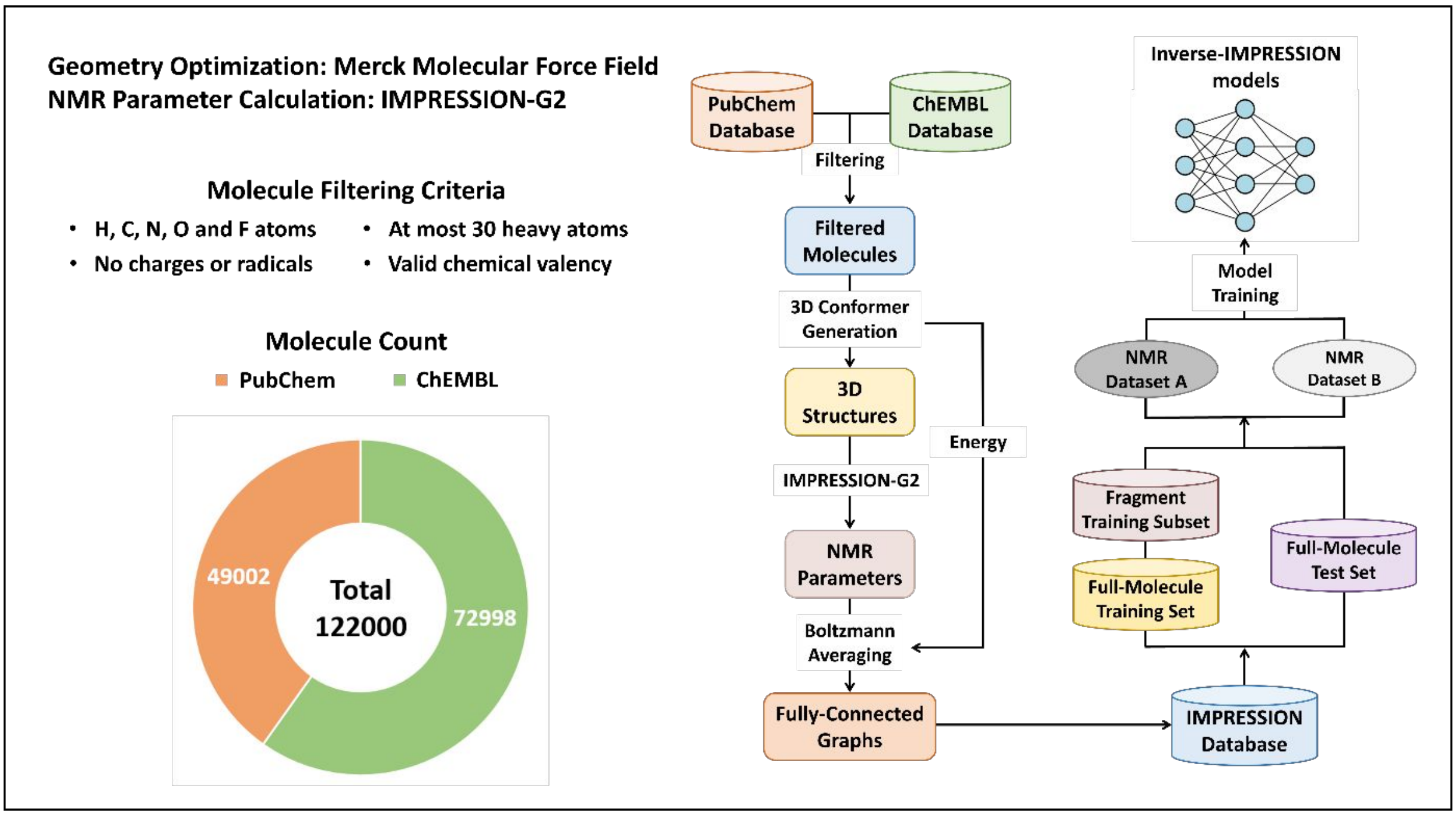

Supplementary Figure 3. Workflow of generating molecular graph datasets for training inverse-IMPRESSION models

The molecules of inverse-IMPRESSION dataset were derived from the public PubChem[10] and ChEMBL[11] databases, and filtered according to the following criteria: (1) containing only H, C, N, O and F atoms; (2) comprising at most 30 heavy atoms; (3) having no charges, radicals or disconnected fragments; and (4) satisfying standard chemical valency rules for all atoms. A final dataset of 122,000 organic molecules was obtained, including 72,998 molecules from the PubChem database and 49,002 molecules from the ChEMBL database. Details are shown in Supplementary Figure 3.

A total of 100 conformers were generated and optimised for each molecule using the Merck Molecular Force Field[12] (MMFF) as implemented in RDKit with default settings. The conformer energies were also calculated using MMFF. The NMR parameters (chemical shifts and scalar coupling constants) of each conformer were predicted using the IMPRESSION-G2 model.[1] The final NMR parameters for each molecule were obtained as Boltzmann-weighted averages over all conformers based on their MMFF energies.

The molecules together with their NMR parameters were processed using our in-house public package, Efficient Molecular Storage (EMS), and stored in DataFrames.[13] They were then transformed into fully-connected graphs using the PyTorch Geometric package,[14] in which each atom was represented as a node and every pair of atoms was connected by an edge. These graph representations were used as inputs to the inverse-IMPRESSION models with customised node and edge features.

The inverse-IMPRESSION dataset was split into a training set of 120,000 molecules (4,974,382 nodes and 107,317,458 edges) and a test set of 2,000 molecules (86,645 nodes and 1,923,615 edges). To distinguish these datasets from the fragment training subset used for training the stepwise model, the inverse-IMPRESSION training and test sets are referred to as the full-molecule training and test sets. The diversity of the molecules can be approximated by visualizing the distributions of molecular weight, atom counts, ring counts, cLogP value, nSPS value and $^{1}H/^{13}C/^{15}N/^{19}F$ chemical shifts, as shown in

Supplementary Section 3. The cLogP (calculated logarithm of partition coefficient) value measures molecule's lipophilicity, which is calculated using the Wildman–Crippen fragment-based model[15] as implemented in RDKit. The nSPS[16] (normalised spacial score) value reflects the 3D structural complexity of a molecule, which is calculated using the SpacialScore module implemented in RDKit.

1.3.2 Fragment Training Subset

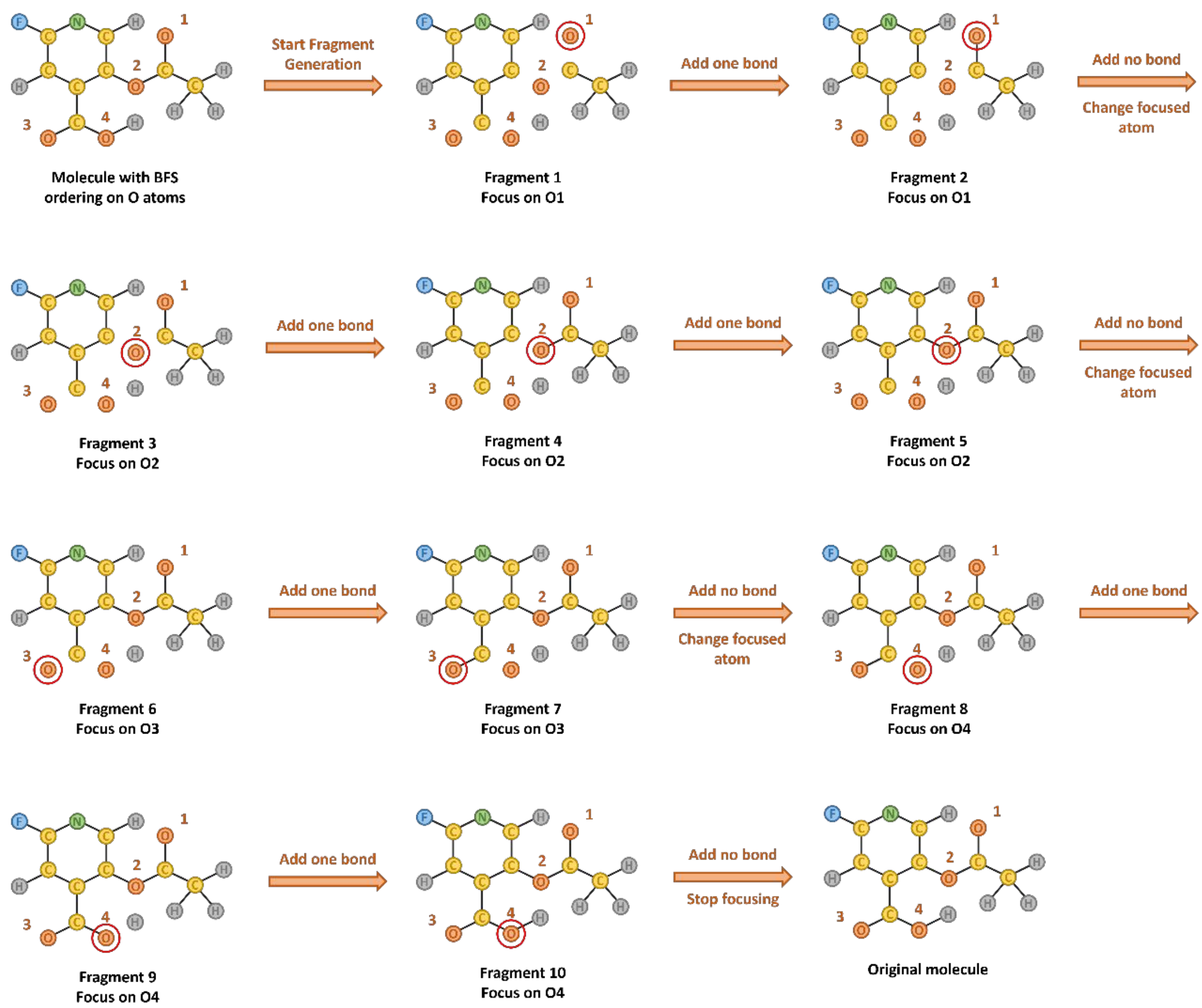

Supplementary Figure 4. The fragment generation process according to the BFS ordering of O atoms. The atom with a red circle is the focused atom in each step.

To train the stepwise model, which predicts the next attachment atom given the current molecular fragment, a subset of 60,000 molecules with no less than 2 oxygen atoms was randomly sampled from the full-molecule training dataset. Two molecular fragments were randomly generated for each molecule using breadth-first search (BFS) algorithm, yielding a fragment training subset of 120,000 molecular fragments.

The BFS algorithm orders graph nodes based on their path distance from a starting node. In the inverse-IMPRESSION platform, BFS is extended to rank NMR-silent atoms by their distance from the NMR-active molecular fragment predicted by the one-shot model. For NMR Dataset B, the BFS algorithm orders the N, O and F atoms by their path distance from the NMR-active fragments composed of H and C atoms. For NMR Dataset C, the BFS algorithm orders the O atoms by their path distance from the NMR-active fragments composed of H, C, N, and F atoms.

Supplementary Figure 4 illustrates a molecule with one BFS ordering of O atoms and the corresponding fragment generation process. The fragment generation starts from the molecular fragment consisting of H, C, N, and F atoms and proceeds by iteratively adding bonds to the growing fragment following the BFS order. A *focus* mechanism is introduced to iterate over all equivalent atoms (O atoms in this example) and determine whether to continue adding bonds to the currently focused atom. For example, Fragment

1 is the initial molecular fragment with focus on the first oxygen atom O1. Fragment 2 is generated by adding a bond between O1 and a carbon atom according to the ground truth. Since O1 has no bonds to add, Fragment 3 retains the same structure but shifts the focus to the next oxygen atom O2. Fragment 4 is then generated by adding a bond between O2 and a carbon atom. The bond-adding and re-focusing steps are repeated until all oxygen atoms have been processed.

2 Results and Discussion

2.1 Prediction Performance of Inverse-IMPRESSION Models

This section summarises the prediction performance of three inverse-IMPRESSION models: (1) one-shot model; (2) stepwise model; and (3) stepwise model with structure correction. Three NMR datasets were used to train the models, representing different scenarios. In addition to NMR Datasets A and B discussed in the main text, NMR Dataset C was included to incorporate NMR data for ^{1}H, ^{13}C, ^{15}N, and ^{19}F. The details of the three NMR datasets are summarised below:

- **NMR Dataset A**: represents a control scenario where NMR chemical shifts and scalar coupling constants (rather than the coupling correlations used in NMR Dataset B and C) are available for all ^{1}H, ^{13}C, ^{15}N, ^{17}O and ^{19}F nuclei, including those that are not experimentally observable and measurable. Although idealised and unrealistic, this setting provides an estimate of inverse-IMPRESSION platform's upper bound of reconstruction accuracy.

- **NMR Dataset B**: represents a scenario in which only the most commonly used NMR experiments are available. It includes chemical shifts for ^{1}H and ^{13}C nuclei and scalar coupling correlations for H-H and C-H atom pairs, corresponding to COSY, HSQC, and HMBC spectra. A threshold of 2 Hz was applied to filter the weak peaks.

  For the stepwise model trained with NMR Dataset B, all bonds involving N, O, and F atoms are removed for the structures generated by the initial one-shot model. The stepwise model then reassigns bonds for these atoms to reconstruct the molecular structure. For the stepwise model with structure correction, additional bond removal is performed for carbon atoms exhibiting large prediction errors (mean absolute error, MAE > 10 ppm) and their neighbouring N, O, and F atoms. The stepwise model subsequently reassigns bonds for these atoms to correct and complete the structure.

- **NMR Dataset C**: represents a scenario with comprehensive experimentally accessible NMR data. It includes chemical shifts for ^{1}H, ^{13}C, ^{15}N, and ^{19}F nuclei, as well as scalar coupling correlations for H-H, C-H, N-H, and F-C atom pairs, corresponding to peaks from ^{1}H-^{1}H COSY, ^{1}H-^{13}C HSQC, ^{1}H-^{13}C HMBC, ^{1}H-^{15}N HSQC, ^{1}H-^{15}N HMBC, ^{19}F-^{13}C HSQC, and ^{19}F-^{13}C HMBC spectra. A threshold of 2 Hz was applied to filter the weak peaks.

  For the stepwise model trained with NMR Dataset C, all bonds involving O atoms are removed for the structures generated by the initial one-shot model. The stepwise model then reassigns bonds for these atoms to reconstruct the molecular structure. For the stepwise model with structure correction, additional bond removal is performed for carbon atoms exhibiting large prediction errors (MAE > 10 ppm) and their neighbouring N, O, and F atoms. The stepwise model subsequently reassigns bonds for these atoms to correct and complete the structure.

A multi-shot noise-augmented strategy was applied to inverse-IMPRESSION models trained on NMR Datasets B and C. For each NMR input, 200 structural candidates are generated by adding gaussian noise to the input chemical shifts and scalar coupling constants, with standard deviations of 0.3 ppm for ^{1}H, 2.5 ppm for ^{13}C, and 7.5 ppm for ^{15}N and ^{19}F, and 0.4 Hz for all coupling constants between NMR-active nuclei. After noise augmentation, the resulting edges with coupling constants of over 2 Hz are assigned as the corresponding coupling correlation type (see Supplementary Section 1.2.5 for details), with the remainder assigned as uncorrelated (value of 0). Each noise-perturbed input yields a single predicted structure, producing an ensemble of candidate structures for a given set of NMR parameters. The chemical validity of the generated candidates was assessed using the sanitization function implemented in the RDKit package.

The prediction performance of inverse-IMPRESSION models are evaluated by the following metrics:

- **Single prediction accuracy**: the fraction of tests in which the correct structure is predicted using the original NMR parameters without noise augmentation.
- **Validity**: the fraction of tests where at least one chemically valid structure appears among the 200 generated candidates.
- **Top-200 accuracy**: the fraction of tests where the correct structure appears among the 200 generated candidates prior to ranking, which indicates the upper bound of the ranking's ability to recover the correct structure.
- **Top-10 frequency accuracy**: the fraction of tests where the correct structure appears within the 10 most frequent candidates.
- **Top-3 frequency accuracy**: the fraction of tests where the correct structure appears within the 3 most frequent candidates.
- **Top-1 frequency accuracy**: the fraction of tests where the correct structure appears as the most frequent candidate.

Supplementary Table 2. The performance metrics of inverse-IMPRESSION models trained with different NMR datasets. Prediction time refers to the average computational time required to elucidate a single molecule using one set of noise-augmented NMR inputs.

Model	NMR dataset	Single prediction accuracy	Validity	Top-200 accuracy	Top-10 frequency accuracy	Top-3 frequency accuracy	Top-1 frequency accuracy	Prediction time
One-shot	A	99.4%	-	-	-	-	-	0.18 s
	B	5.3%	6.8%	6.5%	6.5%	6.5%	6.5%	0.18 s
	C	23.4%	33.4%	31.1%	31.1%	31.1%	30.6%	0.18 s
Stepwise	B	49.6%	95.5%	82.8%	82.7%	81.2%	74.2%	0.84 s
	C	59.4%	94.4%	90.0%	90.0%	89.5%	86.2%	0.53 s
Stepwise with structure correction	B	53.4%	99.2%	89.2%	88.7%	86.4%	77.8%	1.66 s
	C	64.4%	99.5%	93.6%	93.3%	91.7%	86.2%	0.95 s

2.2 Edge-level Analysis on One-Shot Models

An edge-level analysis was performed to evaluate the prediction accuracy for each edge type in the one-shot models. To ensure a concise and clear analysis, no noise was applied during prediction, and only one candidate was generated for each molecule. Two metrics are used to quantify the prediction accuracy:

- **False Negative Rate (FNR):** defined as FN/(TP+FN), where FN is the number of false negative edges among one edge type, and TP is the number of true positive edges. The FNR indicates the proportion of true bonds of each bond type that are not identified in ground-truth molecules.
- **False Discovery Rate (FDR):** defined as FP/(TP+FP), where FP is the number of false positive edges among on edge type, and TP is the number of true positive edges. The FDR indicates the proportion of predicted bonds of each bond type that are incorrectly identified, i.e., bonds predicted by the model that do not exist in the true molecule.

Supplementary Table 3. False negative rates (FNR) and false discovery rates (FDR) associated with each edge type from the one-shot models

Edge type	NMR Dataset A		NMR Dataset B		NMR Dataset C	
	FNR (%)	FDR (%)	FNR (%)	FDR (%)	FNR (%)	FDR (%)
**C-C**	0.00	0.00	3.26	4.21	2.59	2.87
**C-H**	0.00	0.00	0.00	0.00	0.00	0.00
**N-H**	0.00	0.00	71.46	35.68	0.00	0.00
**O-H**	0.00	0.00	82.82	40.88	85.88	37.61
**N-C**	0.01	0.01	69.16	34.51	4.45	3.19
**C-O**	0.00	0.00	73.22	34.55	76.16	30.84
**C-F**	0.00	0.00	21.82	17.23	0.00	0.00

2.3 Top-k Accuracy Trends for Inverse-IMPRESSION Models

2.3.1 One-Shot Model B

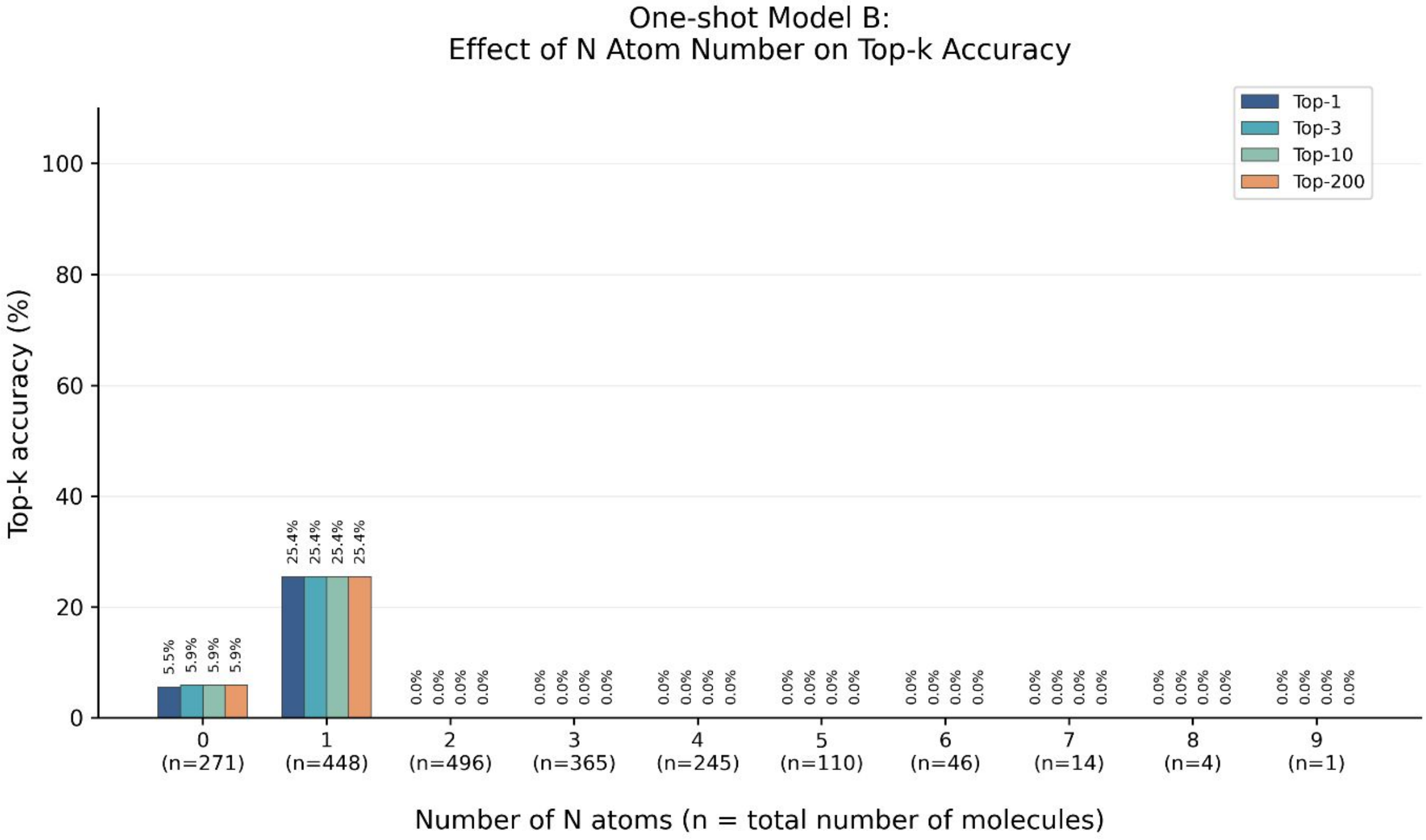

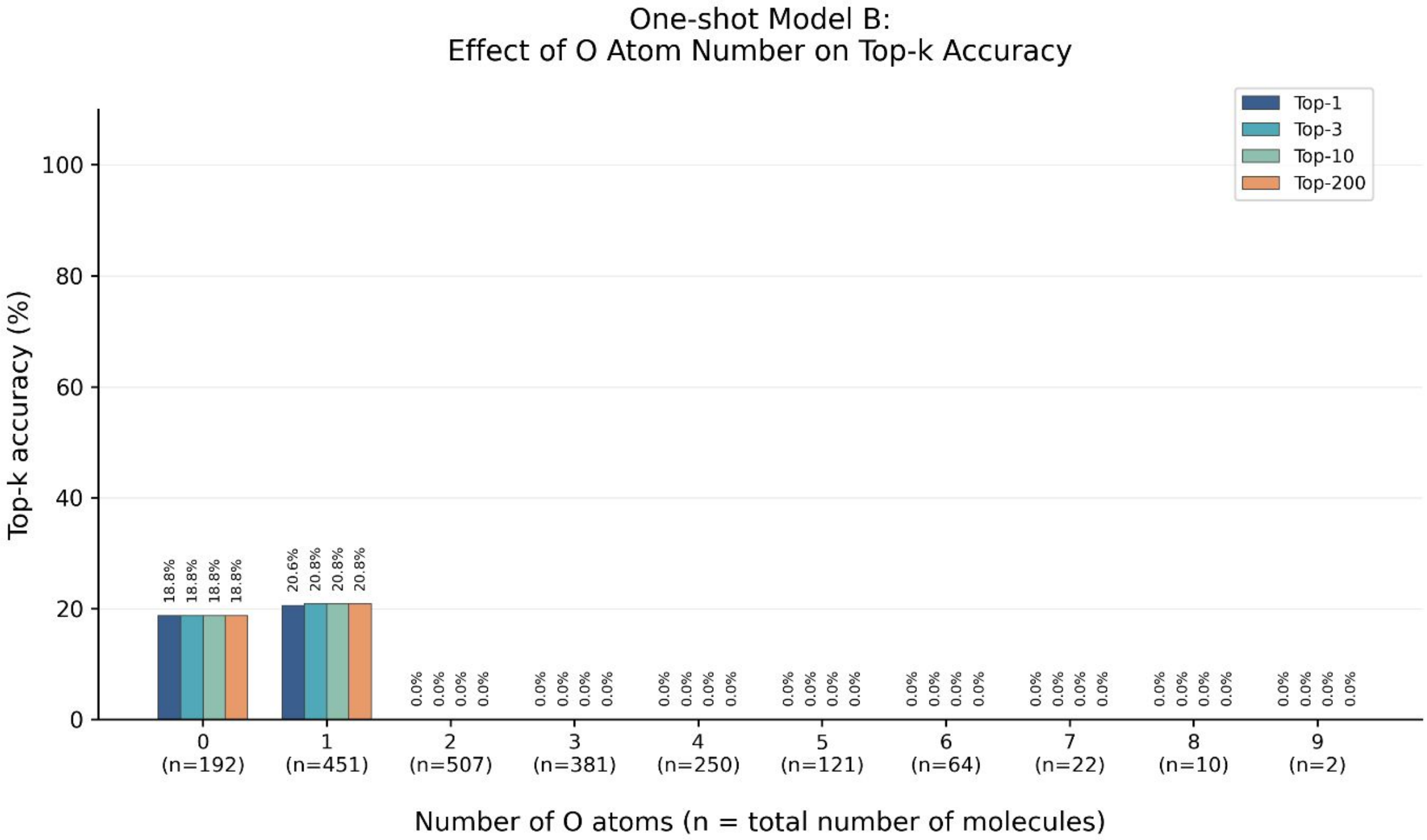

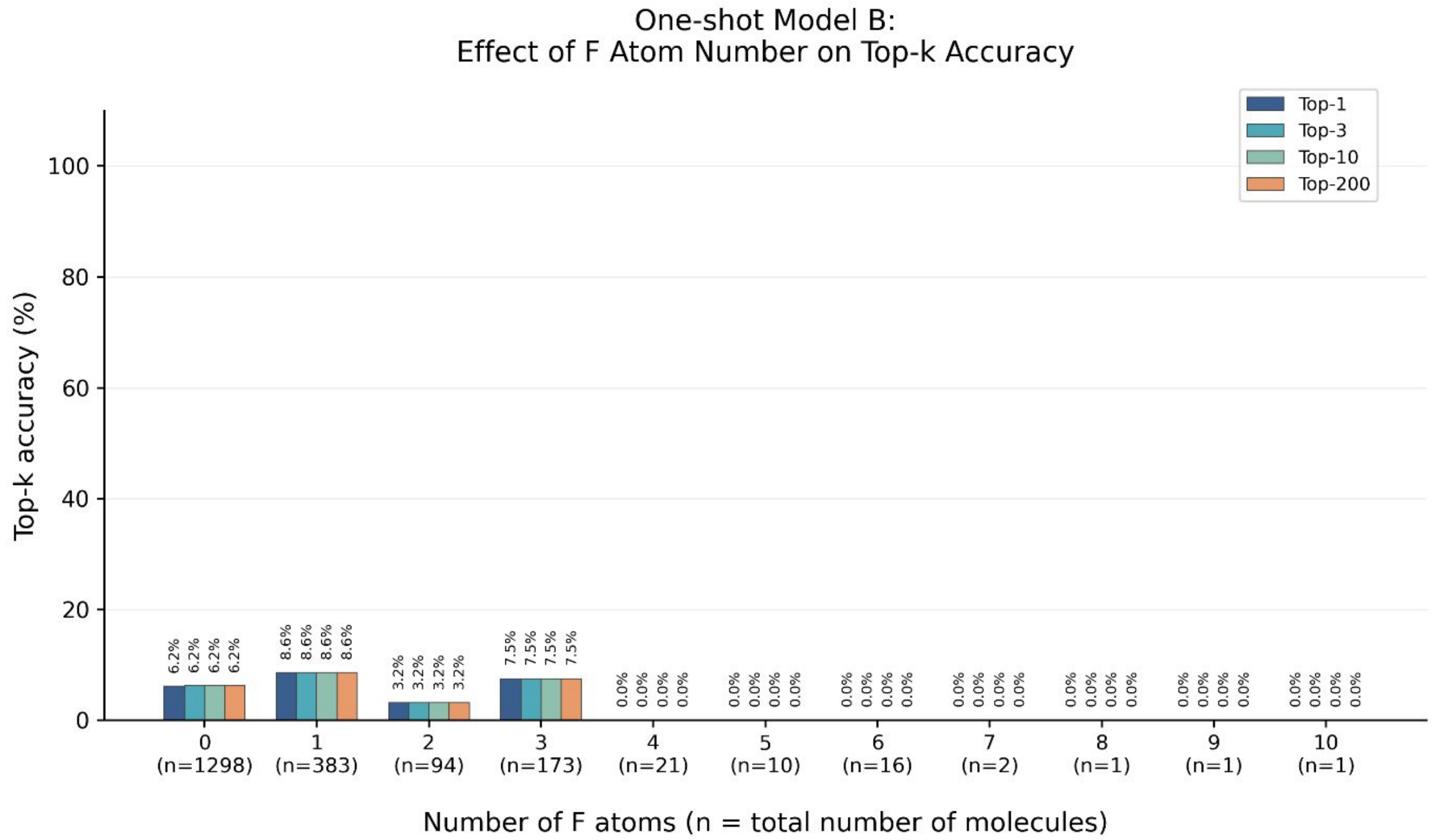
One-shot Model B:
Effect of F Atom Number on Top-k Accuracy
Top-1
Top-3
Top-10
Top-200
Top-k accuracy (%)
0
20
40
60
80
100
6.2%
8.6%
3.2%
7.5%
0.0%
0
(n=1298)
1
(n=383)
2
(n=94)
3
(n=173)
4
(n=21)
5
(n=10)
6
(n=16)
7
(n=2)
8
(n=1)
9
(n=1)
10
(n=1)
Number of F atoms (n = total number of molecules)

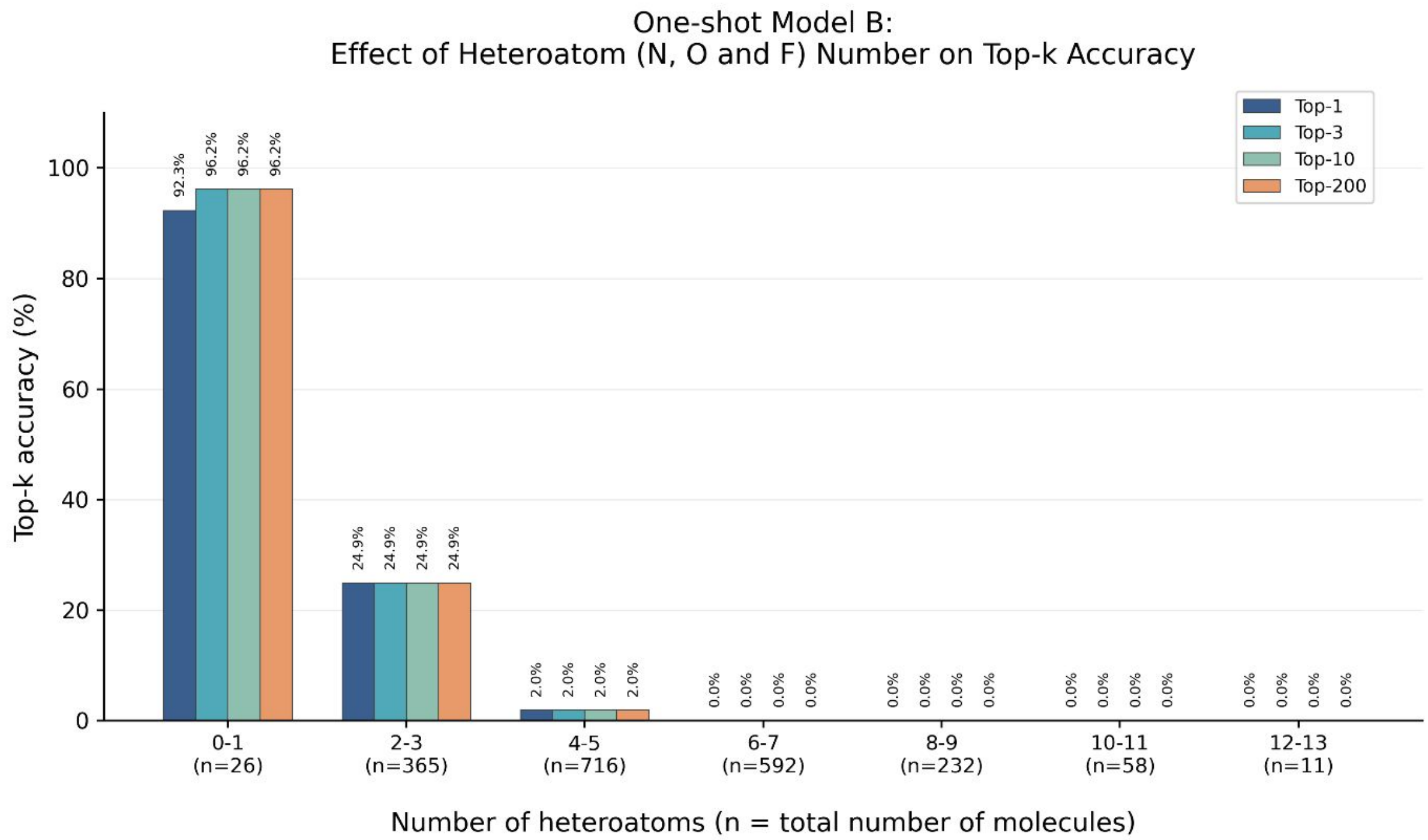
One-shot Model B:
Effect of Heteroatom (N, O and F) Number on Top-k Accuracy
Top-1
Top-3
Top-10
Top-200
Top-k accuracy (%)
0
20
40
60
80
100
92.3%
96.2%
24.9%
2.0%
0.0%
0-1
(n=26)
2-3
(n=365)
4-5
(n=716)
6-7
(n=592)
8-9
(n=232)
10-11
(n=58)
12-13
(n=11)
Number of heteroatoms (n = total number of molecules)

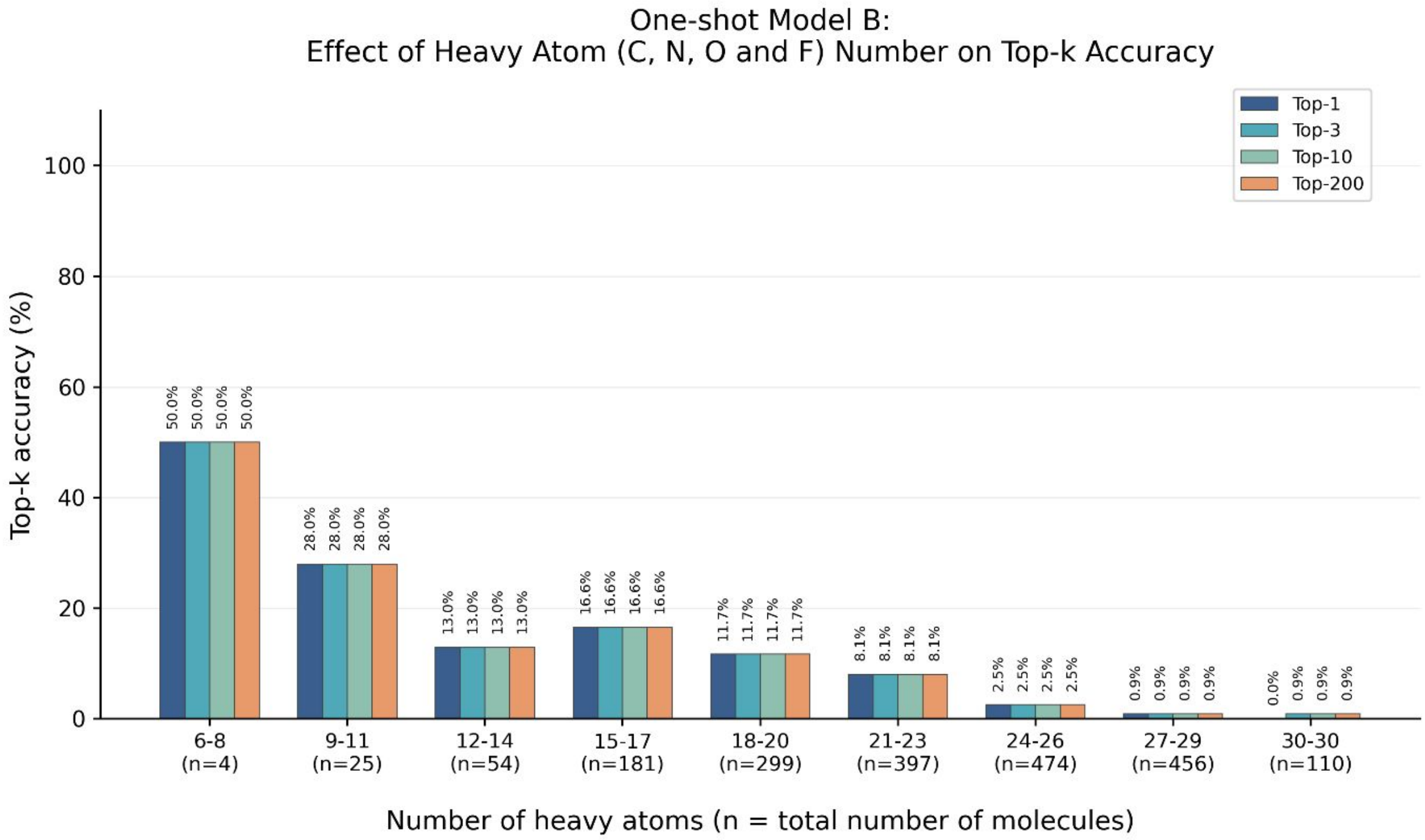
One-shot Model B:
Effect of Heavy Atom (C, N, O and F) Number on Top-k Accuracy
Top-1
Top-3
Top-10
Top-200
Top-k accuracy (%)
0
20
40
60
80
100
50.0% 50.0% 50.0% 50.0%
28.0% 28.0% 28.0% 28.0%
13.0% 13.0% 13.0% 13.0%
16.6% 16.6% 16.6% 16.6%
11.7% 11.7% 11.7% 11.7%
8.1% 8.1% 8.1% 8.1%
2.5% 2.5% 2.5% 2.5%
0.9% 0.9% 0.9% 0.9%
0.0% 0.9% 0.9% 0.9%
6-8 (n=4)
9-11 (n=25)
12-14 (n=54)
15-17 (n=181)
18-20 (n=299)
21-23 (n=397)
24-26 (n=474)
27-29 (n=456)
30-30 (n=110)
Number of heavy atoms (n = total number of molecules)

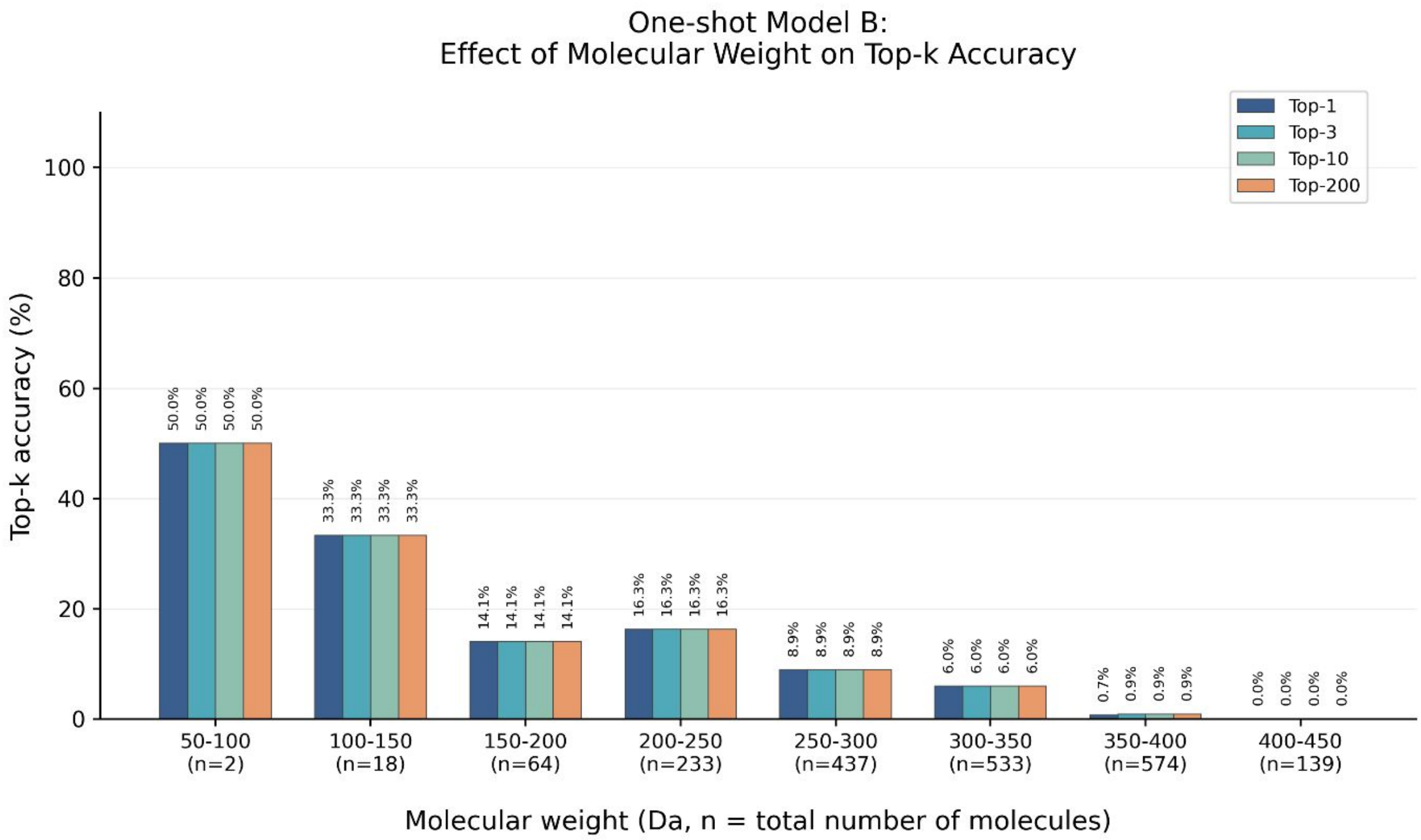
One-shot Model B:
Effect of Molecular Weight on Top-k Accuracy
Top-1
Top-3
Top-10
Top-200
Top-k accuracy (%)
0
20
40
60
80
100
50.0% 50.0% 50.0% 50.0%
33.3% 33.3% 33.3% 33.3%
14.1% 14.1% 14.1% 14.1%
16.3% 16.3% 16.3% 16.3%
8.9% 8.9% 8.9% 8.9%
6.0% 6.0% 6.0% 6.0%
0.7% 0.9% 0.9% 0.9%
0.0% 0.0% 0.0% 0.0%
50-100 (n=2)
100-150 (n=18)
150-200 (n=64)
200-250 (n=233)
250-300 (n=437)
300-350 (n=533)
350-400 (n=574)
400-450 (n=139)
Molecular weight (Da, n = total number of molecules)

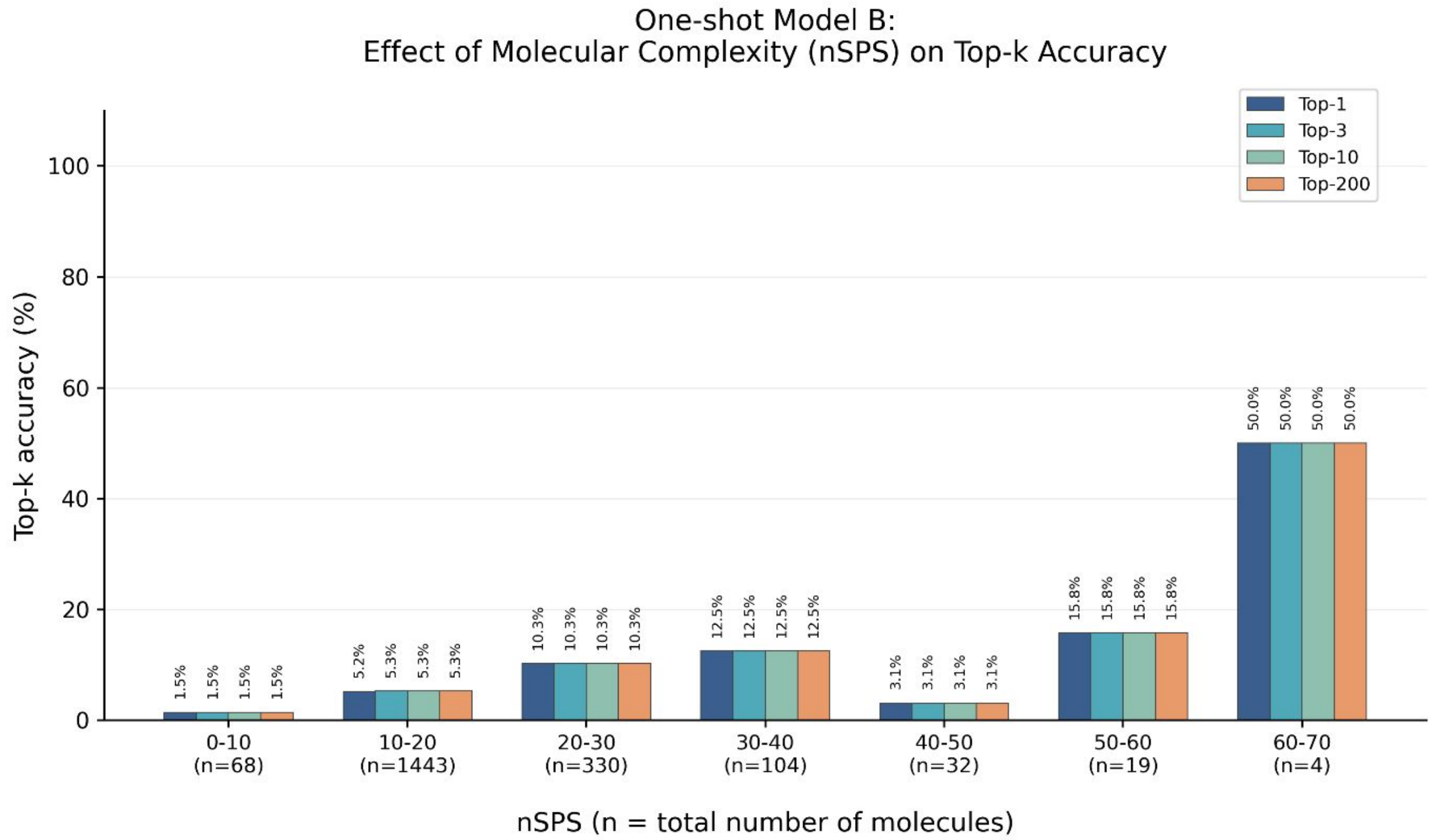
One-shot Model B:
Effect of Molecular Complexity (nSPS) on Top-k Accuracy
Top-1
Top-3
Top-10
Top-200
Top-k accuracy (%)
0
20
40
60
80
100
1.5%
5.2%
5.3%
10.3%
12.5%
3.1%
15.8%
50.0%
0-10
(n=68)
10-20
(n=1443)
20-30
(n=330)
30-40
(n=104)
40-50
(n=32)
50-60
(n=19)
60-70
(n=4)
nSPS (n = total number of molecules)

2.3.2 One-Shot Model C

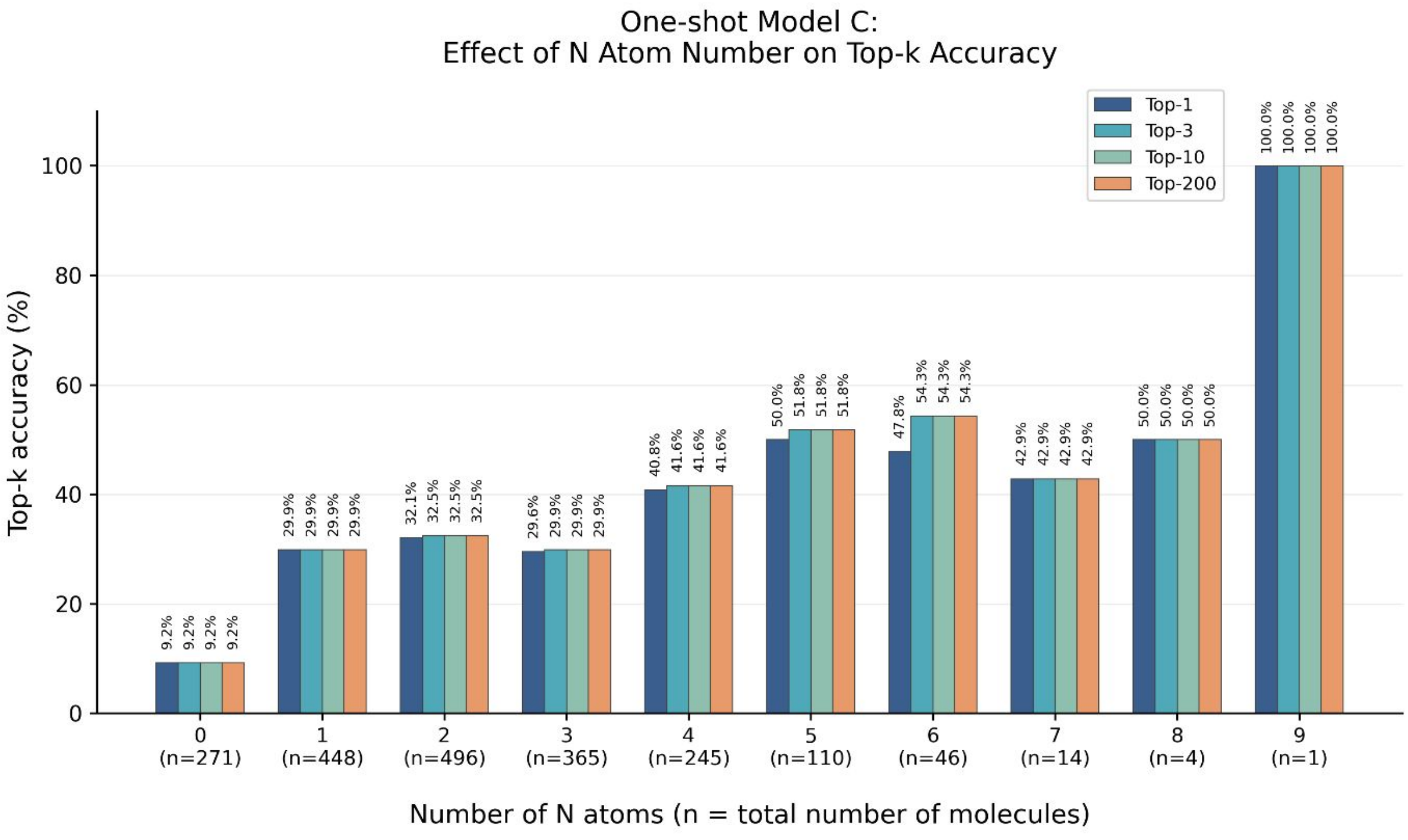

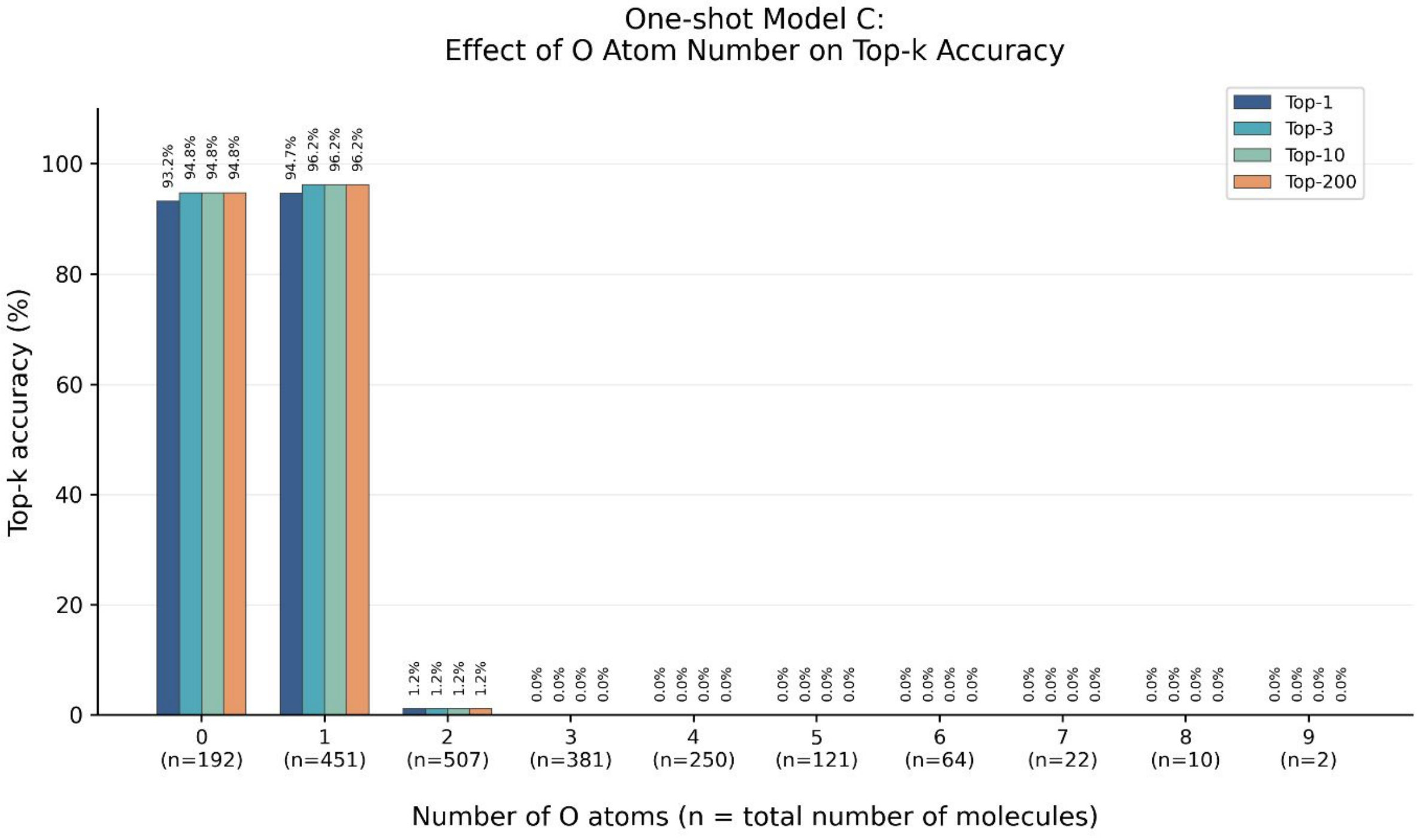

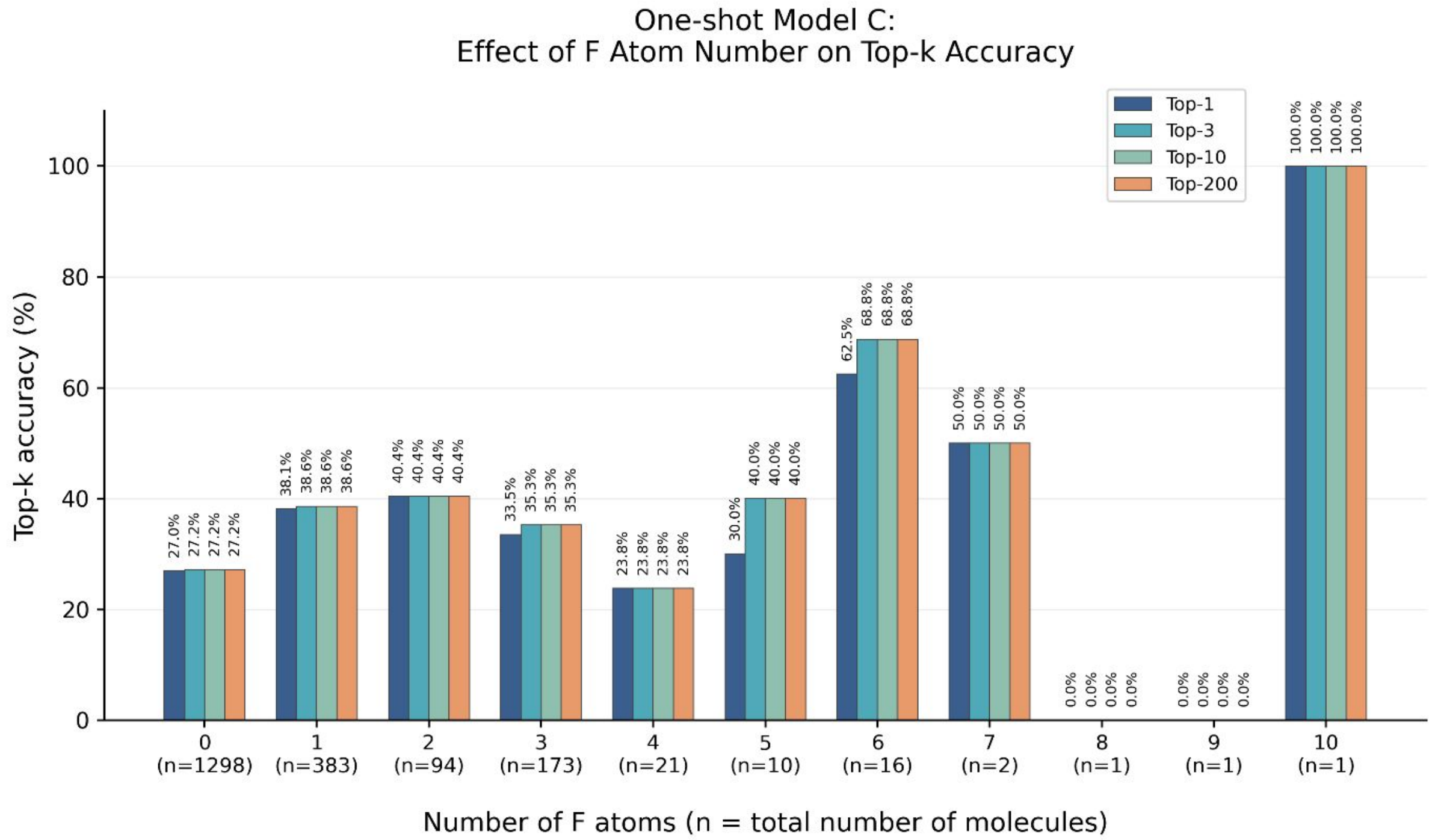
One-shot Model C:
Effect of F Atom Number on Top-k Accuracy
Top-1
Top-3
Top-10
Top-200
Top-k accuracy (%)
0 (n=1298)
1 (n=383)
2 (n=94)
3 (n=173)
4 (n=21)
5 (n=10)
6 (n=16)
7 (n=2)
8 (n=1)
9 (n=1)
10 (n=1)
Number of F atoms (n = total number of molecules)

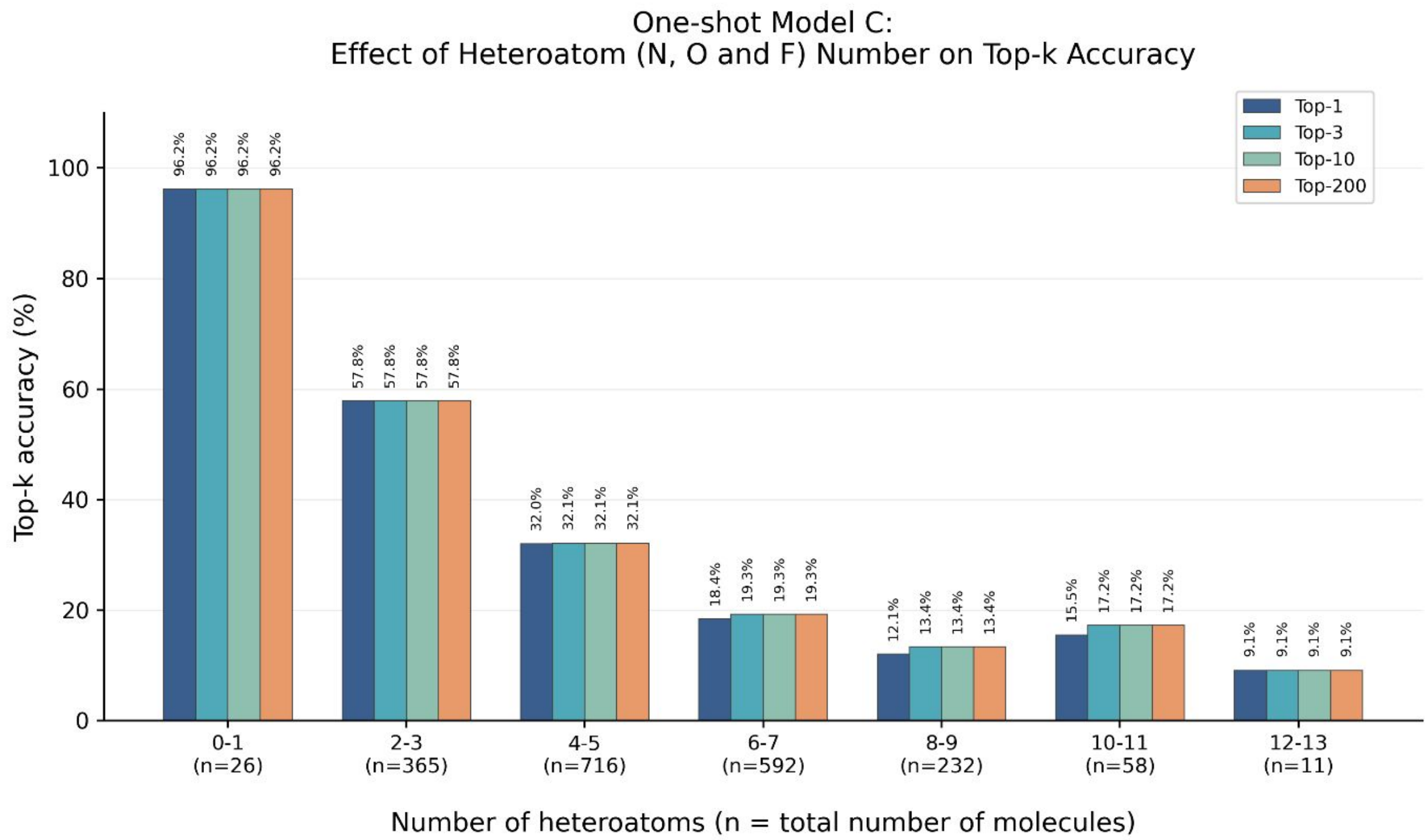
One-shot Model C:
Effect of Heteroatom (N, O and F) Number on Top-k Accuracy
Top-1
Top-3
Top-10
Top-200
Top-k accuracy (%)
0-1 (n=26)
2-3 (n=365)
4-5 (n=716)
6-7 (n=592)
8-9 (n=232)
10-11 (n=58)
12-13 (n=11)
Number of heteroatoms (n = total number of molecules)

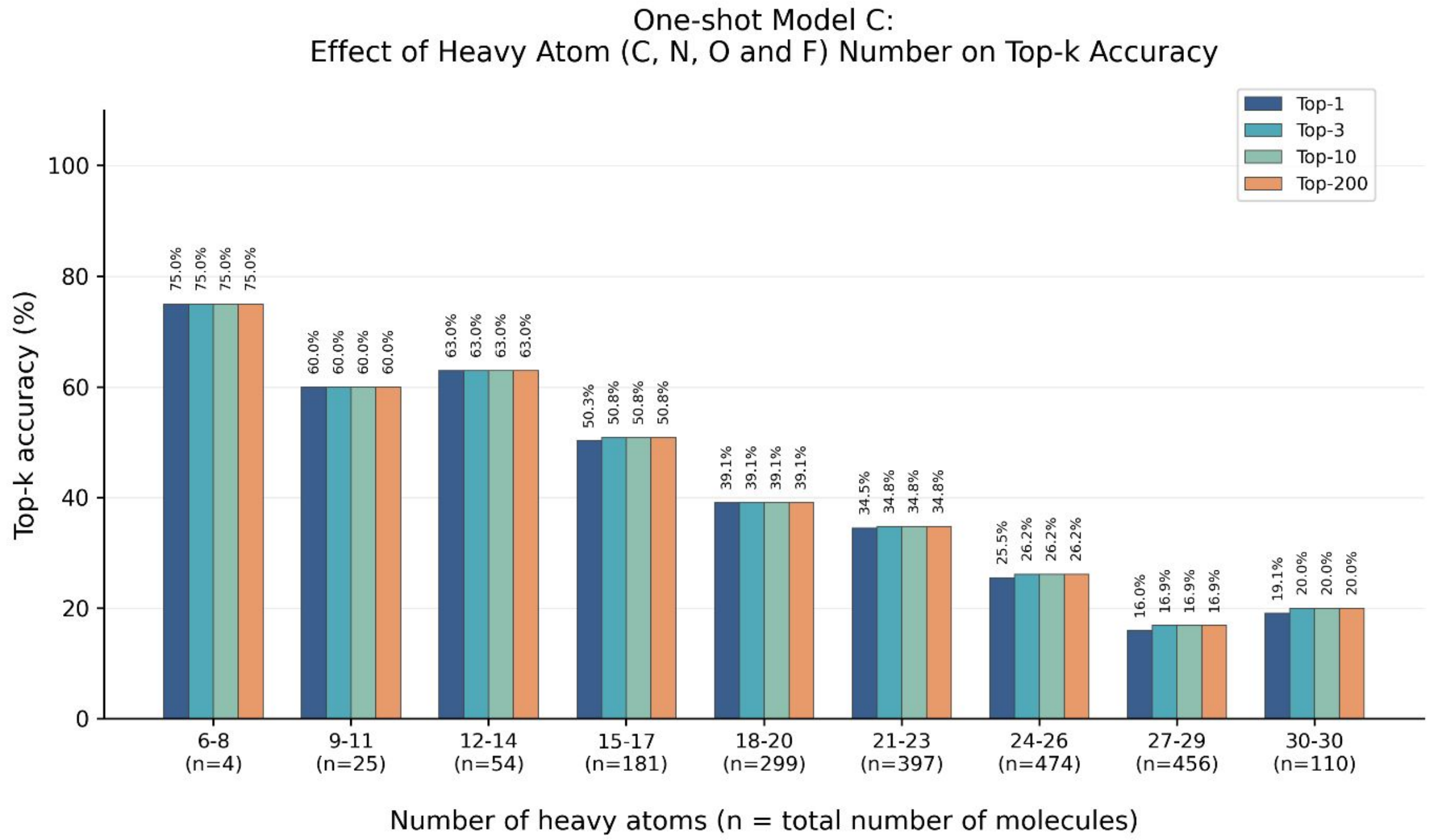
One-shot Model C:
Effect of Heavy Atom (C, N, O and F) Number on Top-k Accuracy
Top-1
Top-3
Top-10
Top-200
Top-k accuracy (%)
0
20
40
60
80
100
6-8 (n=4)
9-11 (n=25)
12-14 (n=54)
15-17 (n=181)
18-20 (n=299)
21-23 (n=397)
24-26 (n=474)
27-29 (n=456)
30-30 (n=110)
75.0% 75.0% 75.0% 75.0%
60.0% 60.0% 60.0% 60.0%
63.0% 63.0% 63.0% 63.0%
50.3% 50.8% 50.8% 50.8%
39.1% 39.1% 39.1% 39.1%
34.5% 34.8% 34.8% 34.8%
25.5% 26.2% 26.2% 26.2%
16.0% 16.9% 16.9% 16.9%
19.1% 20.0% 20.0% 20.0%
Number of heavy atoms (n = total number of molecules)

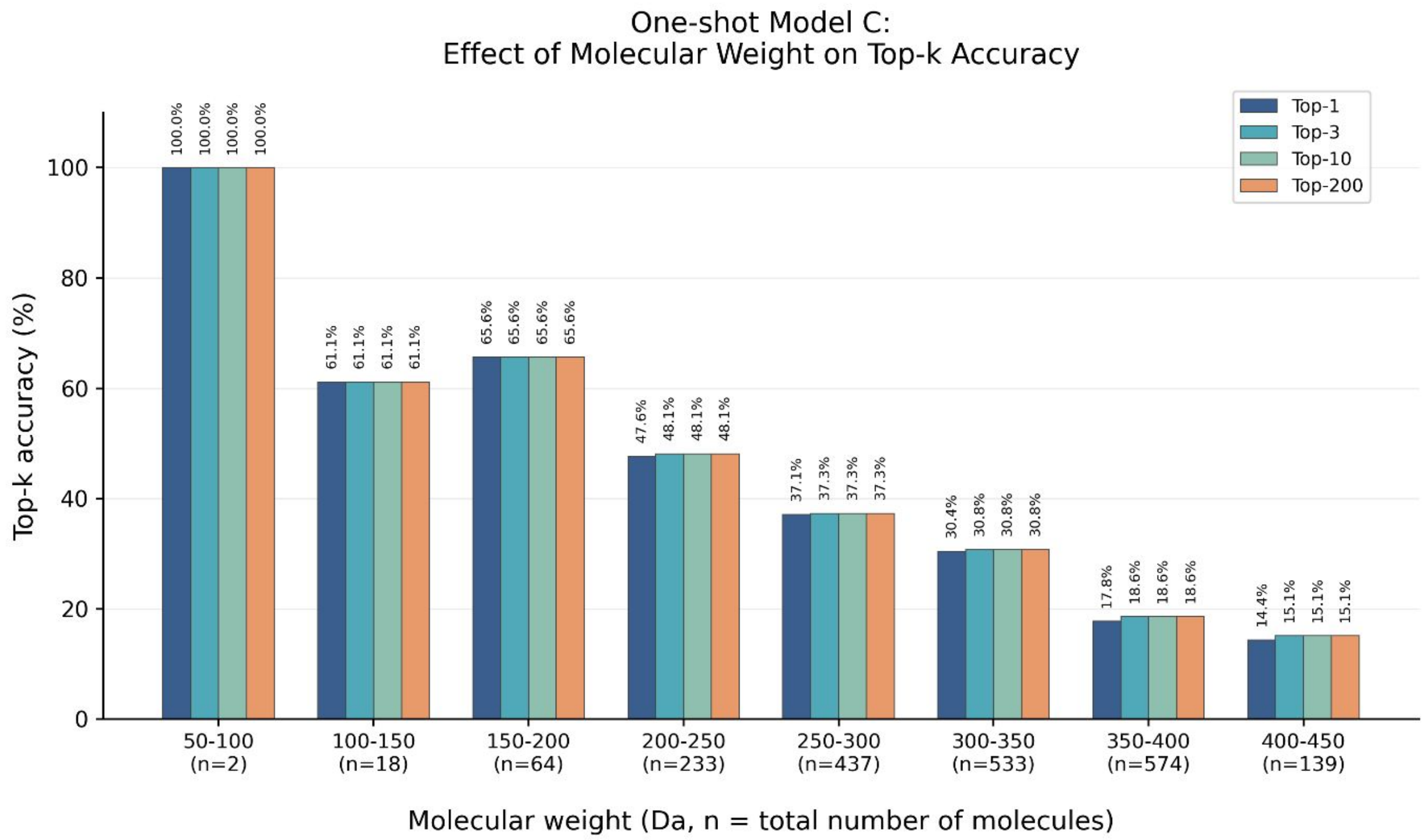
One-shot Model C:
Effect of Molecular Weight on Top-k Accuracy
Top-1
Top-3
Top-10
Top-200
Top-k accuracy (%)
0
20
40
60
80
100
50-100 (n=2)
100-150 (n=18)
150-200 (n=64)
200-250 (n=233)
250-300 (n=437)
300-350 (n=533)
350-400 (n=574)
400-450 (n=139)
100.0% 100.0% 100.0% 100.0%
61.1% 61.1% 61.1% 61.1%
65.6% 65.6% 65.6% 65.6%
47.6% 48.1% 48.1% 48.1%
37.1% 37.3% 37.3% 37.3%
30.4% 30.8% 30.8% 30.8%
17.8% 18.6% 18.6% 18.6%
14.4% 15.1% 15.1% 15.1%
Molecular weight (Da, n = total number of molecules)

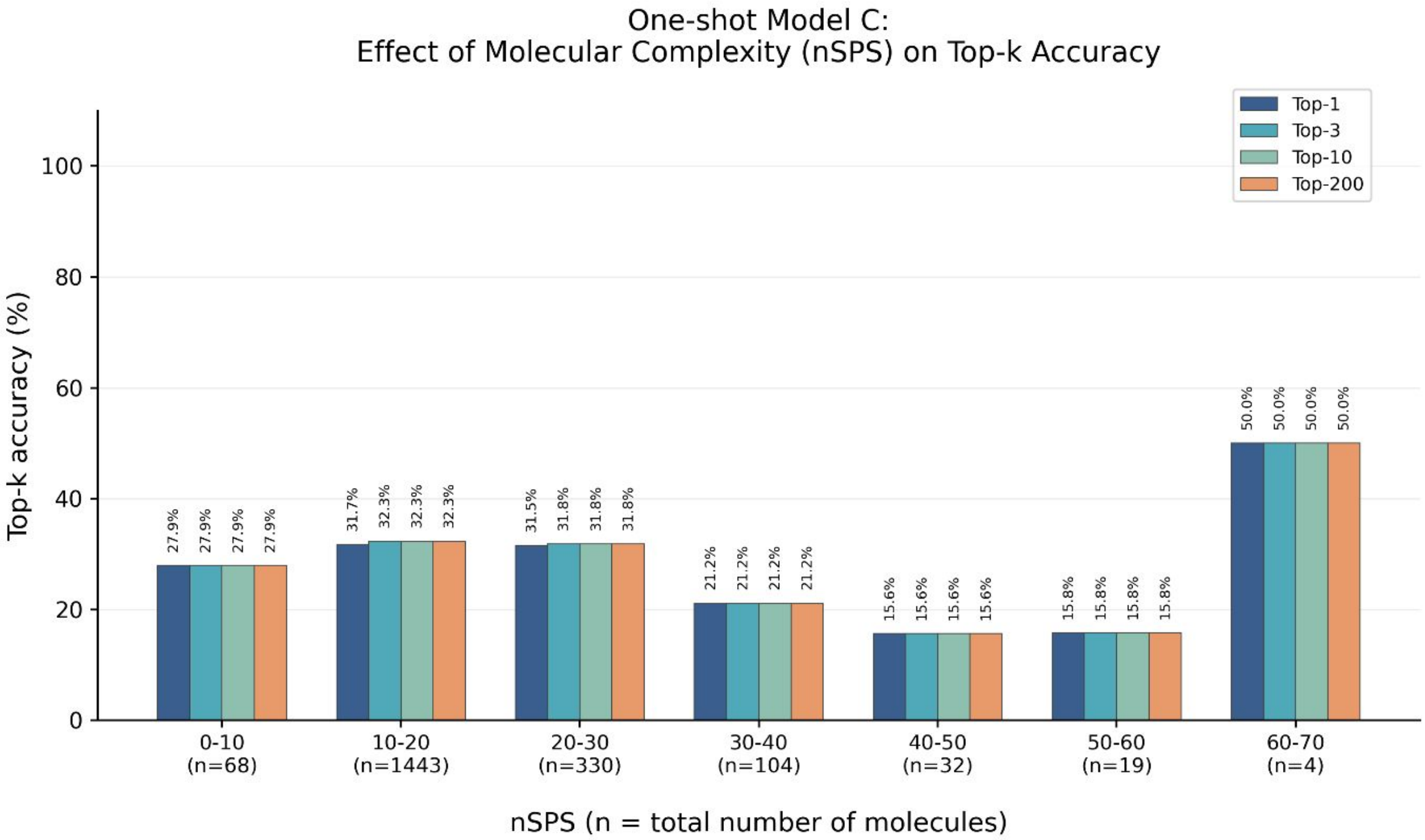
One-shot Model C:
Effect of Molecular Complexity (nSPS) on Top-k Accuracy
Top-1
Top-3
Top-10
Top-200
Top-k accuracy (%)
0
20
40
60
80
100
27.9%
27.9%
27.9%
27.9%
31.7%
32.3%
32.3%
32.3%
31.5%
31.8%
31.8%
31.8%
21.2%
21.2%
21.2%
21.2%
15.6%
15.6%
15.6%
15.6%
15.8%
15.8%
15.8%
15.8%
50.0%
50.0%
50.0%
50.0%
0-10 (n=68)
10-20 (n=1443)
20-30 (n=330)
30-40 (n=104)
40-50 (n=32)
50-60 (n=19)
60-70 (n=4)
nSPS (n = total number of molecules)

2.3.3 Stepwise Model B With Structure Correction

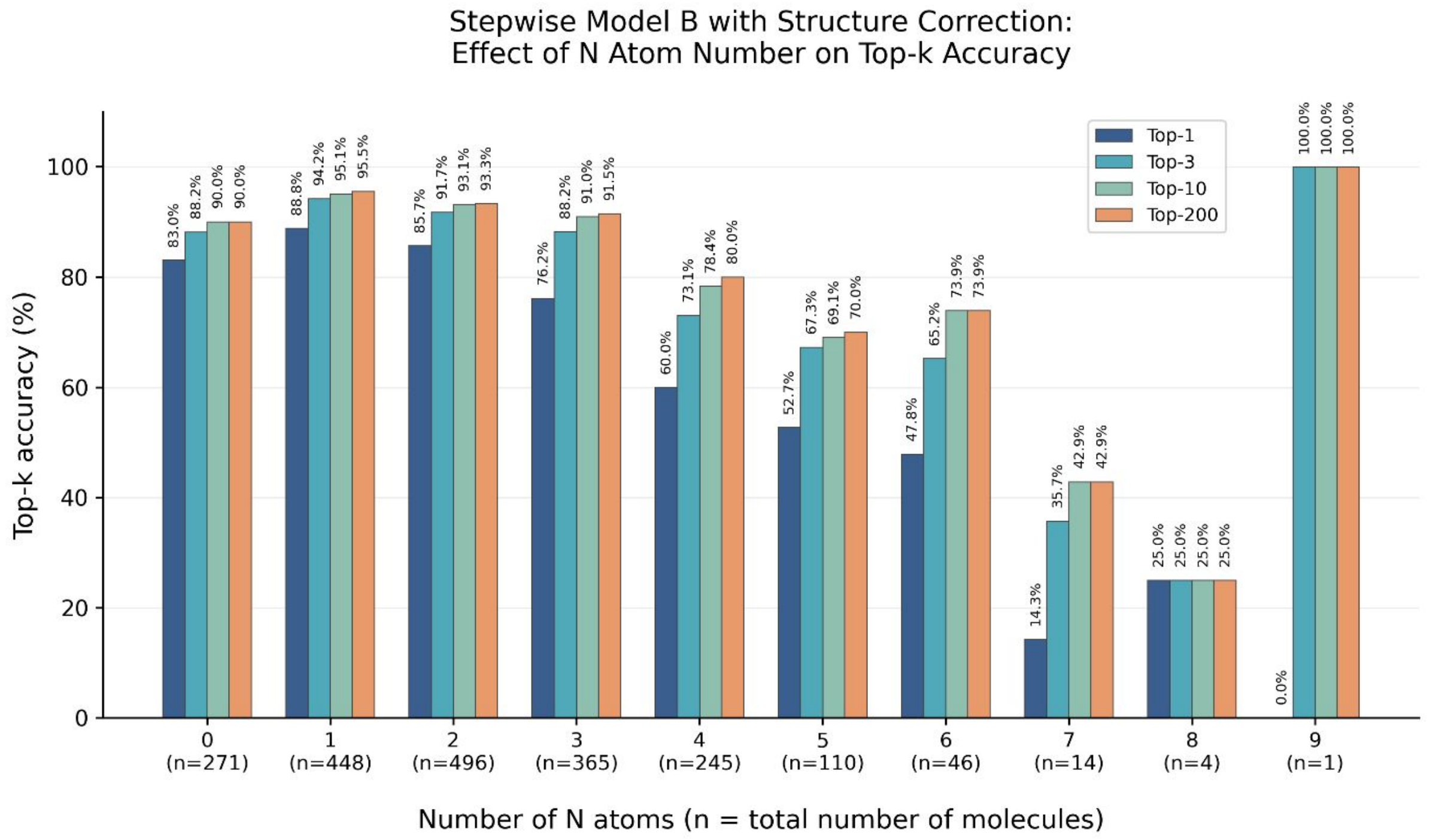

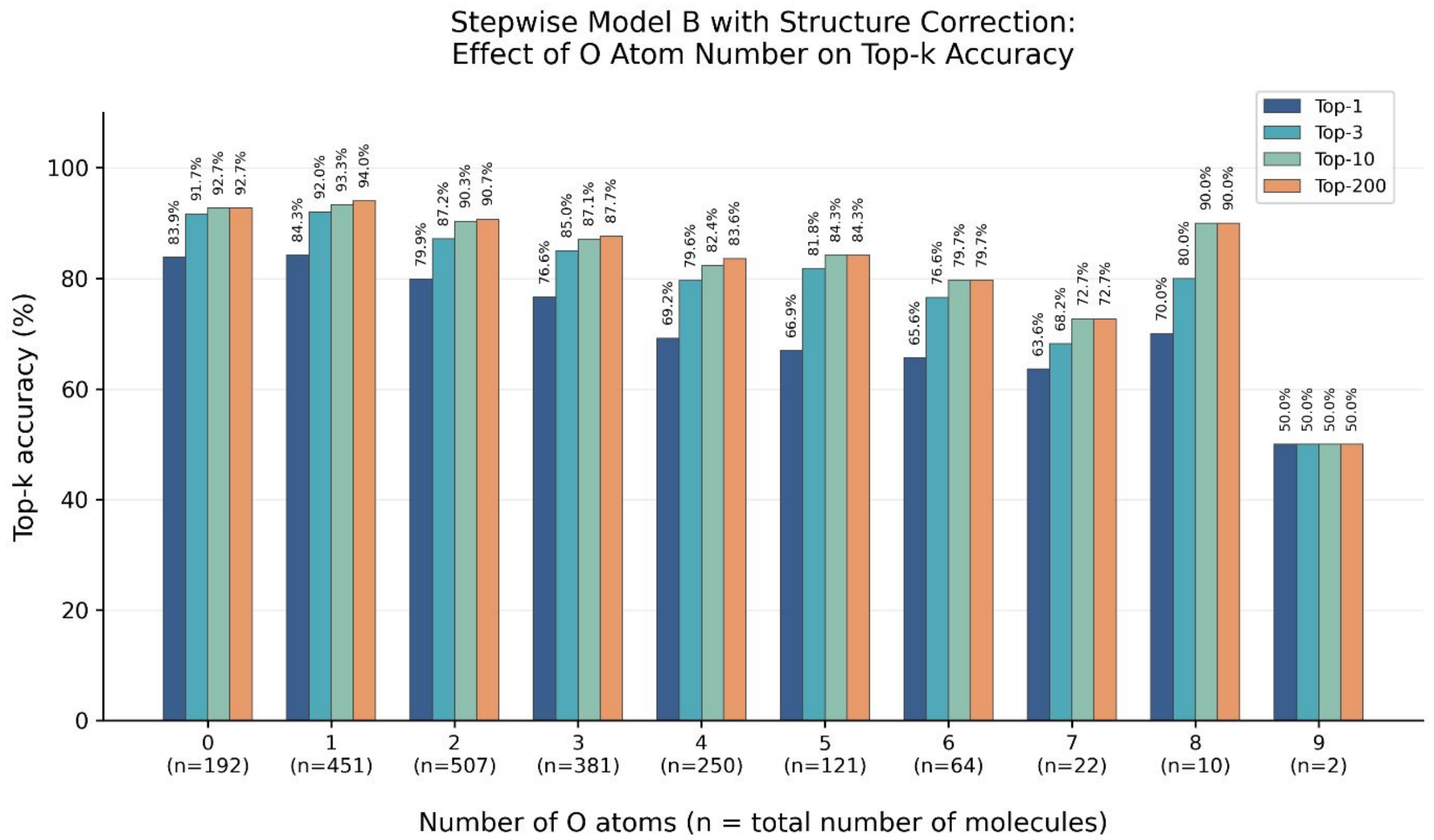

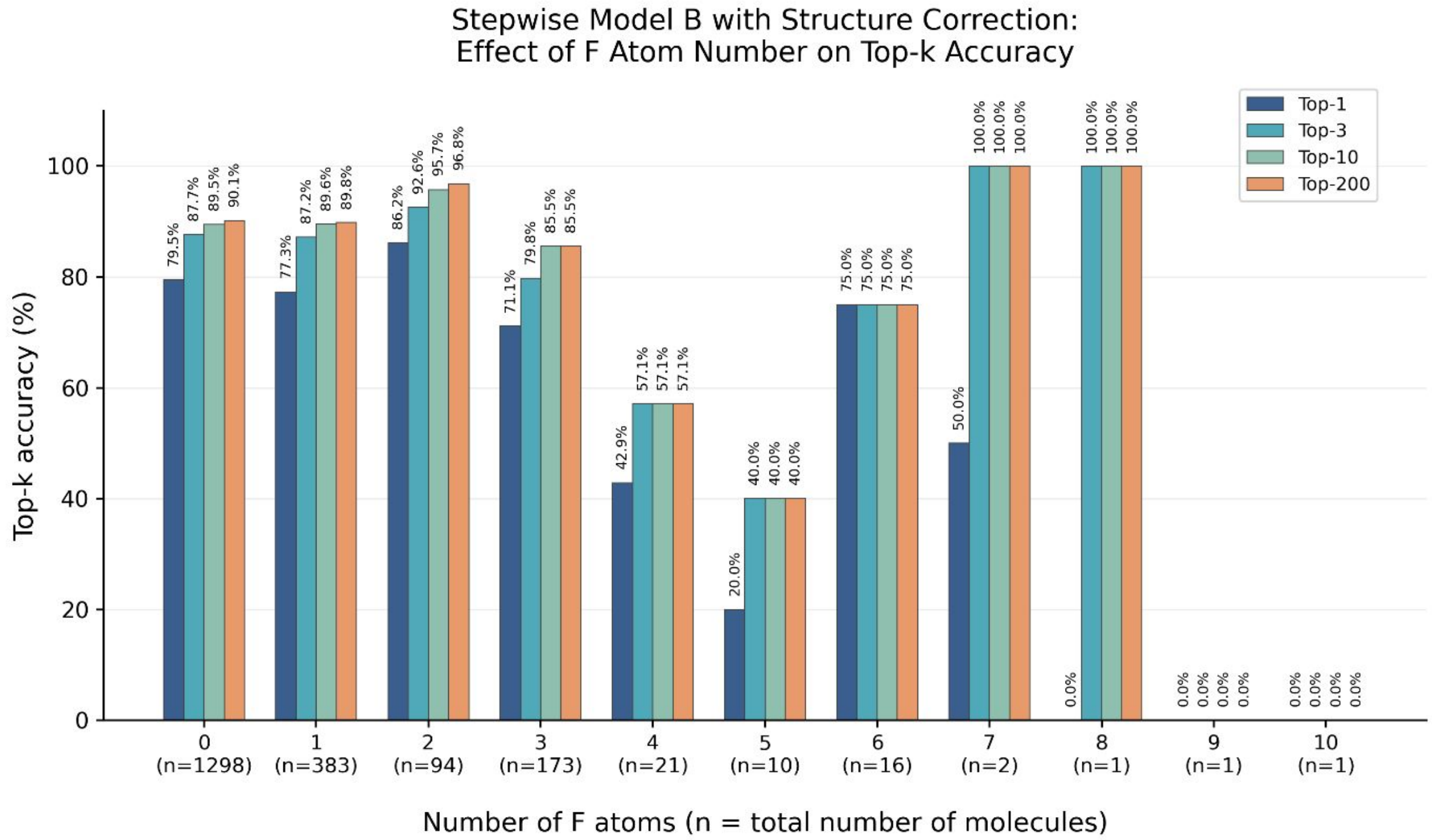

Stepwise Model B with Structure Correction:
Effect of F Atom Number on Top-k Accuracy
Top-1
Top-3
Top-10
Top-200
Top-k accuracy (%)
Number of F atoms (n = total number of molecules)
0 (n=1298)
1 (n=383)
2 (n=94)
3 (n=173)
4 (n=21)
5 (n=10)
6 (n=16)
7 (n=2)
8 (n=1)
9 (n=1)
10 (n=1)
79.5% 87.7% 89.5% 90.1%
77.3% 87.2% 89.6% 89.8%
86.2% 92.6% 95.7% 96.8%
71.1% 79.8% 85.5% 85.5%
42.9% 57.1% 57.1% 57.1%
20.0% 40.0% 40.0% 40.0%
75.0% 75.0% 75.0% 75.0%
50.0% 100.0% 100.0% 100.0%
0.0% 100.0% 100.0% 100.0%
0.0% 0.0% 0.0% 0.0%
0.0% 0.0% 0.0% 0.0%

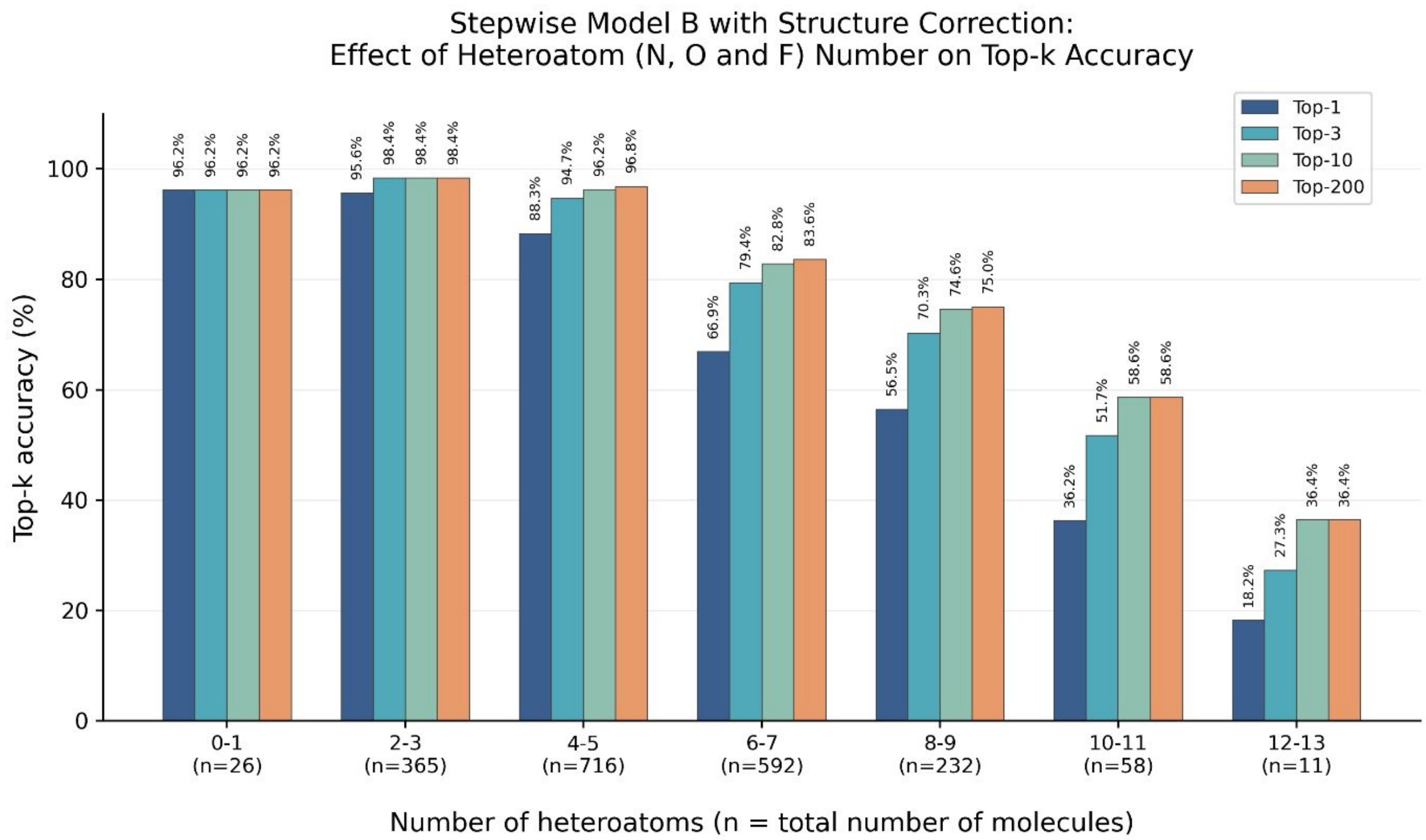

Stepwise Model B with Structure Correction:
Effect of Heteroatom (N, O and F) Number on Top-k Accuracy
Top-1
Top-3
Top-10
Top-200
Top-k accuracy (%)
Number of heteroatoms (n = total number of molecules)
0-1 (n=26)
2-3 (n=365)
4-5 (n=716)
6-7 (n=592)
8-9 (n=232)
10-11 (n=58)
12-13 (n=11)
96.2% 96.2% 96.2% 96.2%
95.6% 98.4% 98.4% 98.4%
88.3% 94.7% 96.2% 96.8%
66.9% 79.4% 82.8% 83.6%
56.5% 70.3% 74.6% 75.0%
36.2% 51.7% 58.6% 58.6%
18.2% 27.3% 36.4% 36.4%

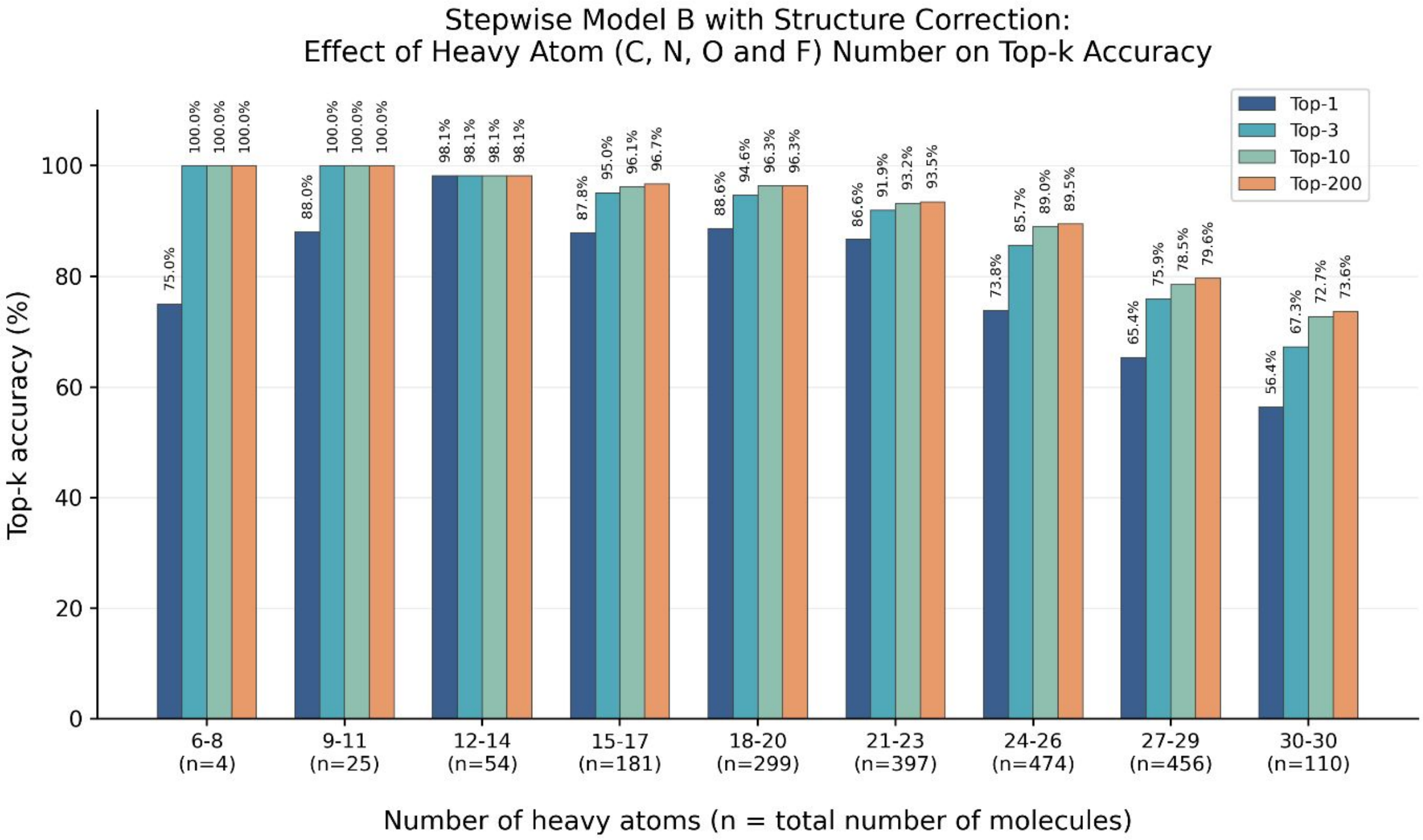
Stepwise Model B with Structure Correction:
Effect of Heavy Atom (C, N, O and F) Number on Top-k Accuracy
Top-k accuracy (%)
0
20
40
60
80
100
Top-1
Top-3
Top-10
Top-200
75.0% 100.0% 100.0% 100.0%
88.0% 100.0% 100.0% 100.0%
98.1% 98.1% 98.1% 98.1%
87.8% 95.0% 96.1% 96.7%
88.6% 94.6% 96.3% 96.3%
86.6% 91.9% 93.2% 93.5%
73.8% 85.7% 89.0% 89.5%
65.4% 75.9% 78.5% 79.6%
56.4% 67.3% 72.7% 73.6%
6-8 (n=4)
9-11 (n=25)
12-14 (n=54)
15-17 (n=181)
18-20 (n=299)
21-23 (n=397)
24-26 (n=474)
27-29 (n=456)
30-30 (n=110)
Number of heavy atoms (n = total number of molecules)

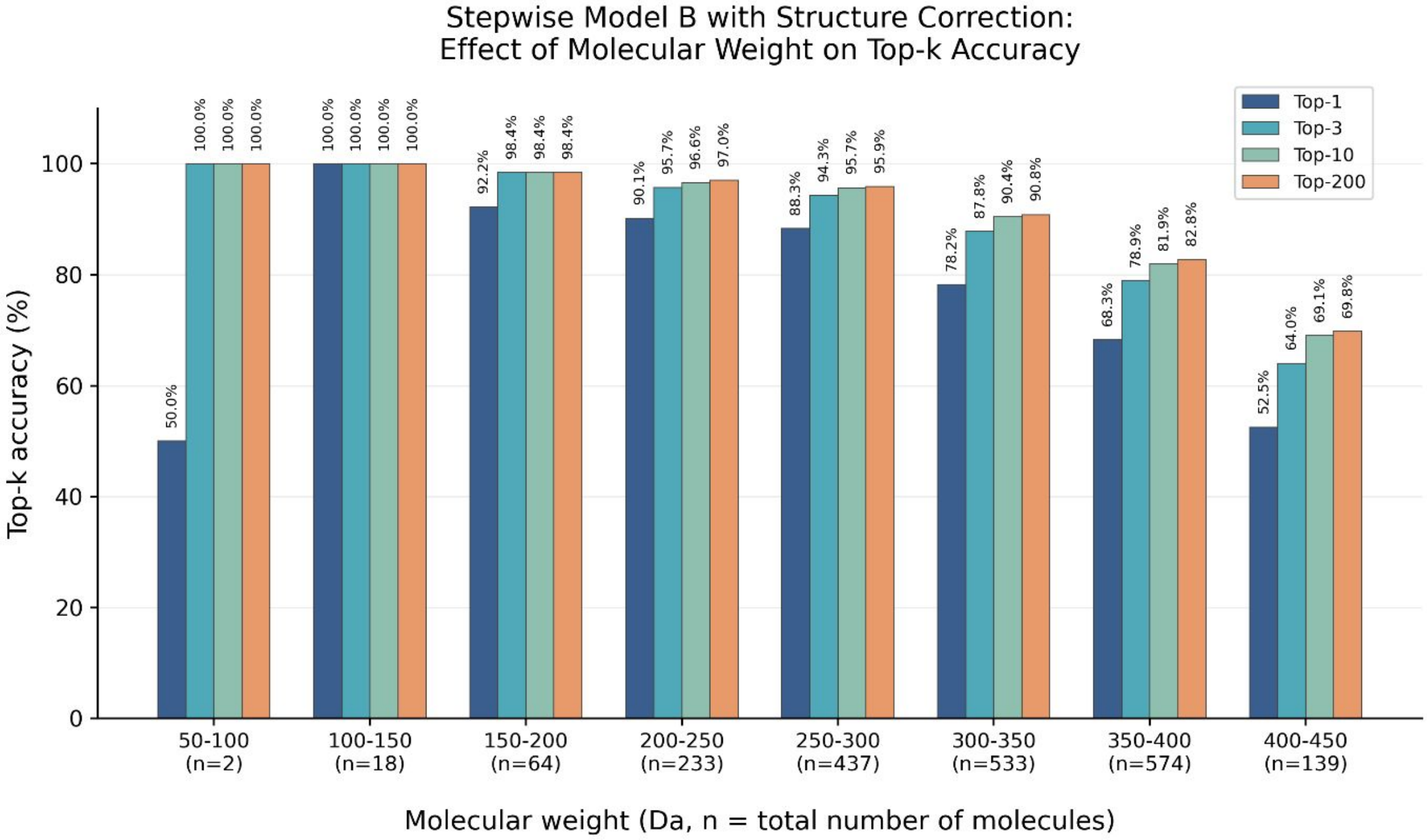
Stepwise Model B with Structure Correction:
Effect of Molecular Weight on Top-k Accuracy
Top-k accuracy (%)
0
20
40
60
80
100
Top-1
Top-3
Top-10
Top-200
50.0% 100.0% 100.0% 100.0%
100.0% 100.0% 100.0% 100.0%
92.2% 98.4% 98.4% 98.4%
90.1% 95.7% 96.6% 97.0%
88.3% 94.3% 95.7% 95.9%
78.2% 87.8% 90.4% 90.8%
68.3% 78.9% 81.9% 82.8%
52.5% 64.0% 69.1% 69.8%
50-100 (n=2)
100-150 (n=18)
150-200 (n=64)
200-250 (n=233)
250-300 (n=437)
300-350 (n=533)
350-400 (n=574)
400-450 (n=139)
Molecular weight (Da, n = total number of molecules)

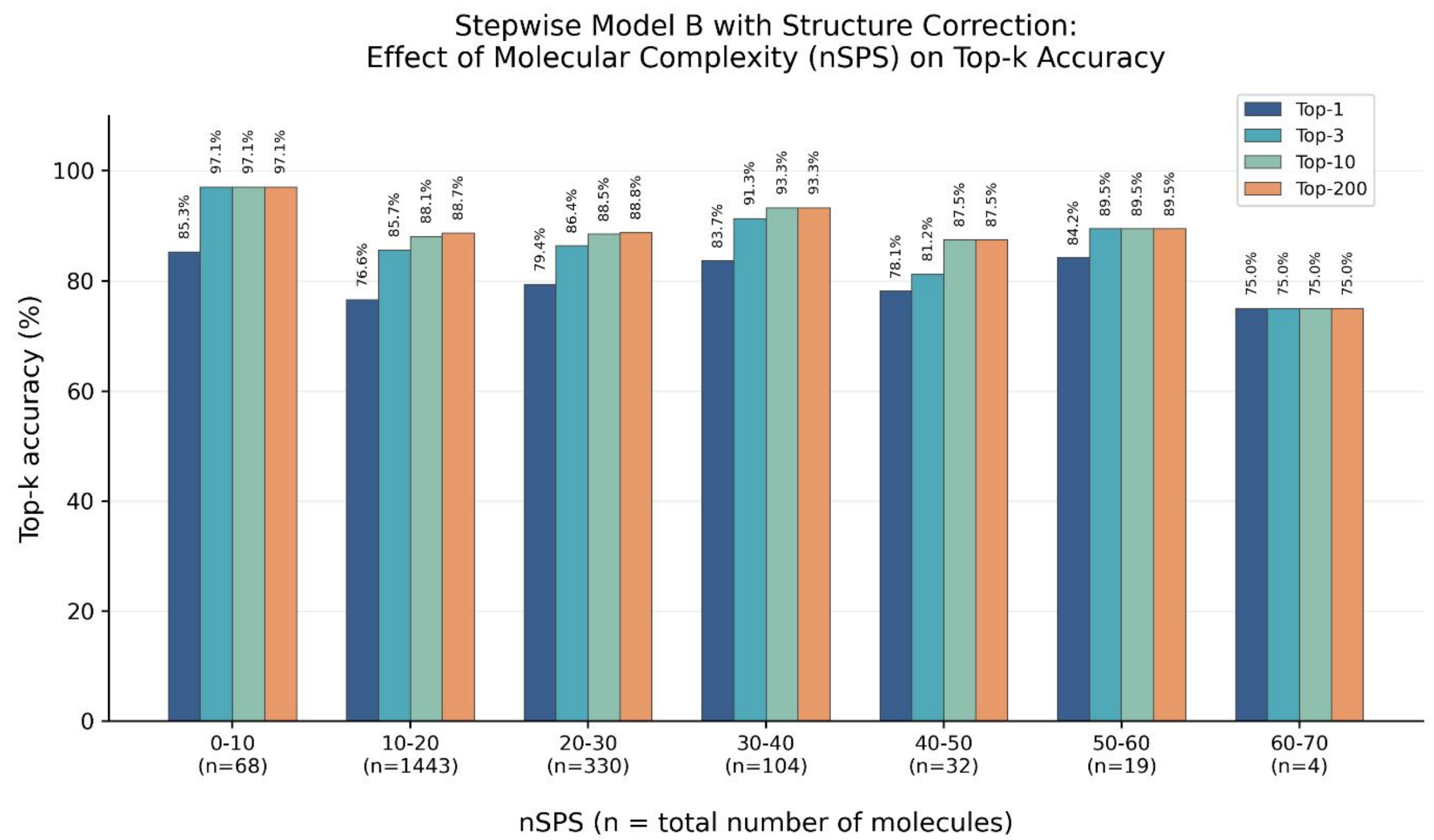

Stepwise Model B with Structure Correction:
Effect of Molecular Complexity (nSPS) on Top-k Accuracy
Top-1
Top-3
Top-10
Top-200
Top-k accuracy (%)
0
20
40
60
80
100
85.3%
97.1%
97.1%
97.1%
76.6%
85.7%
88.1%
88.7%
79.4%
86.4%
88.5%
88.8%
83.7%
91.3%
93.3%
93.3%
78.1%
81.2%
87.5%
87.5%
84.2%
89.5%
89.5%
89.5%
75.0%
75.0%
75.0%
75.0%
0-10 (n=68)
10-20 (n=1443)
20-30 (n=330)
30-40 (n=104)
40-50 (n=32)
50-60 (n=19)
60-70 (n=4)
nSPS (n = total number of molecules)

2.3.4 Stepwise Model C With Structure Correction

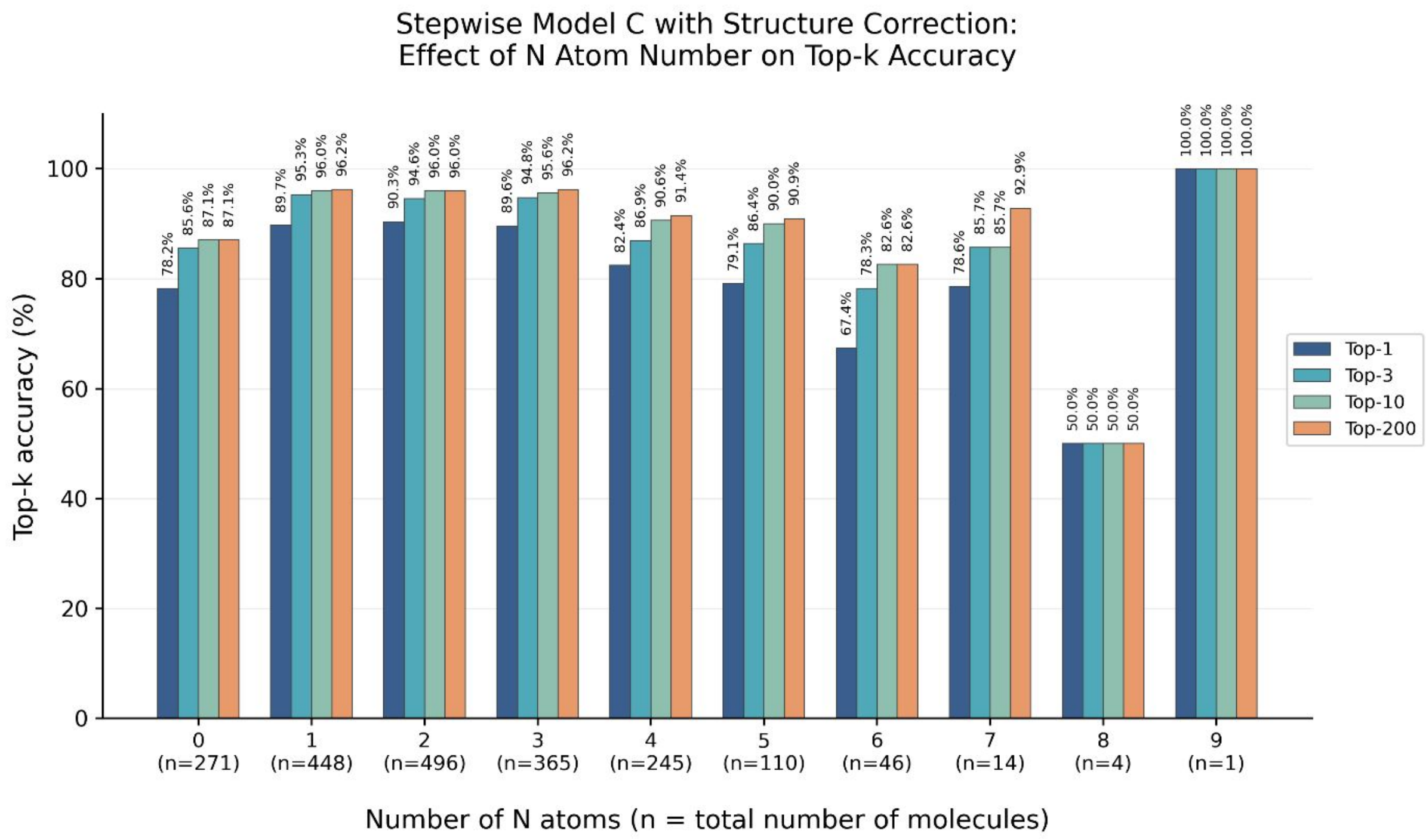

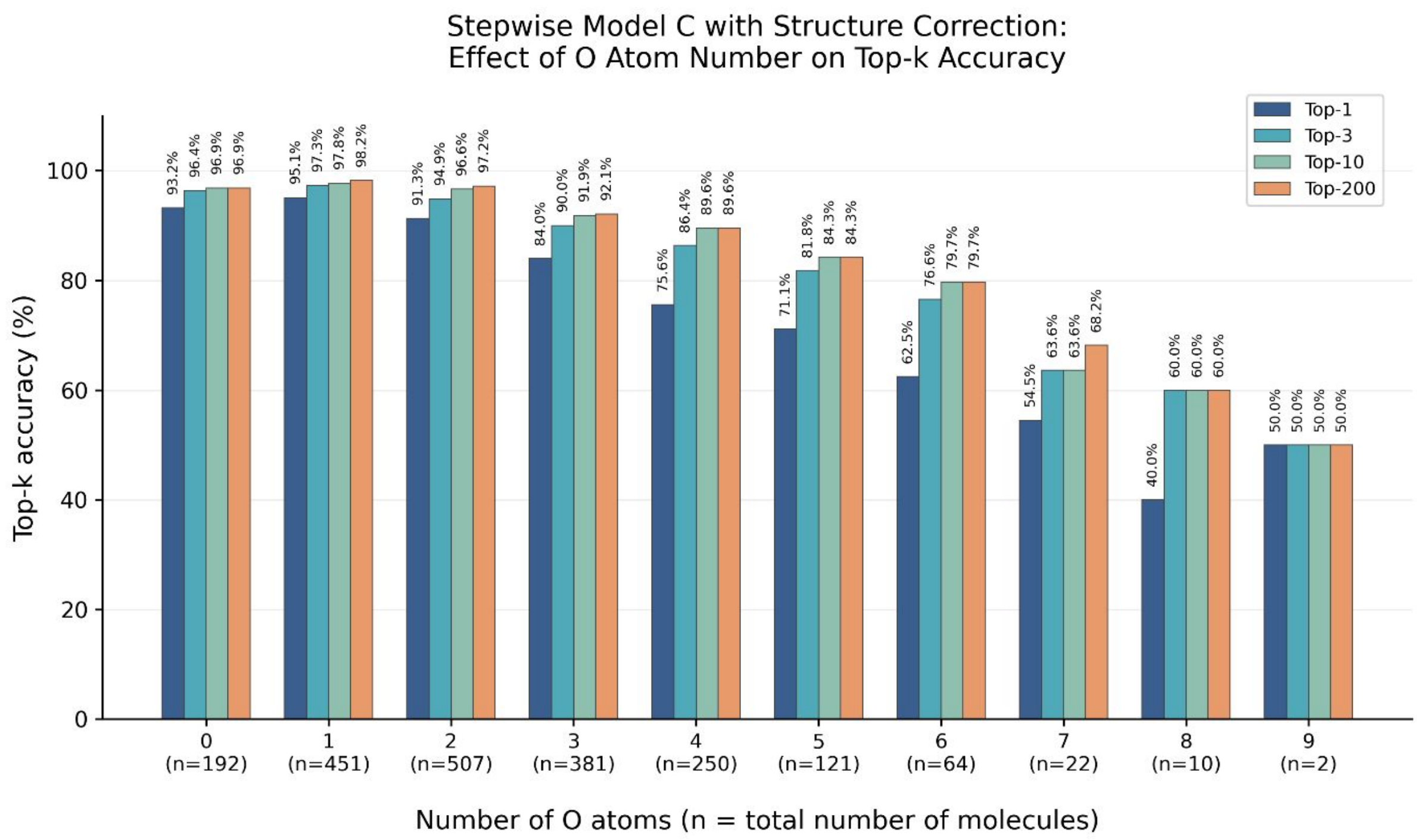

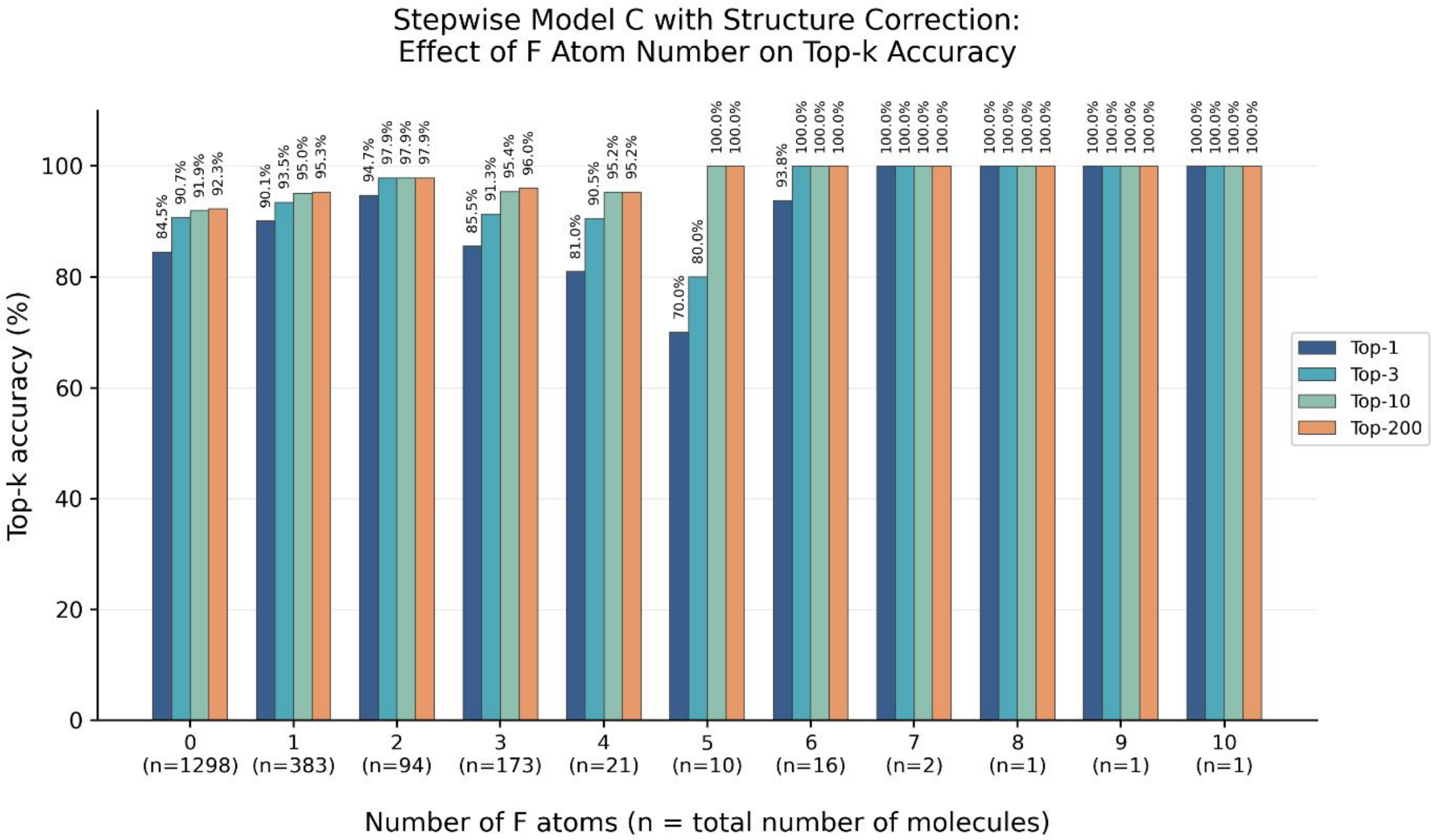
Stepwise Model C with Structure Correction:
Effect of F Atom Number on Top-k Accuracy
Top-k accuracy (%)
0
20
40
60
80
100
84.5% 90.7% 91.9% 92.3%
90.1% 93.5% 95.0% 95.3%
94.7% 97.9% 97.9% 97.9%
85.5% 91.3% 95.4% 96.0%
81.0% 90.5% 95.2% 95.2%
70.0% 80.0% 100.0% 100.0%
93.8% 100.0% 100.0% 100.0%
100.0% 100.0% 100.0% 100.0%
100.0% 100.0% 100.0% 100.0%
100.0% 100.0% 100.0% 100.0%
100.0% 100.0% 100.0% 100.0%
Top-1
Top-3
Top-10
Top-200
0 (n=1298)
1 (n=383)
2 (n=94)
3 (n=173)
4 (n=21)
5 (n=10)
6 (n=16)
7 (n=2)
8 (n=1)
9 (n=1)
10 (n=1)
Number of F atoms (n = total number of molecules)

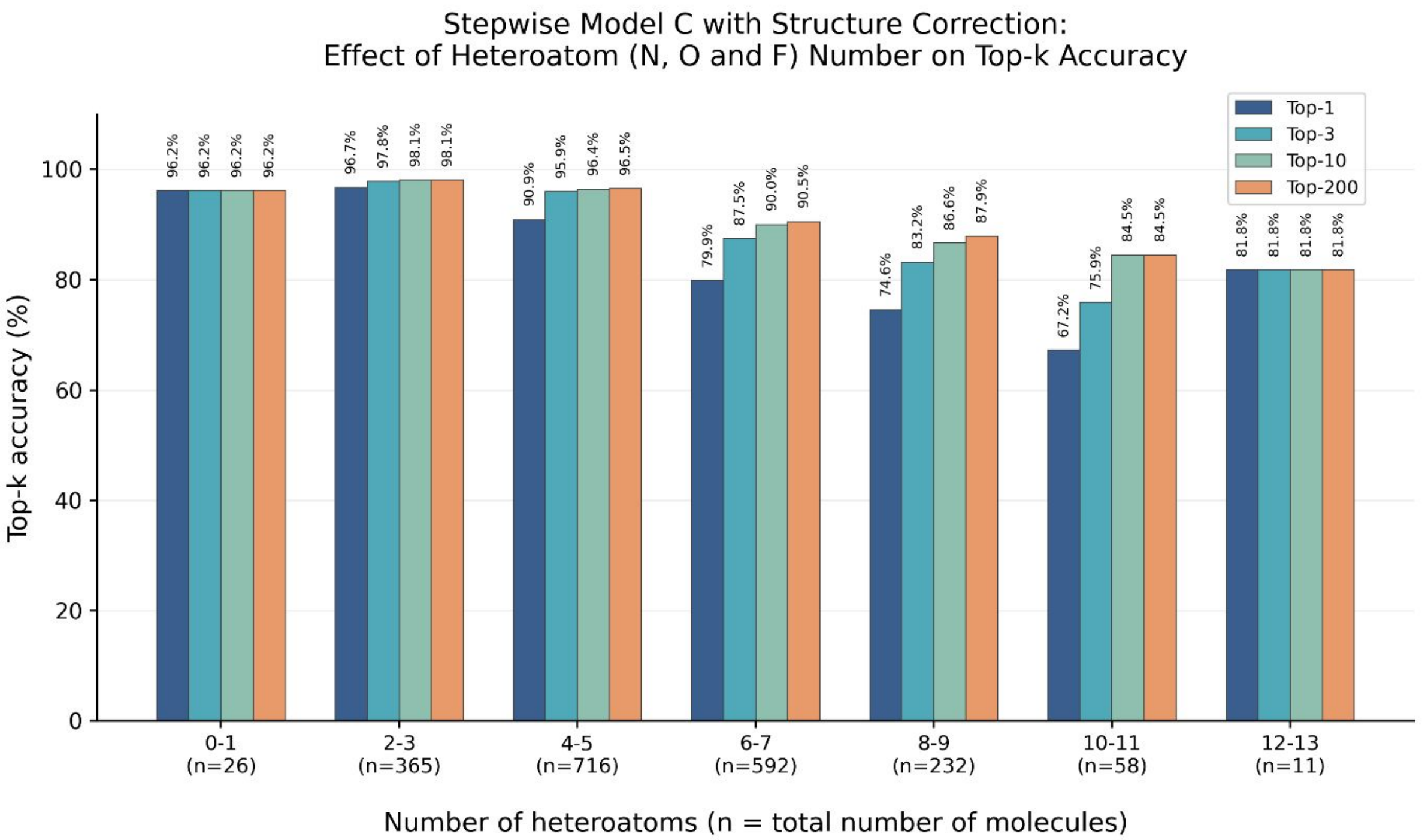
Stepwise Model C with Structure Correction:
Effect of Heteroatom (N, O and F) Number on Top-k Accuracy
Top-k accuracy (%)
0
20
40
60
80
100
Top-1
Top-3
Top-10
Top-200
96.2% 96.2% 96.2% 96.2%
96.7% 97.8% 98.1% 98.1%
90.9% 95.9% 96.4% 96.5%
79.9% 87.5% 90.0% 90.5%
74.6% 83.2% 86.6% 87.9%
67.2% 75.9% 84.5% 84.5%
81.8% 81.8% 81.8% 81.8%
0-1 (n=26)
2-3 (n=365)
4-5 (n=716)
6-7 (n=592)
8-9 (n=232)
10-11 (n=58)
12-13 (n=11)
Number of heteroatoms (n = total number of molecules)

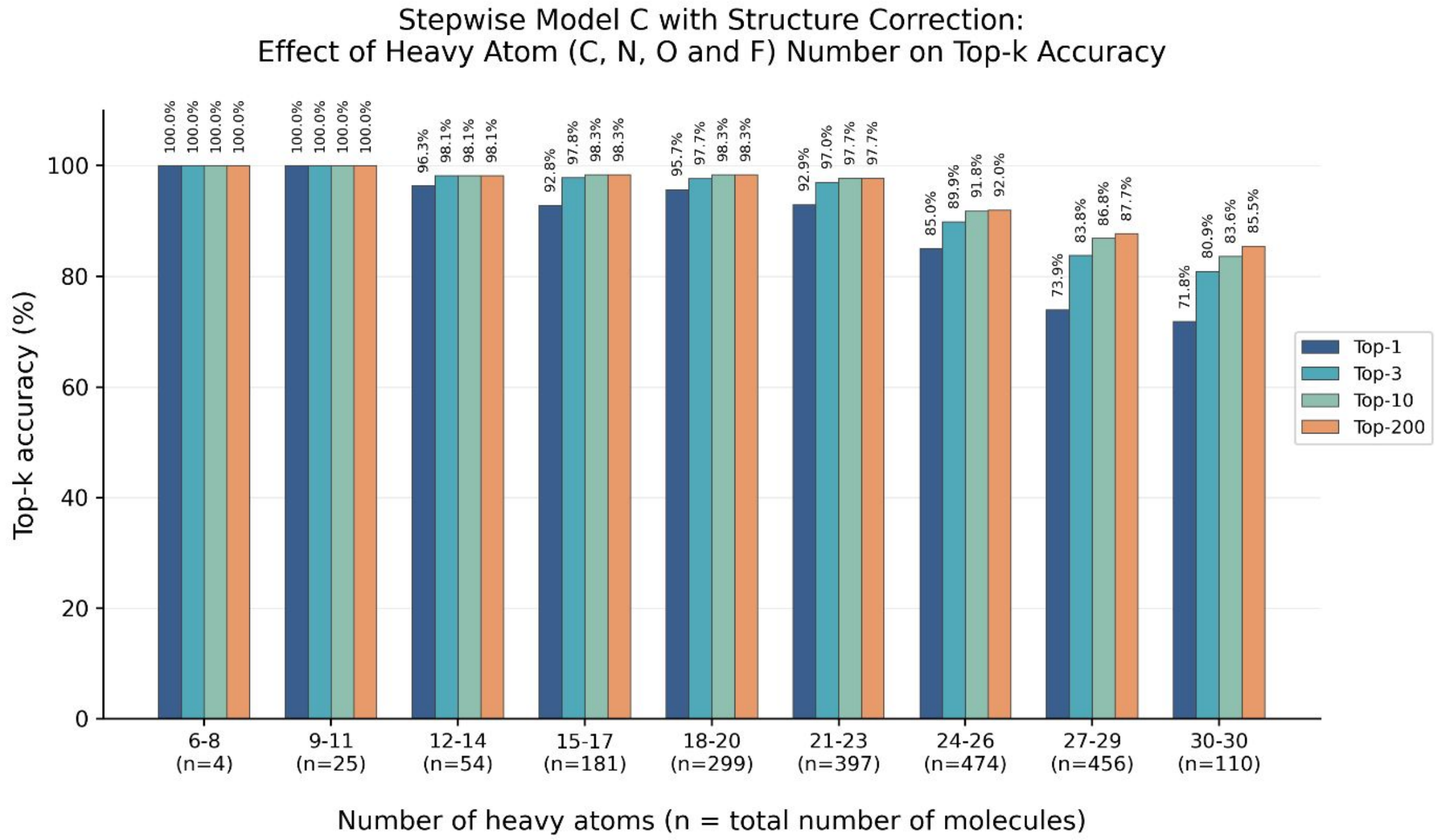
Stepwise Model C with Structure Correction:
Effect of Heavy Atom (C, N, O and F) Number on Top-k Accuracy
Top-k accuracy (%)
Number of heavy atoms (n = total number of molecules)
6-8 (n=4)
9-11 (n=25)
12-14 (n=54)
15-17 (n=181)
18-20 (n=299)
21-23 (n=397)
24-26 (n=474)
27-29 (n=456)
30-30 (n=110)
Top-1
Top-3
Top-10
Top-200
100.0% 100.0% 100.0% 100.0%
100.0% 100.0% 100.0% 100.0%
96.3% 98.1% 98.1% 98.1%
92.8% 97.8% 98.3% 98.3%
95.7% 97.7% 98.3% 98.3%
92.9% 97.0% 97.7% 97.7%
85.0% 89.9% 91.8% 92.0%
73.9% 83.8% 86.8% 87.7%
71.8% 80.9% 83.6% 85.5%

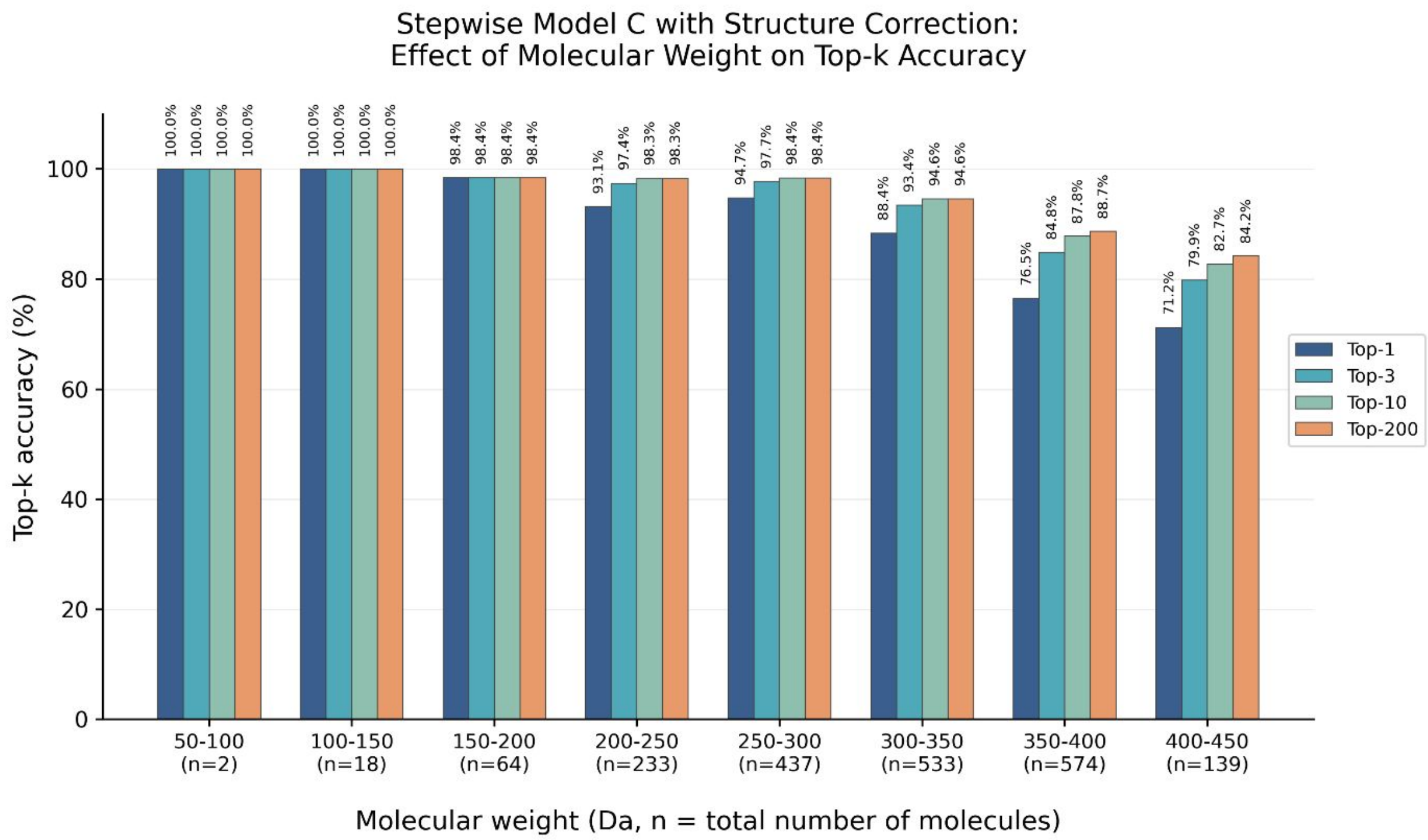
Stepwise Model C with Structure Correction:
Effect of Molecular Weight on Top-k Accuracy
Top-k accuracy (%)
Molecular weight (Da, n = total number of molecules)
50-100 (n=2)
100-150 (n=18)
150-200 (n=64)
200-250 (n=233)
250-300 (n=437)
300-350 (n=533)
350-400 (n=574)
400-450 (n=139)
Top-1
Top-3
Top-10
Top-200
100.0% 100.0% 100.0% 100.0%
100.0% 100.0% 100.0% 100.0%
98.4% 98.4% 98.4% 98.4%
93.1% 97.4% 98.3% 98.3%
94.7% 97.7% 98.4% 98.4%
88.4% 93.4% 94.6% 94.6%
76.5% 84.8% 87.8% 88.7%
71.2% 79.9% 82.7% 84.2%

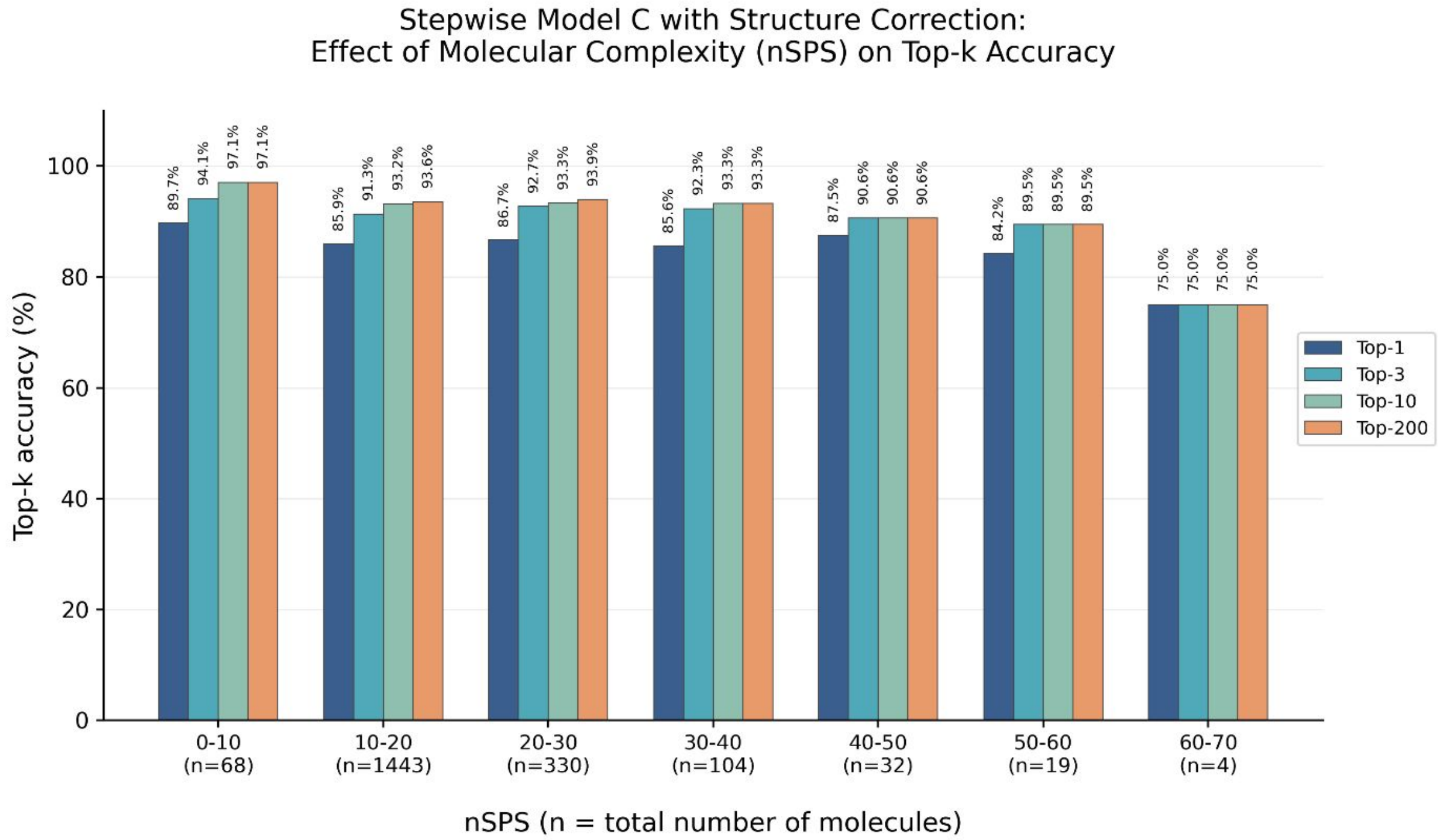

Stepwise Model C with Structure Correction:
Effect of Molecular Complexity (nSPS) on Top-k Accuracy
Top-k accuracy (%)
0
20
40
60
80
100
89.7%
94.1%
97.1%
97.1%
85.9%
91.3%
93.2%
93.6%
86.7%
92.7%
93.3%
93.9%
85.6%
92.3%
93.3%
93.3%
87.5%
90.6%
90.6%
90.6%
84.2%
89.5%
89.5%
89.5%
75.0%
75.0%
75.0%
75.0%
0-10 (n=68)
10-20 (n=1443)
20-30 (n=330)
30-40 (n=104)
40-50 (n=32)
50-60 (n=19)
60-70 (n=4)
nSPS (n = total number of molecules)
Top-1
Top-3
Top-10
Top-200

2.4 Application of Inverse-IMPRESSION on experimental molecules

The inverse-IMPRESSION platform was applied to predict the structures of 19 molecules using experimental ^{1}H and ^{13}C chemical shifts together with coupling constants obtained from COSY, HSQC, and HMBC spectra. Unless otherwise specified, 2000 sets of NMR noises were added to each molecule, with standard deviations of 0.3 ppm for ^{1}H, 2.5 ppm for ^{13}C, and 0.4 Hz for all coupling constants. The predicted candidate structures were ranked by frequency, defined as the proportion of times a structure was predicted among the 2000 candidates.

An additional ranking method based on the MAE of ^{13}C chemical shifts was also employed to improve performance. For each chemically valid candidate structure, 20 conformers were generated and optimised using the MMFF force field as implemented in RDKit. The ^{13}C chemical shifts of each conformer were then predicted using the IMPRESSION-G2 model. Boltzmann-weighted averaging of the conformers was performed to obtain the final predicted ^{13}C chemical shifts for each candidate structure. The mean absolute error (MAE) over all carbon atoms was subsequently calculated and used to rank the valid candidates.

The success of the ^{13}C chemical shift MAE ranking as a confidence metric can be seen in the following plot, where the top-ranked candidates in unsuccessful elucidations are clearly seen to be unreasonable based on MAE, while the top-ranked candidates for successful elucidations have MAEs in line with the expected accuracy of the forward IMPRESSION-G2 model.

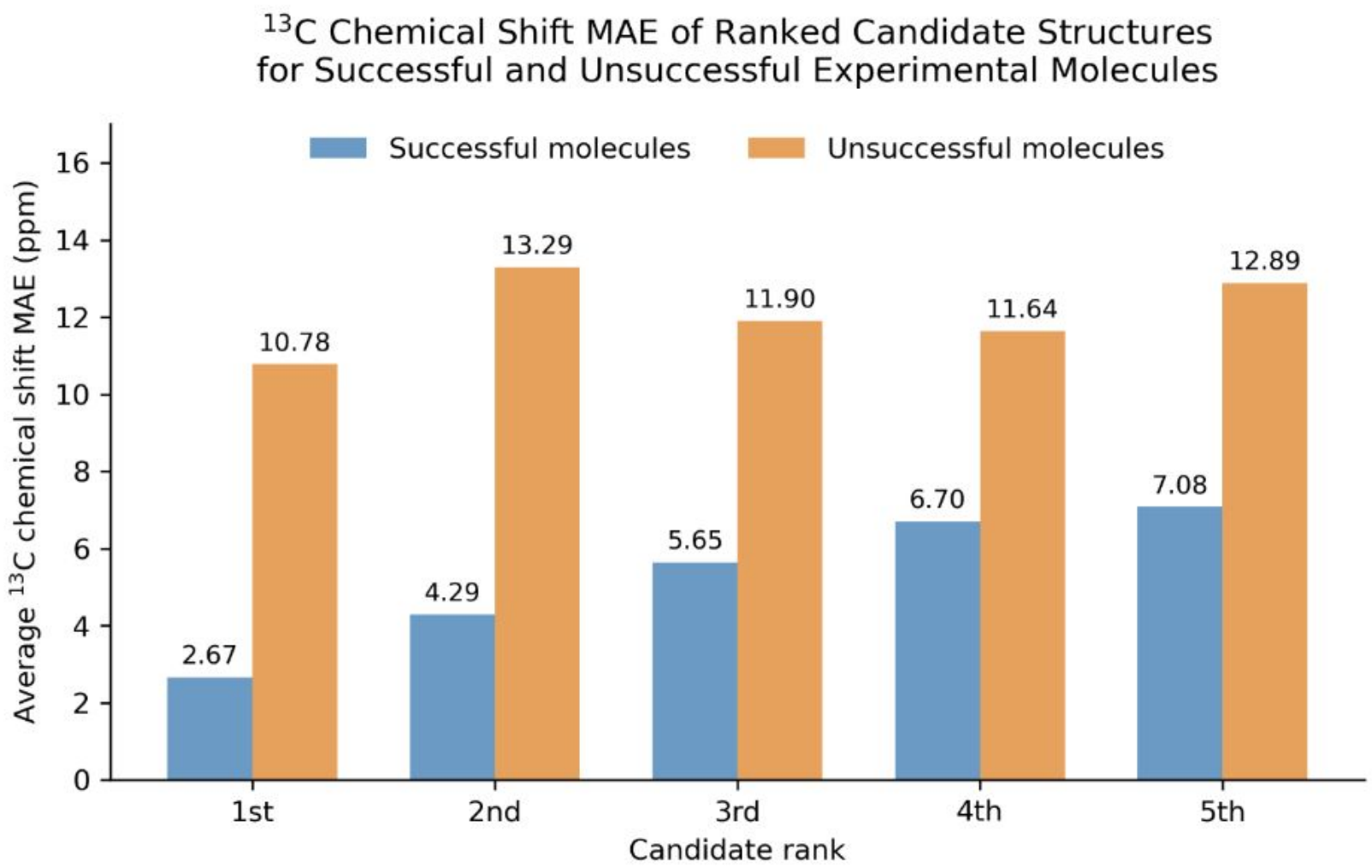

The structures of the 19 molecules are shown below and classified into two categories: (1) correctly predicted structures ranked first by MAE; and (2) cases where no valid structures were predicted or where the top-ranked candidate is not the correct structure.

Supplementary Table 4 summarises the molecular properties associated with prediction accuracy: (1) Number of heteroatoms; (2) Number of quaternary-quaternary C-C bonds; (3) COSY ratio, defined as the proportion of 2JHH, 3JHH and 4JHH coupling constants among all H-H pairs separated by 2-4 bonds; (4) HSQC ratio, defined as the proportion of 1JCH coupling constants among all C-H bonds; and (5) HMBC ratio, defined as the proportion of 2JCH, 3JCH and 4JCH coupling constants among all C-H pairs separated by 2-4 bonds.

2.4.1 Correct Predictions (Top MAE Rank)

**Structure**	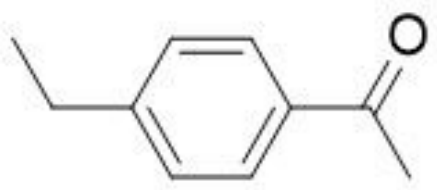	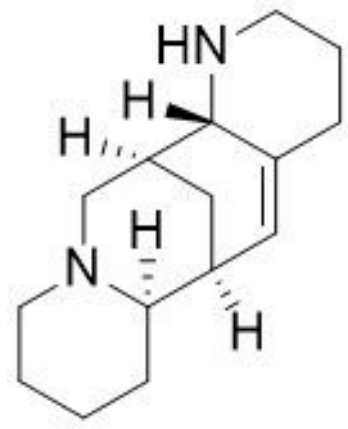
**Name**	1-(4-ethylphenyl)ethenone	aloperine
**Formula**	$C_{10}H_{12}O$	$C_{15}H_{24}N_2$
**Molecular weight**	148.2 g/mol	232.4 g/mol
**nSPS (SPS)**	9.6 (106)	45.8 (779)
**Number of NMR noise sets**	2000	2000
**Coupling constant noise**	0.4 Hz	0.4 Hz
**^{13}C MAE**	1.99 ppm	2.94 ppm
**^{13}C MAE rank**	1	1
**Frequency**	99.85%	80.5%
**Frequency rank**	1	1
**Total prediction time**	17.9 min	70.5 min
**Prediction time per shot**	0.5 s	2.1 s

Structure	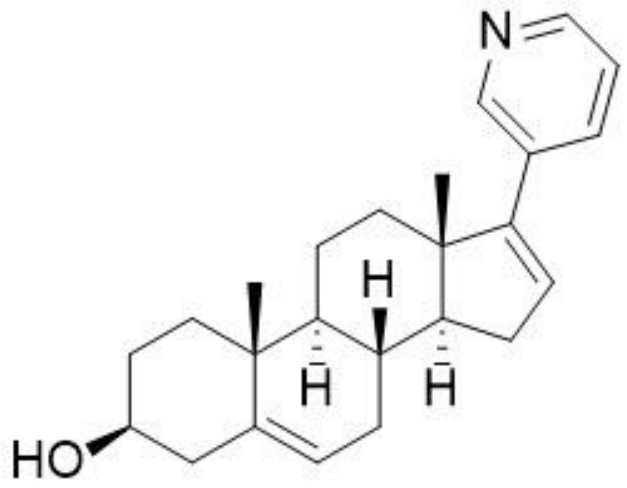	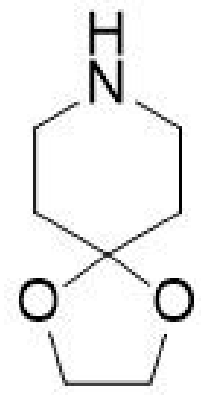
**Name**	abiraterone	4-piperidone ethylene acetal
**Formula**	$C_{24}H_{31}NO$	$C_7H_{13}NO_2$
**Molecular weight**	349.5 g/mol	143.2 g/mol
**nSPS (SPS)**	44.4 (1154)	31.2 (312)
**Number of NMR noise sets**	2000	2000
**Coupling constant noise**	0.4 Hz	0.4 Hz
**^{13}C MAE**	2.99 ppm	1.27 ppm
**^{13}C MAE rank**	1	1
**Frequency**	1.5%	0.85%
**Frequency rank**	2	8
**Total prediction time**	155.5 min	31.2 min
**Prediction time per shot**	4.7 s	0.9 s

Structure	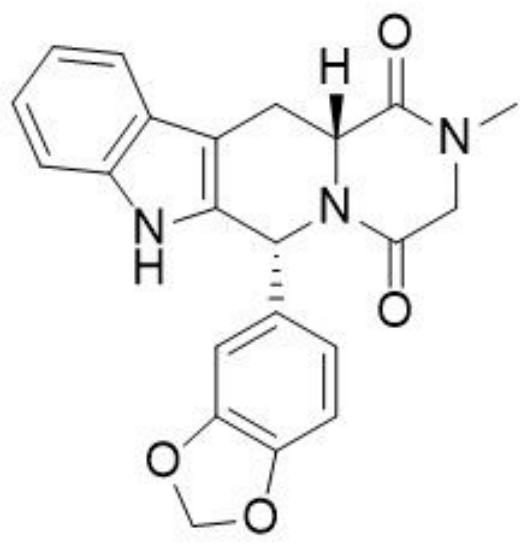	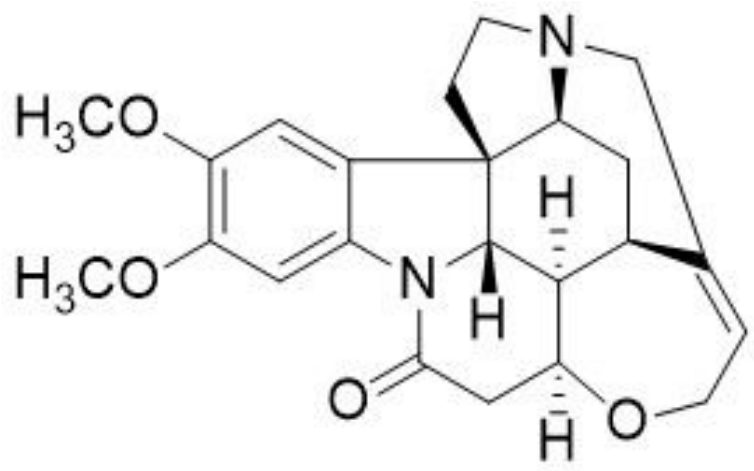
**Name**	tadalafil	brucine
**Formula**	$C_{22}H_{19}N_3O_4$	$C_{23}H_{26}N_2O_4$
**Molecular weight**	389.4 g/mol	394.5 g/mol
**nSPS (SPS)**	22.8 (661)	41.0 (1189)
**Number of NMR noise sets**	2000	5000
**Coupling constant noise**	0.4 Hz	0.4 Hz
**^{13}C MAE**	2.08 ppm	4.31 ppm
**^{13}C MAE rank**	1	1
**Frequency**	0.25 %	0.05%
**Frequency rank**	1	1
**Total prediction time**	166.7 min	423.4 min
**Prediction time per shot**	5.0 s	5.1 s

**Structure**	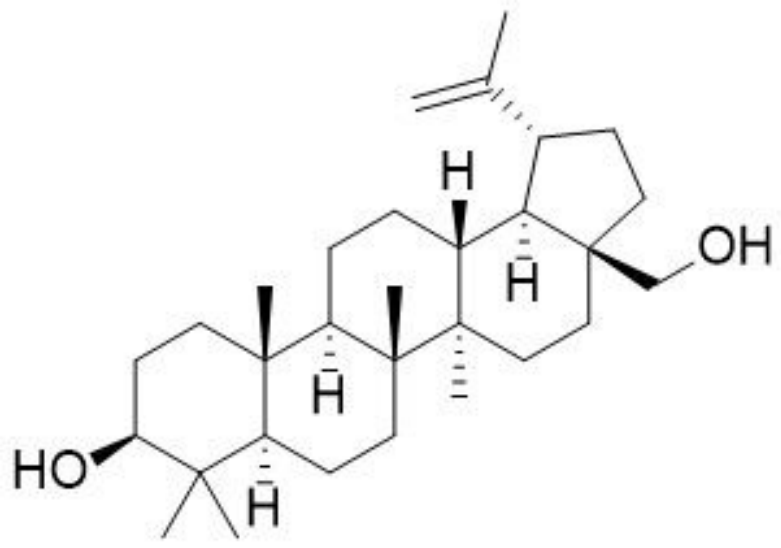	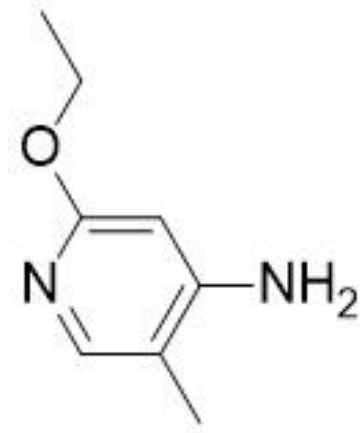
**Name**	betulin	2-ethoxy-5-methylpyridin-4-amine
**Formula**	$C_{30}H_{50}O_2$	$C_8H_{12}N_2O$
**Molecular weight**	442.7 g/mol	152.2 g/mol
**nSPS (SPS)**	56.5 (1808)	9.6 (106)
**Number of NMR noise sets**	2000	2000
**Coupling constant noise**	0.4 Hz	0.6 Hz
**^{13}C MAE**	3.83 ppm	1.71 ppm
**^{13}C MAE rank**	1	1
**Frequency**	0.1%	0.7%
**Frequency rank**	1	7
**Total prediction time**	234.1 min	33.6 min
**Prediction time per shot**	7.0 s	1.0 s

**Structure**	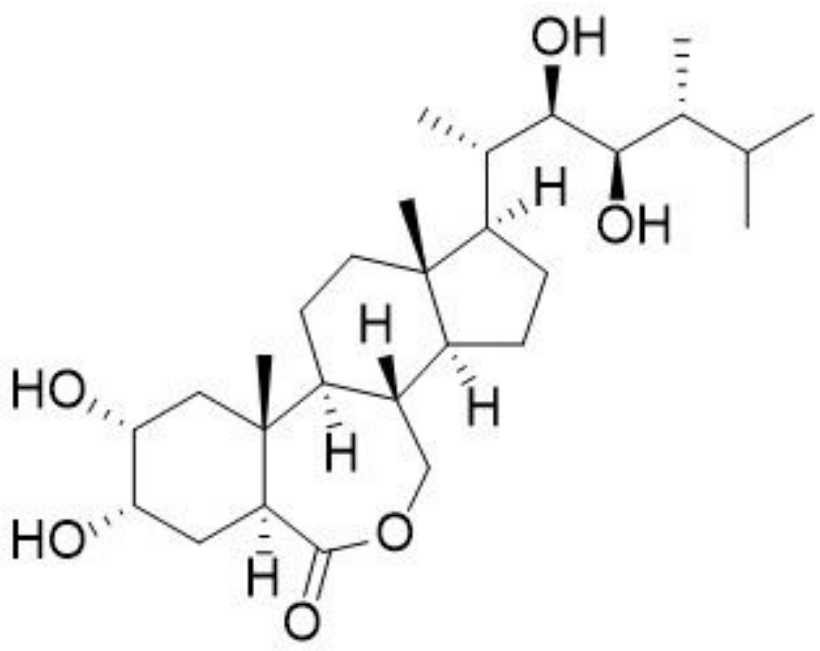	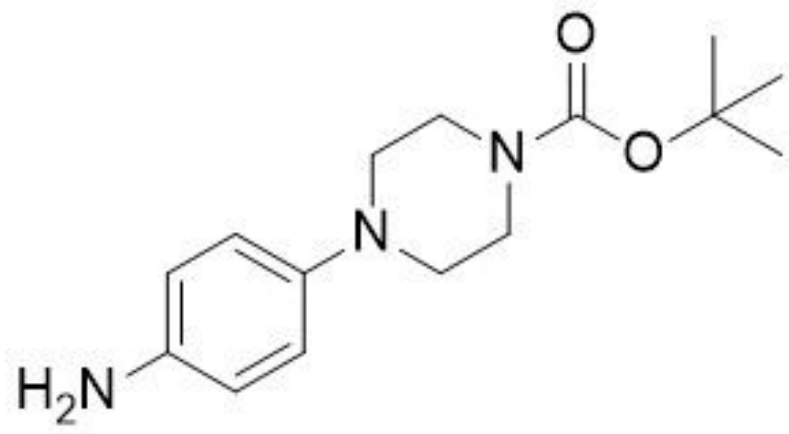
**Name**	24-epibrassinolide	1-Boc-4-(4-aminophenyl)piperazine
**Formula**	$C_{28}H_{48}O_6$	$C_{15}H_{23}N_3O_2$
**Molecular weight**	480.7 g/mol	277.4 g/mol
**nSPS (SPS)**	48.1 (1635)	16.2 (324)
**Number of NMR noise sets**	2000	2000
**Coupling constant noise**	0.4 Hz	0.6 Hz
**^{13}C MAE**	3.66 ppm	2.00 ppm
**^{13}C MAE rank**	1	1
**Frequency**	0.35%	0.05%
**Frequency rank**	1	10
**Total prediction time**	238.9 min	81.3 min
**Prediction time per shot**	7.2 s	2.4 s

**Structure**	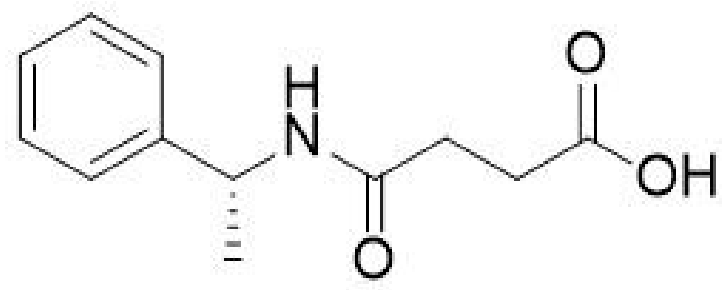
**Name**	(R)-(+)-N-(1-phenylethyl)succinamic acid
**Formula**	$C_{12}H_{15}NO_3$
**Molecular weight**	221.3 g/mol
**nSPS (SPS)**	11.8 (189)
**Number of NMR noise sets**	2000
**Coupling constant noise**	0.4 Hz
**^{13}C MAE**	2.55 ppm
**^{13}C MAE rank**	1
**Frequency**	0.25%
**Frequency rank**	1
**Total prediction time**	88.9 min
**Prediction time per shot**	2.7 s

2.4.2 Incorrect Predictions

**Structure**	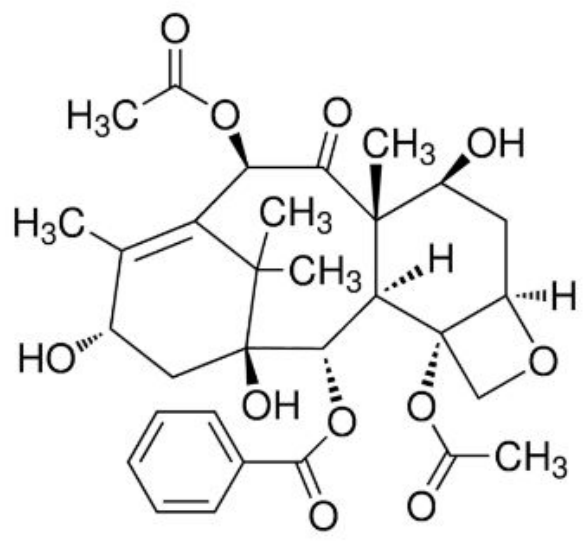	**-**
**Name**	baccatin-III	-
**Formula**	$C_{31}H_{38}O_{11}$	-
**Molecular weight**	586.6 g/mol	-
**nSPS**	40.4 (1697)	-
**Number of NMR noise sets**	-	2000
**Coupling constant noise**	-	0.4 Hz
**^{13}C MAE**	-	-
**^{13}C MAE rank**	-	-
**Frequency**	-	-
**Frequency rank**	-	-
**Total prediction time**	-	216.7 min
**Prediction time per shot**	-	6.5 s

Structure	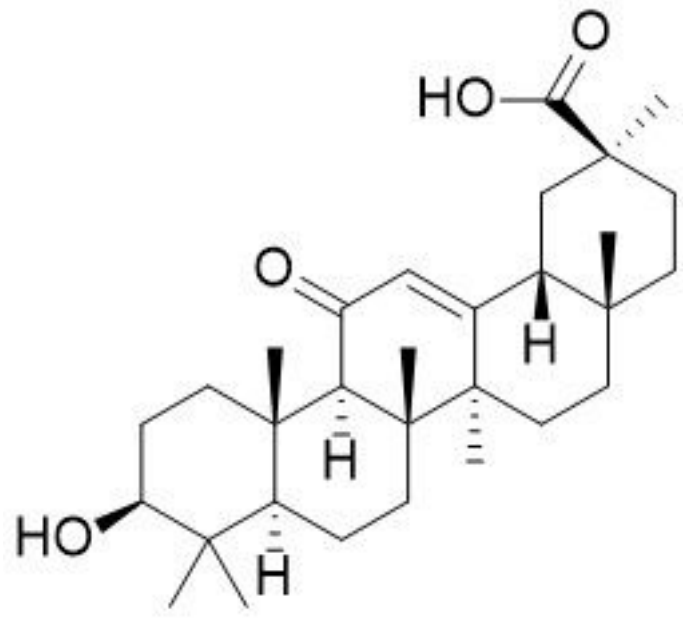	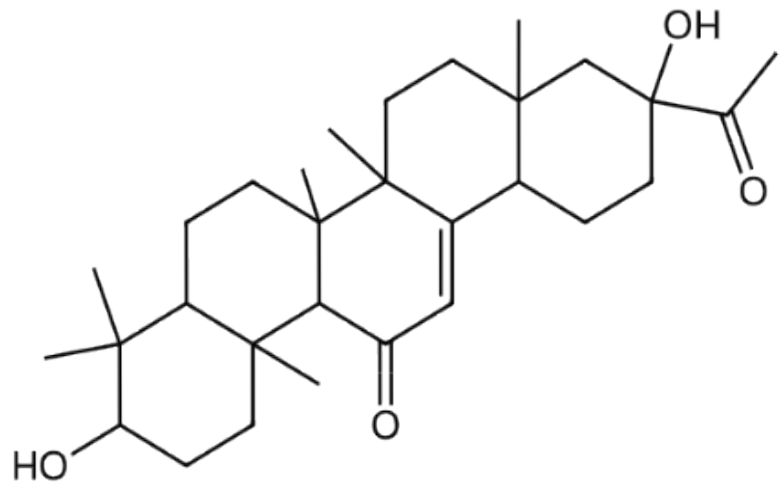
**Name**	enoxolone	Wrongly predicted candidate ranked #1 by ^{13}C MAE
**Formula**	$C_{30}H_{46}O_4$	$C_{30}H_{46}O_4$
**Molecular weight**	470.7 g/mol	470.7 g/mol
**nSPS (SPS)**	54.1 (1839)	-
**Number of NMR noise sets**	-	2000
**Coupling constant noise**	-	0.4 Hz
**^{13}C MAE**	-	5.75 ppm
**^{13}C MAE rank**	-	1
**Frequency**	-	0.05%
**Frequency rank**	-	1
**Total prediction time**	-	245.1 min
**Prediction time per shot**	-	7.4 s

Structure	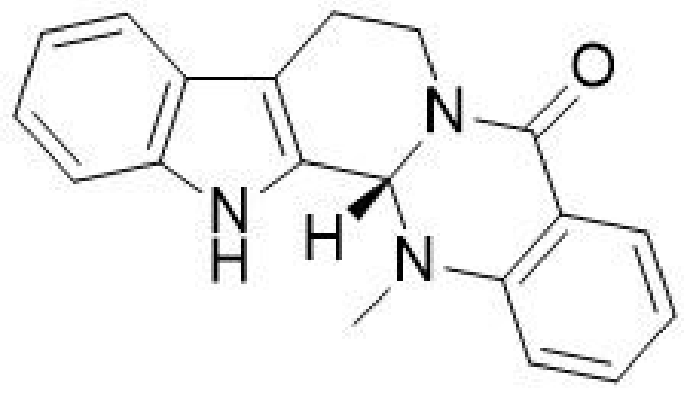	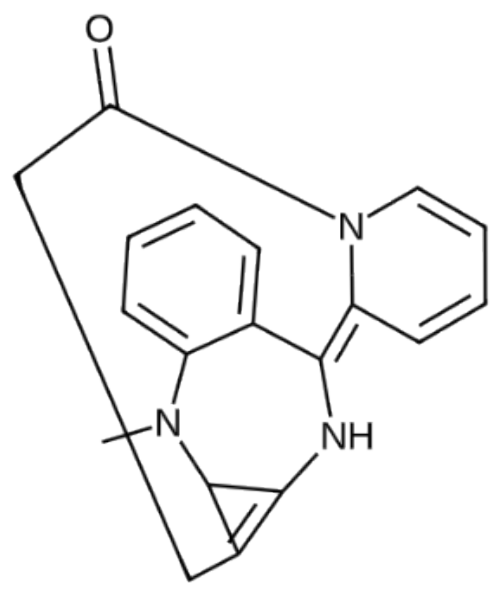
**Name**	evodiamine	Wrongly predicted candidate ranked #1 by ^{13}C MAE
**Formula**	$C_{19}H_{17}N_3O$	$C_{19}H_{17}N_3O$
**Molecular weight**	303.4 g/mol	303.4 g/mol
**nSPS (SPS)**	19.5 (449)	-
**Number of NMR noise sets**	-	2000
**Coupling constant noise**	-	0.4 Hz
**^{13}C MAE**	-	8.63 ppm
**^{13}C MAE rank**	-	1
**Frequency**	-	0.05%
**Frequency rank**	-	1
**Total prediction time**	-	113.9 min
**Prediction time per shot**	-	3.4 s

Structure	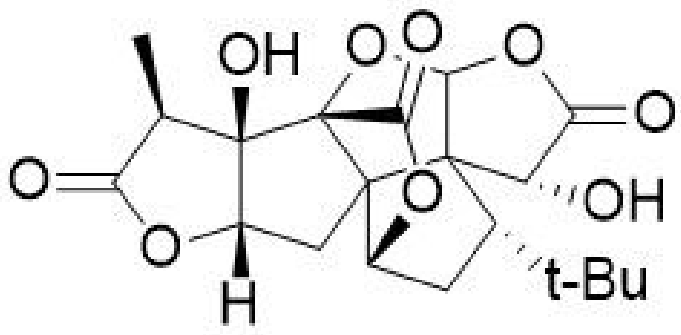	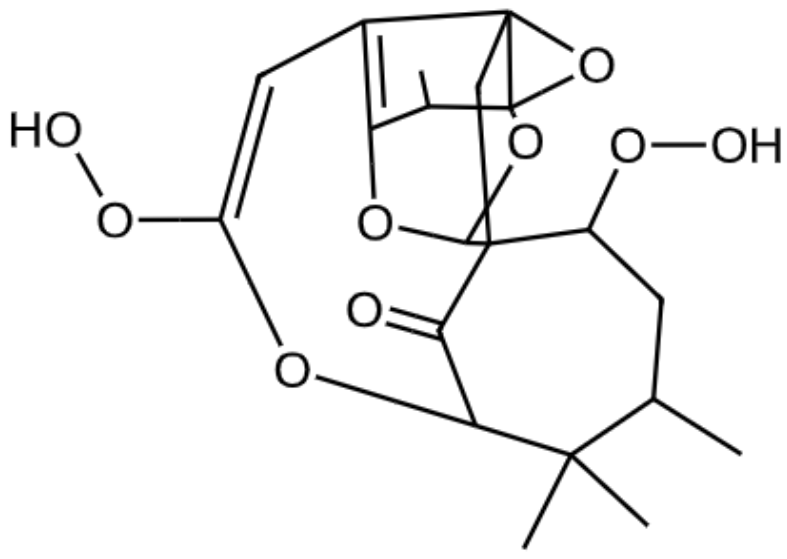
**Name**	ginkgolide A	Wrongly predicted candidate ranked #1 by ^{13}C MAE
**Formula**	$C_{20}H_{24}O_9$	$C_{20}H_{24}O_9$
**Molecular weight**	408.4 g/mol	408.4 g/mol
**nSPS (SPS)**	59.2 (1717)	-
**Number of NMR noise sets**	-	2000
**Coupling constant noise**	-	0.4 Hz
**^{13}C MAE**	**-**	12.58 ppm
**^{13}C MAE rank**	**-**	1
**Frequency**	**-**	0.05%
**Frequency rank**	**-**	1
**Total prediction time**	-	144.0 min
**Prediction time per shot**	-	4.3 s

**Structure**	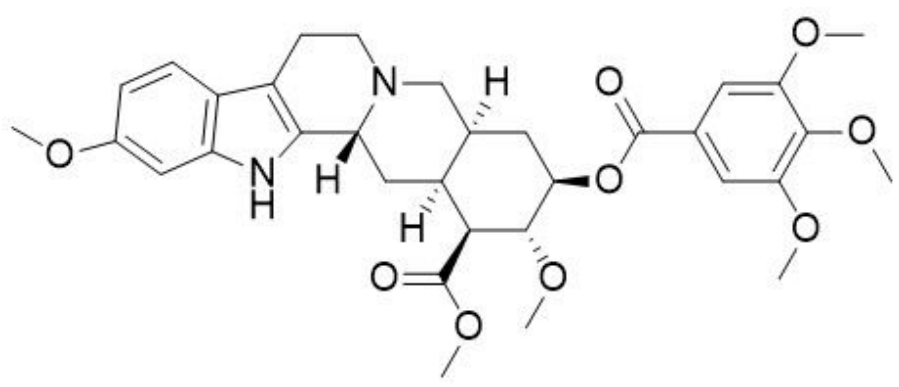	**-**
**Name**	reserpine	-
**Formula**	$C_{33}H_{40}N_2O_9$	-
**Molecular weight**	608.7 g/mol	-
**nSPS (SPS)**	26.1 (1148)	-
**Number of NMR noise sets**	-	-
**Coupling constant noise**	-	-
**^{13}C MAE**	-	-
**^{13}C MAE rank**	-	-
**Frequency**	-	-
**Frequency rank**	-	-
**Total prediction time**	-	255.9 min
**Prediction time per shot**	-	7.7 s

Structure	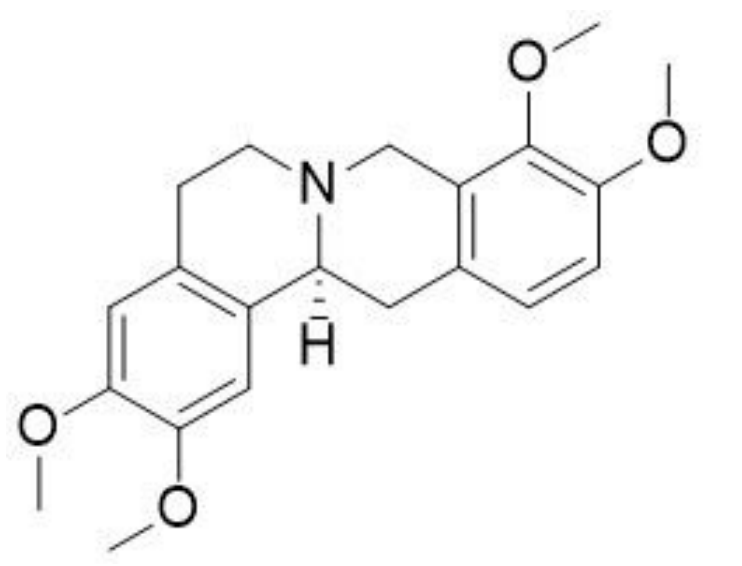	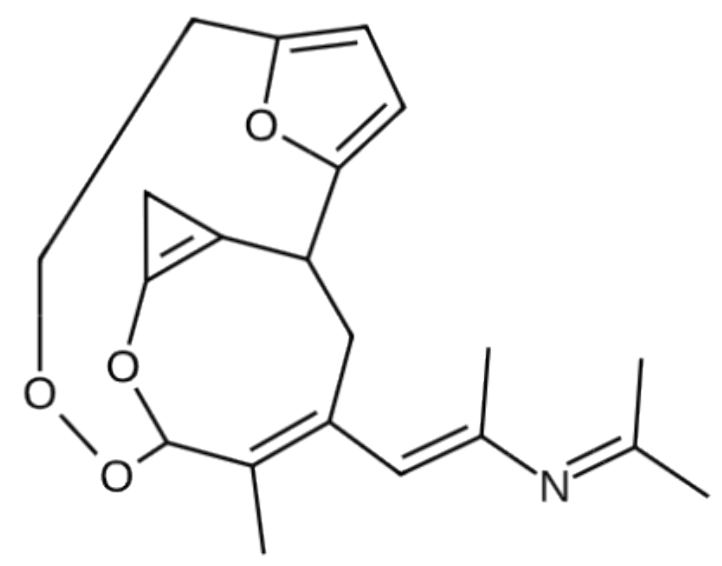
**Name**	tetrahydropalmatine	Wrongly predicted candidate ranked #1 by ^{13}C MAE
**Formula**	$C_{21}H_{25}NO_4$	$C_{21}H_{25}NO_4$
**Molecular weight**	355.5 g/mol	355.5 g/mol
**nSPS (SPS)**	18.4 (478)	-
**Number of NMR noise sets**	-	2000
**Coupling constant noise**	-	0.4 HZ
**^{13}C MAE**	-	16.15 ppm
**^{13}C MAE rank**	-	1
**Frequency**	-	0.05%
**Frequency rank**	-	1
**Total prediction time**	-	97.8 min
**Prediction time per shot**	-	2.9 s

Structure	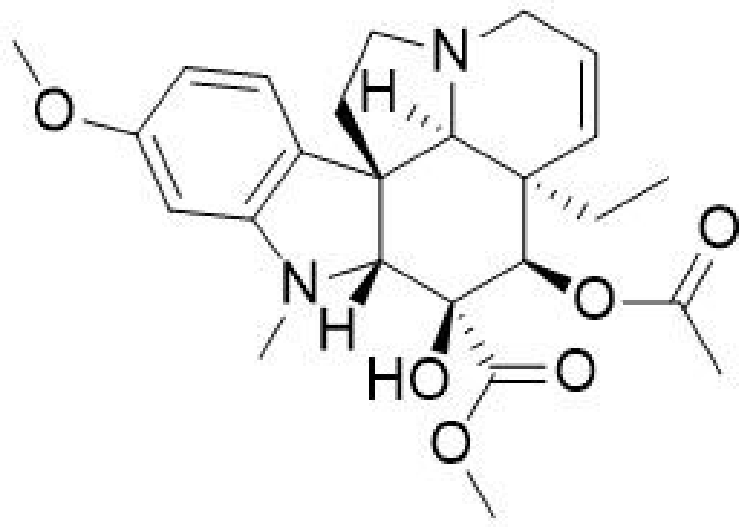	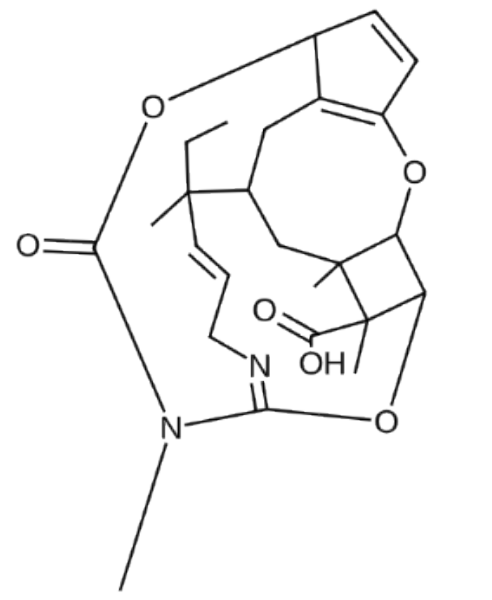
**Name**	vindoline	Wrongly predicted candidate ranked #1 by ^{13}C MAE
**Formula**	$C_{25}H_{32}N_2O_6$	$C_{25}H_{32}N_2O_6$
**Molecular weight**	456.5 g/mol	456.5 g/mol
**nSPS (SPS)**	38.4 (1267)	-
**Number of NMR noise sets**	-	2000
**Coupling constant noise**	-	0.4 Hz
**^{13}C MAE**	-	11.51 ppm
**^{13}C MAE rank**	-	1
**Frequency**	-	0.05%
**Frequency rank**	-	1
**Total prediction time**	-	147.3 min
**Prediction time per shot**	-	4.4 s

Structure	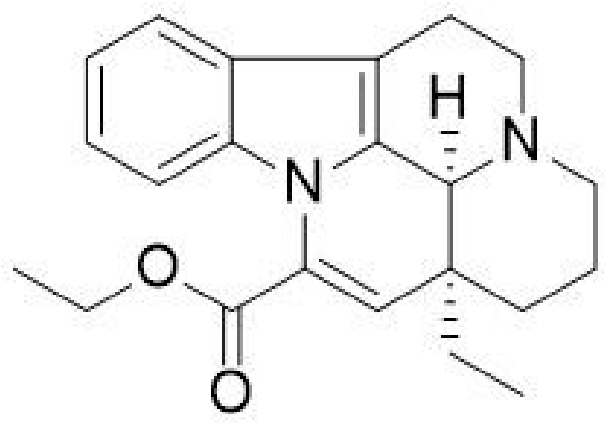	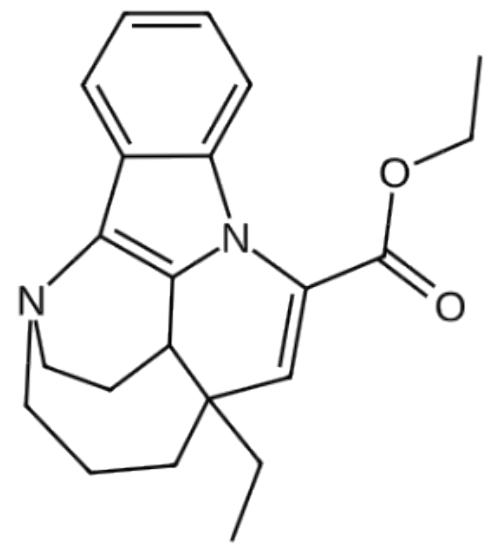
**Name**	vinpocetine	Wrongly predicted candidate ranked #1 by ^{13}C MAE
**Formula**	$C_{22}H_{26}N_2O_2$	$C_{22}H_{26}N_2O_2$
**Molecular weight**	350.5 g/mol	350.5 g/mol
**nSPS (SPS)**	27.2 (707)	-
**Number of NMR noise sets**	-	2000
**Coupling constant noise**	-	0.4 Hz
**^{13}C MAE**	-	5.38 ppm
**^{13}C MAE rank**	-	1
**Frequency**	-	0.05%
**Frequency rank**	-	1
**Total prediction time**	-	209.4 min
**Prediction time per shot**	-	6.3 s

2.4.3 Properties of Experimental molecules

Supplementary Table 4. Molecular properties associated with prediction accuracy for 19 experimental molecules. Molecules highlighted in blue are correctly predicted. Values highlighted in red indicate deviations from the defined thresholds for successful prediction: (1) Heteroatom count ≤ 7; (2) Quaternary-quaternary C-C bond count ≤ 4; and (3) COSY/HMBC ratio ≥ 15%.

Molecule name	M_R (g/mol)	Heteroatom count	Quaternary-quaternary C-C bond count	COSY ratio (%)	HSQC ratio (%)	HMBC ratio (%)
1-(4-ethylphenyl)ethenone	148.2	1	1	38.1	100.0	29.4
aloperine	232.4	2	0	44.0	100.0	39.2
abiraterone	349.5	2	3	35.6	100.0	29.5
4-piperidone ethylene acetal	143.2	3	0	23.5	100.0	52.5
tadalafil	389.4	7	4	50.0	100.0	35.4
brucine	394.5	6	3	30.9	100.0	34.9
betulin	442.7	2	1	22.1	100.0	19.0
2-ethoxy-5-methylpyridin-4-amine	152.2	3	1	31.6	100.0	40.4
24-epibrassinolide	480.7	6	0	25.6	100.0	20.7
1-Boc-4-(4-aminophenyl)piperazine	277.4	5	0	15.4	100.0	40.7
(R)-(+)-N-(1-phenylethyl)succinamic acid	221.3	4	0	10.0	100.0	32.9
baccatin-III	586.6	11	5	12.5	100.0	32.7
enoxolone	470.7	4	3	13.5	100.0	39.1
evodiamine	303.4	4	5	61.5	100.0	30.2
ginkgolide A	408.4	9	4	13.0	100.0	20.4
reserpine	608.7	11	6	21.1	100.0	20.9
tetrahydropalmatine	355.5	5	5	27.8	100.0	21.3
vindoline	456.5	8	3	25.0	100.0	22.7
vinpocetine	350.5	4	4	49.3	100.0	31.4

3 Figures for dataset distributions

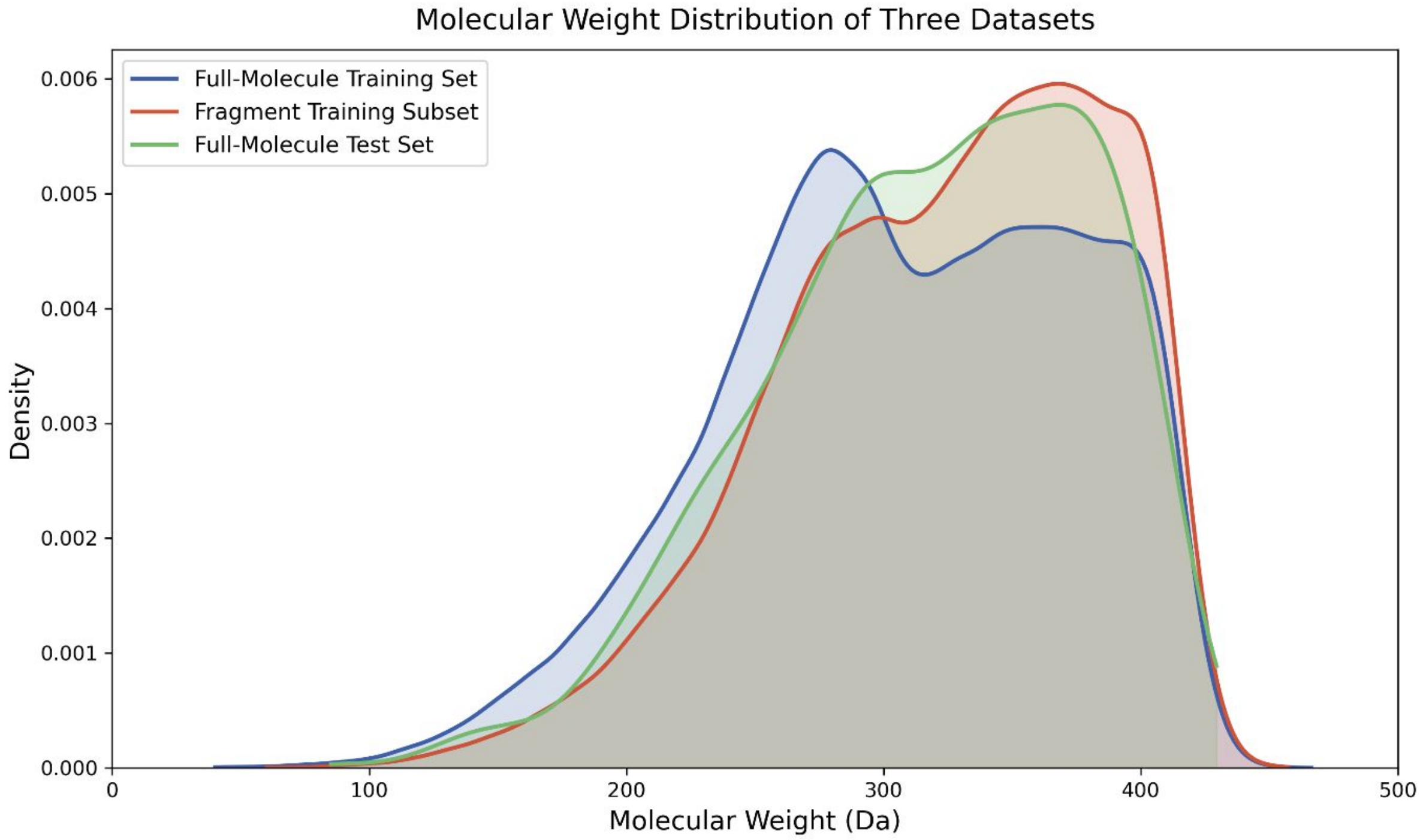

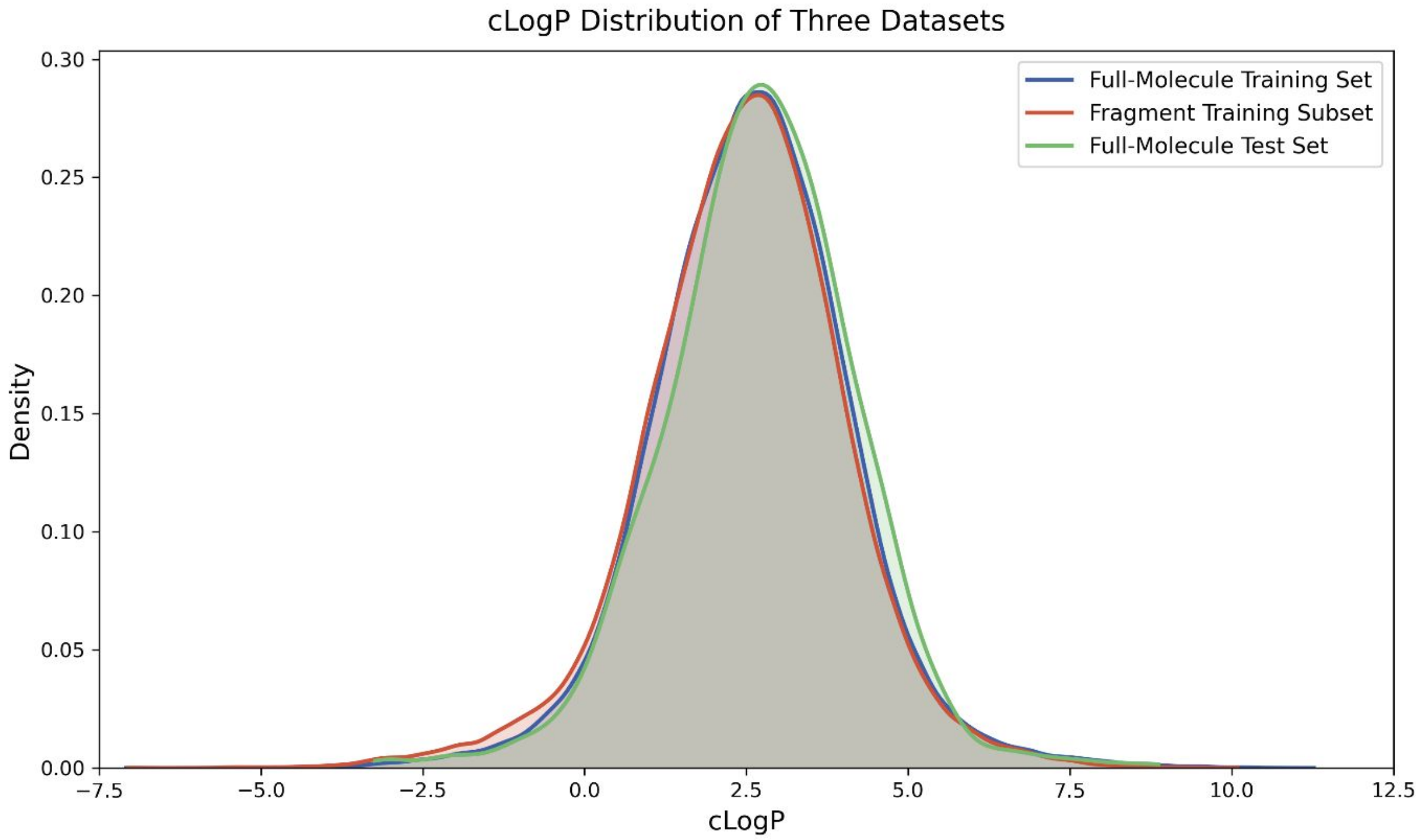

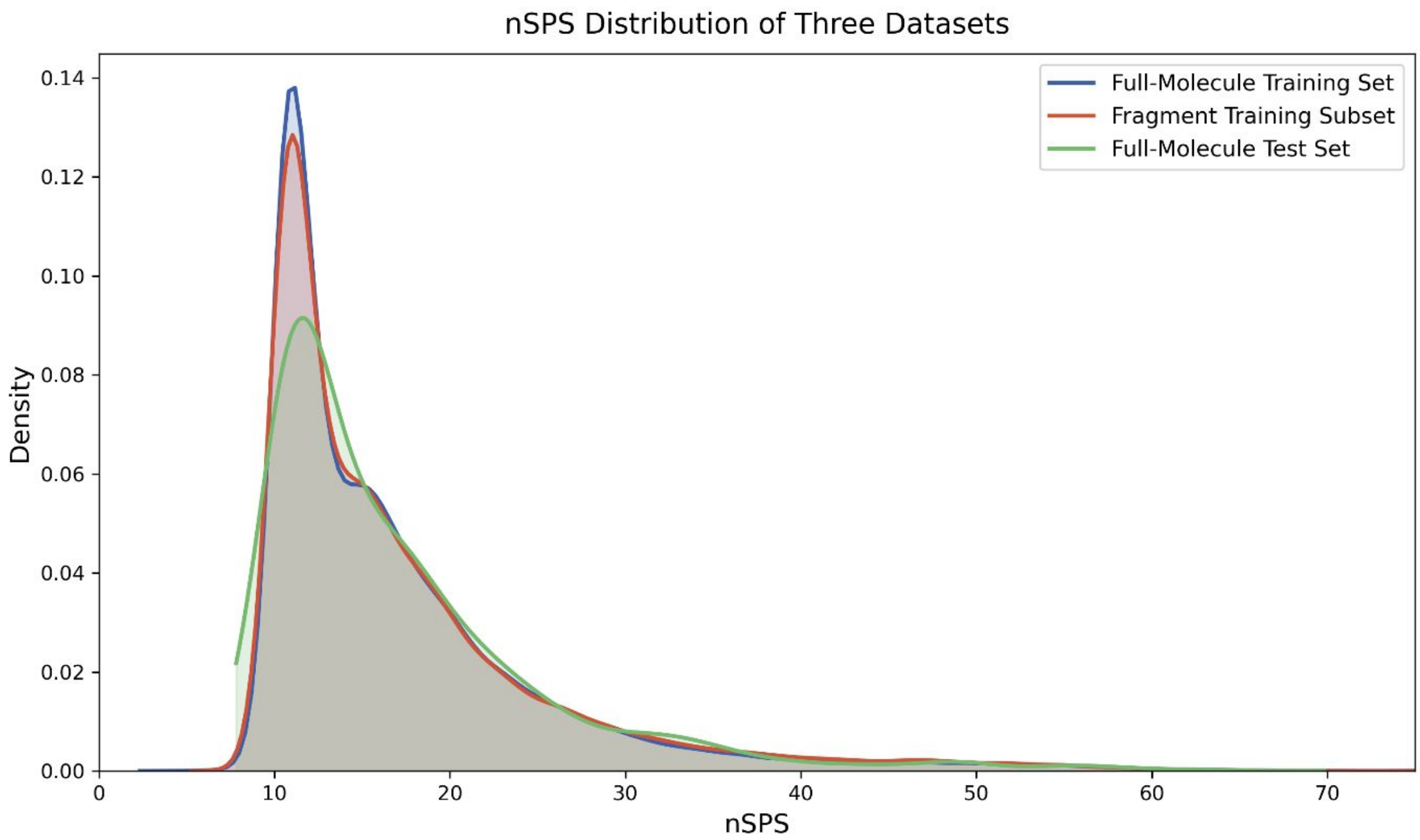
nSPS Distribution of Three Datasets
Full-Molecule Training Set
Fragment Training Subset
Full-Molecule Test Set
Density
nSPS

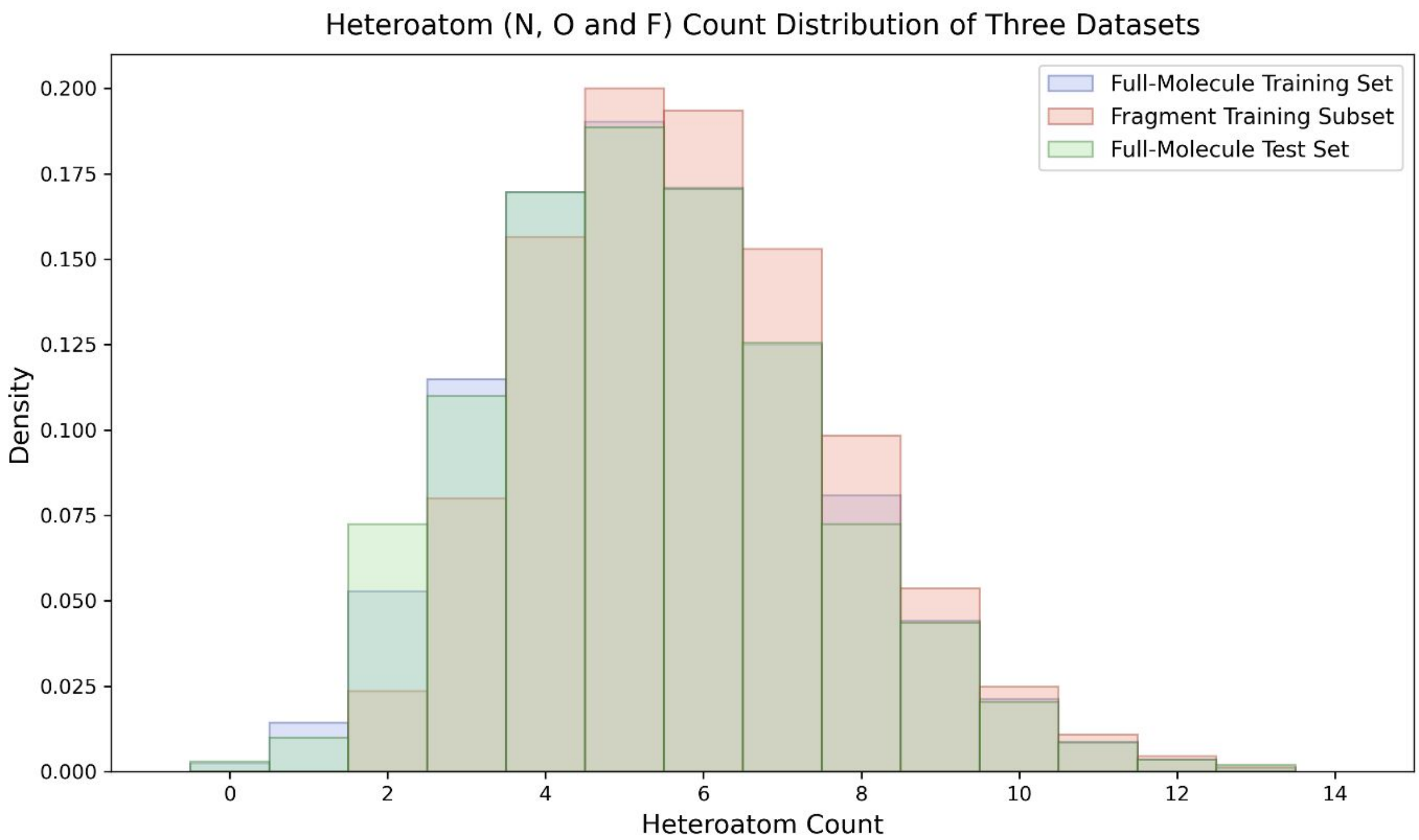
Heteroatom (N, O and F) Count Distribution of Three Datasets
Full-Molecule Training Set
Fragment Training Subset
Full-Molecule Test Set
Density
Heteroatom Count

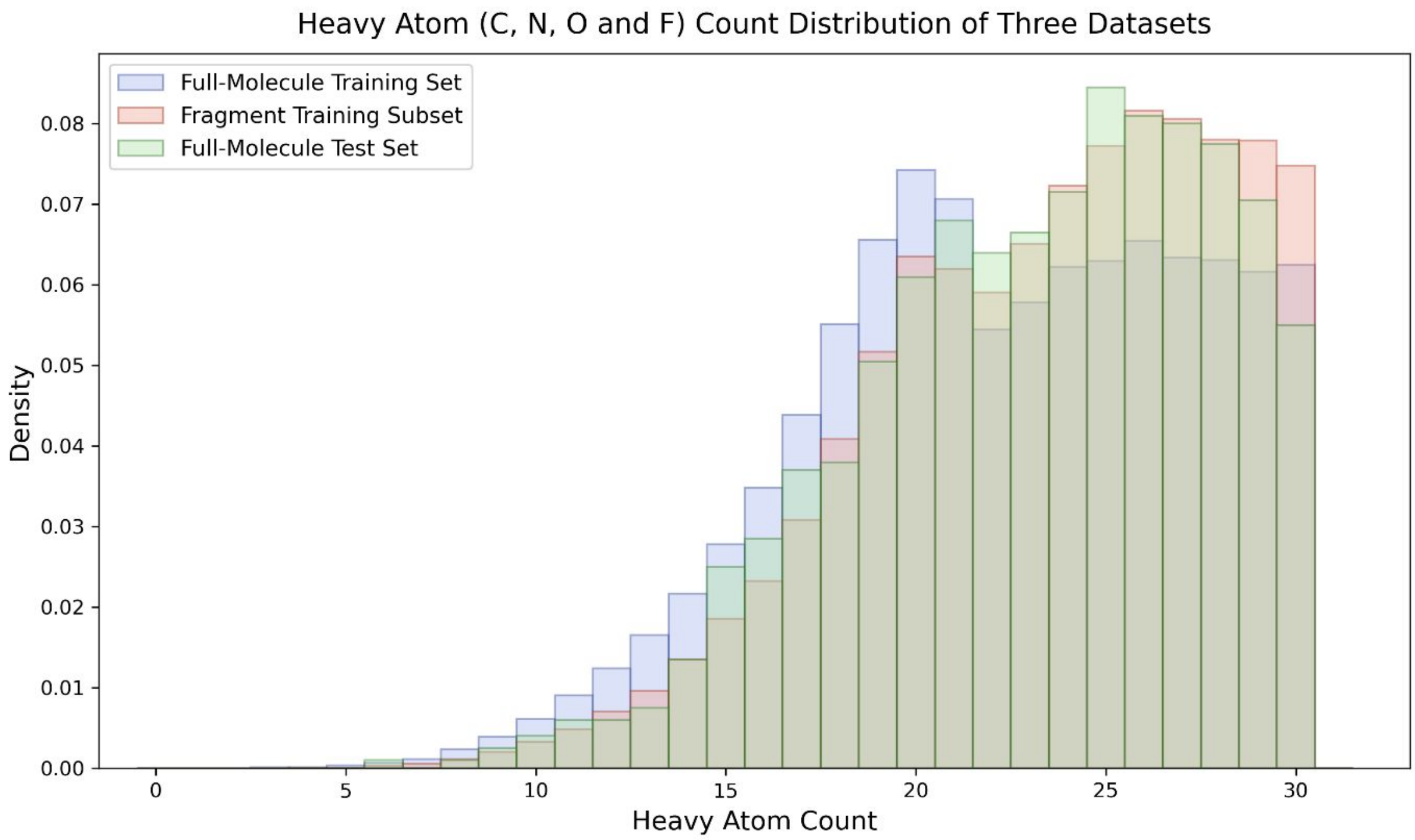
Heavy Atom (C, N, O and F) Count Distribution of Three Datasets
Full-Molecule Training Set
Fragment Training Subset
Full-Molecule Test Set
Density
Heavy Atom Count
0.00
0.01
0.02
0.03
0.04
0.05
0.06
0.07
0.08
0
5
10
15
20
25
30

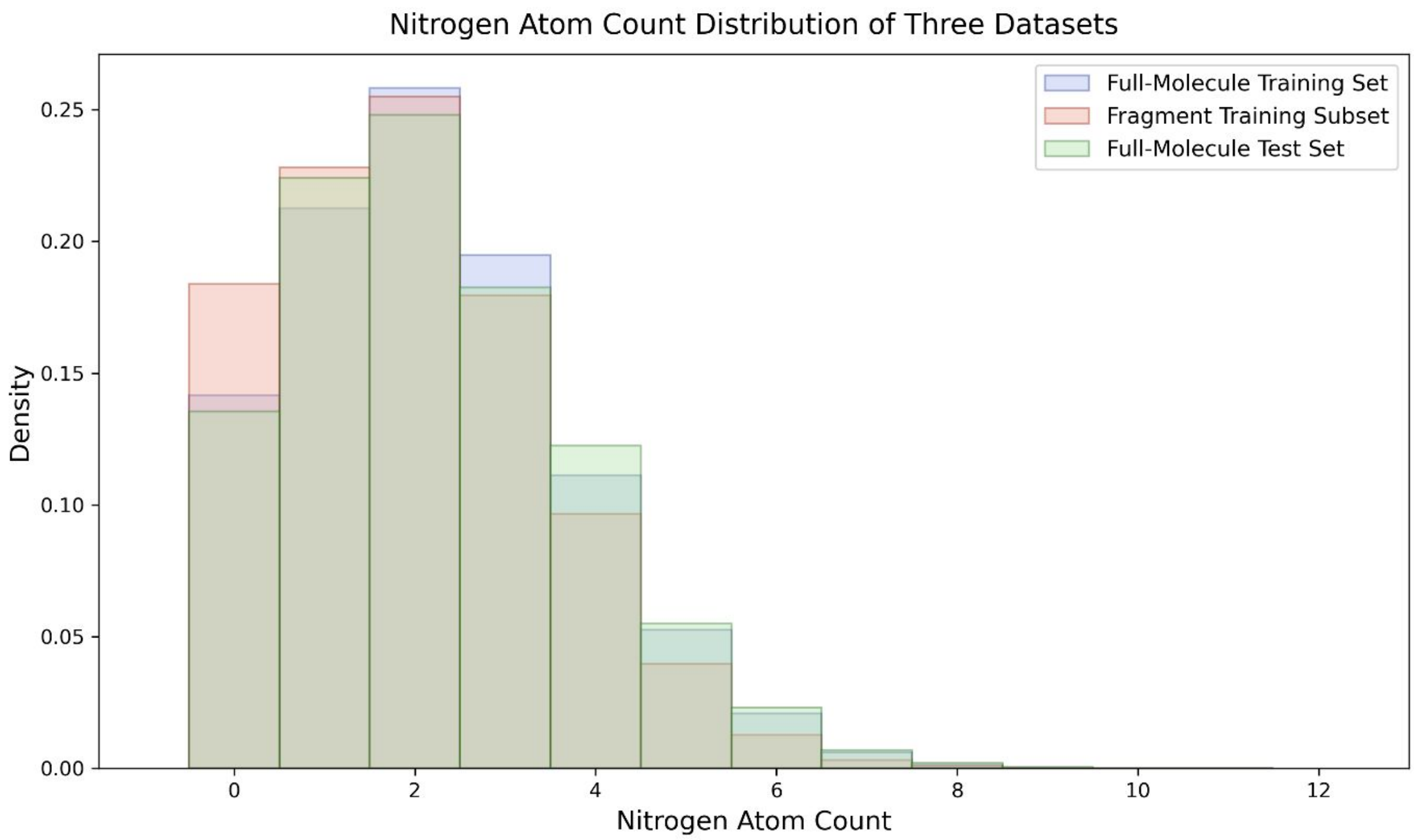
Nitrogen Atom Count Distribution of Three Datasets
Full-Molecule Training Set
Fragment Training Subset
Full-Molecule Test Set
Density
Nitrogen Atom Count
0.00
0.05
0.10
0.15
0.20
0.25
0
2
4
6
8
10
12

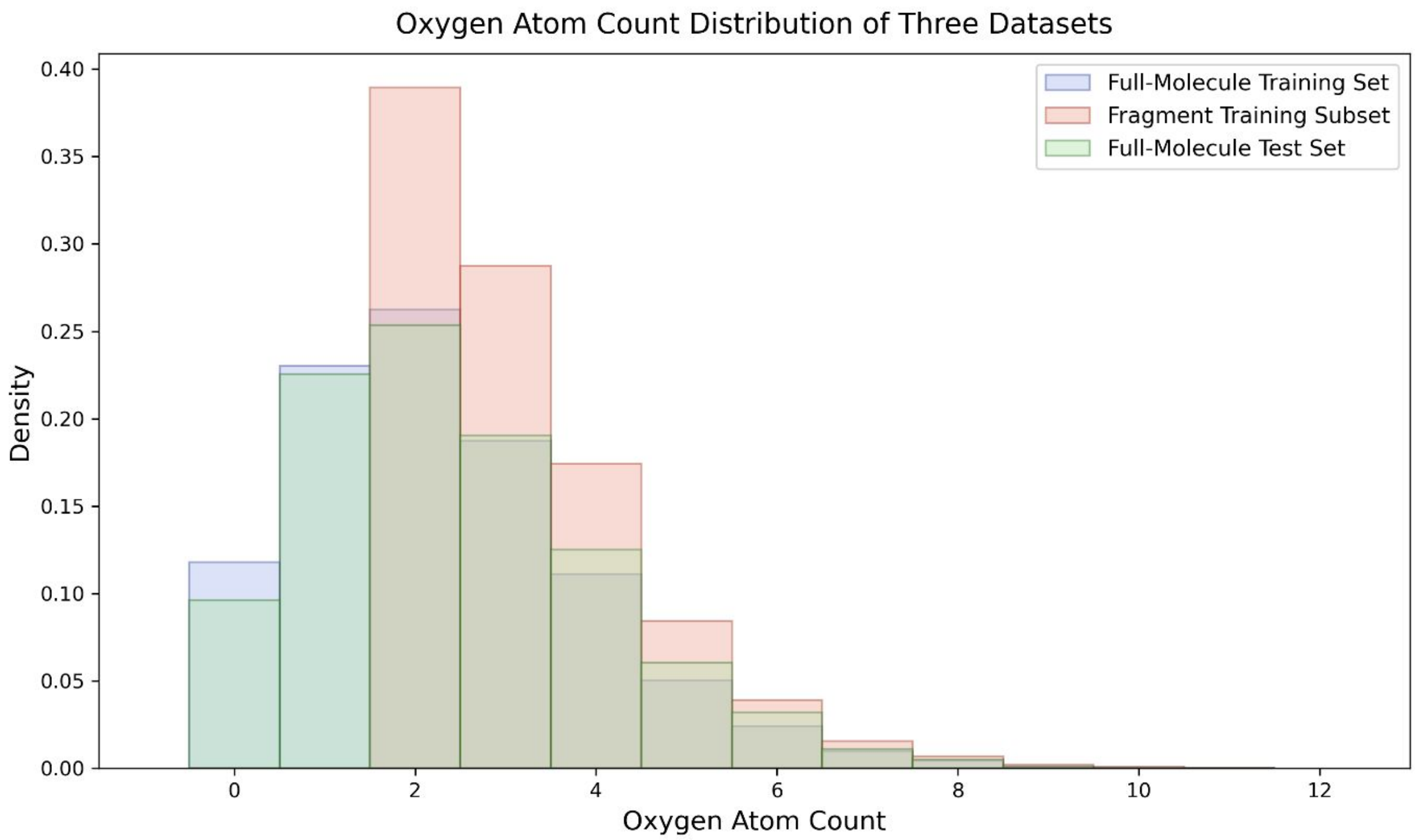
Oxygen Atom Count Distribution of Three Datasets
Full-Molecule Training Set
Fragment Training Subset
Full-Molecule Test Set
Density
0.00
0.05
0.10
0.15
0.20
0.25
0.30
0.35
0.40
0
2
4
6
8
10
12
Oxygen Atom Count

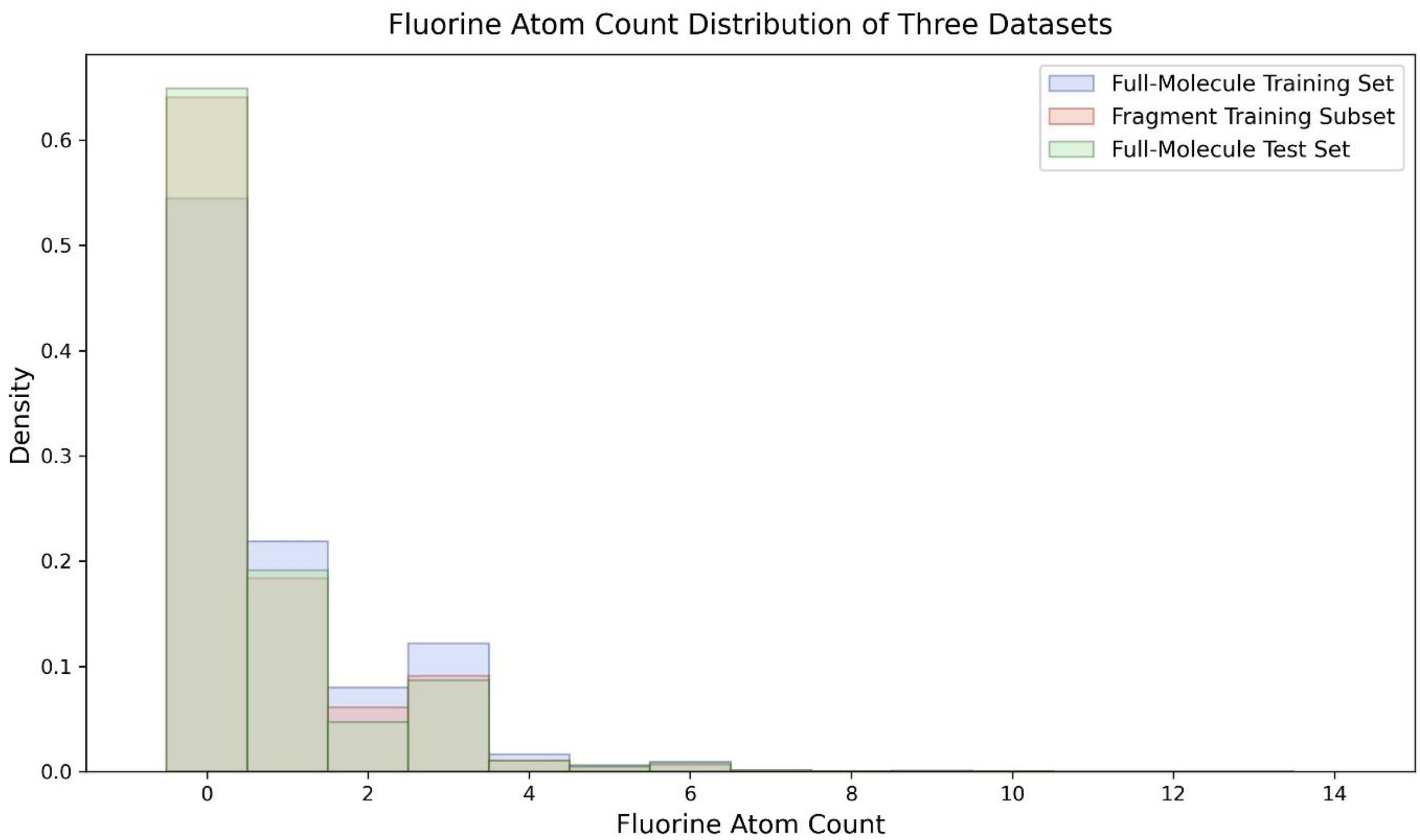
Fluorine Atom Count Distribution of Three Datasets
Full-Molecule Training Set
Fragment Training Subset
Full-Molecule Test Set
Density
0.0
0.1
0.2
0.3
0.4
0.5
0.6
0
2
4
6
8
10
12
14
Fluorine Atom Count

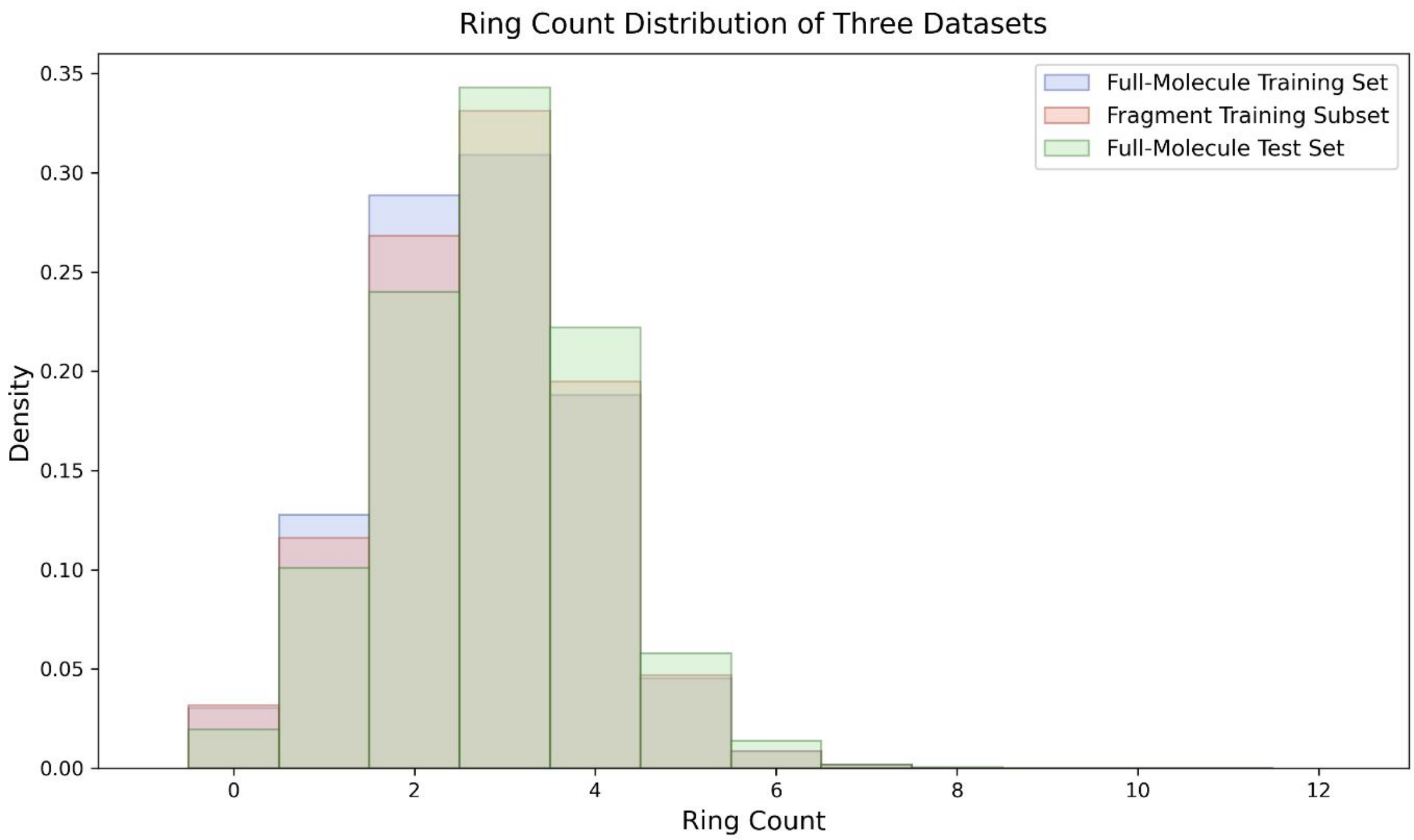
Ring Count Distribution of Three Datasets
Full-Molecule Training Set
Fragment Training Subset
Full-Molecule Test Set
Density
Ring Count
0.35
0.30
0.25
0.20
0.15
0.10
0.05
0.00
0
2
4
6
8
10
12

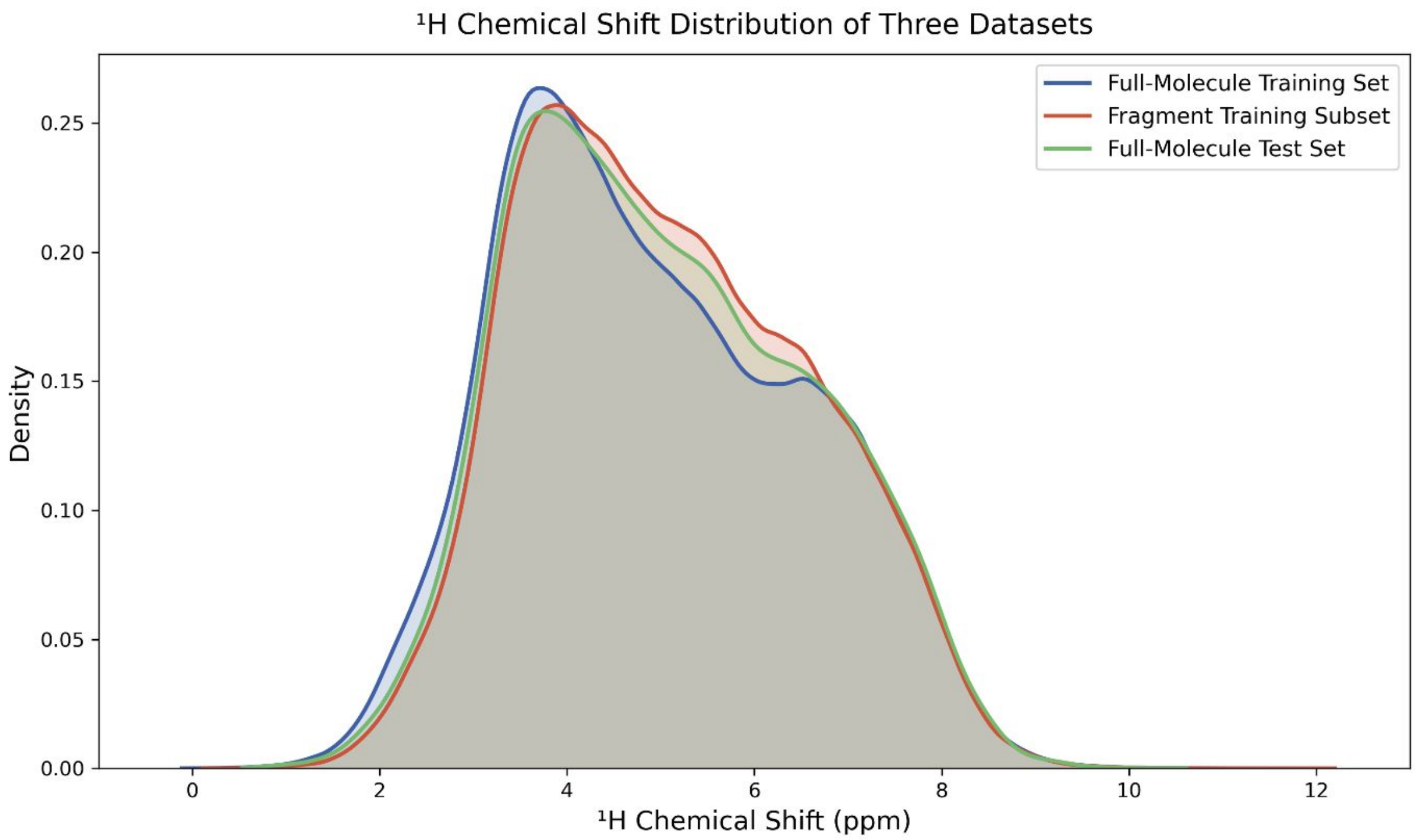
¹H Chemical Shift Distribution of Three Datasets
Full-Molecule Training Set
Fragment Training Subset
Full-Molecule Test Set
Density
¹H Chemical Shift (ppm)
0.25
0.20
0.15
0.10
0.05
0.00
0
2
4
6
8
10
12

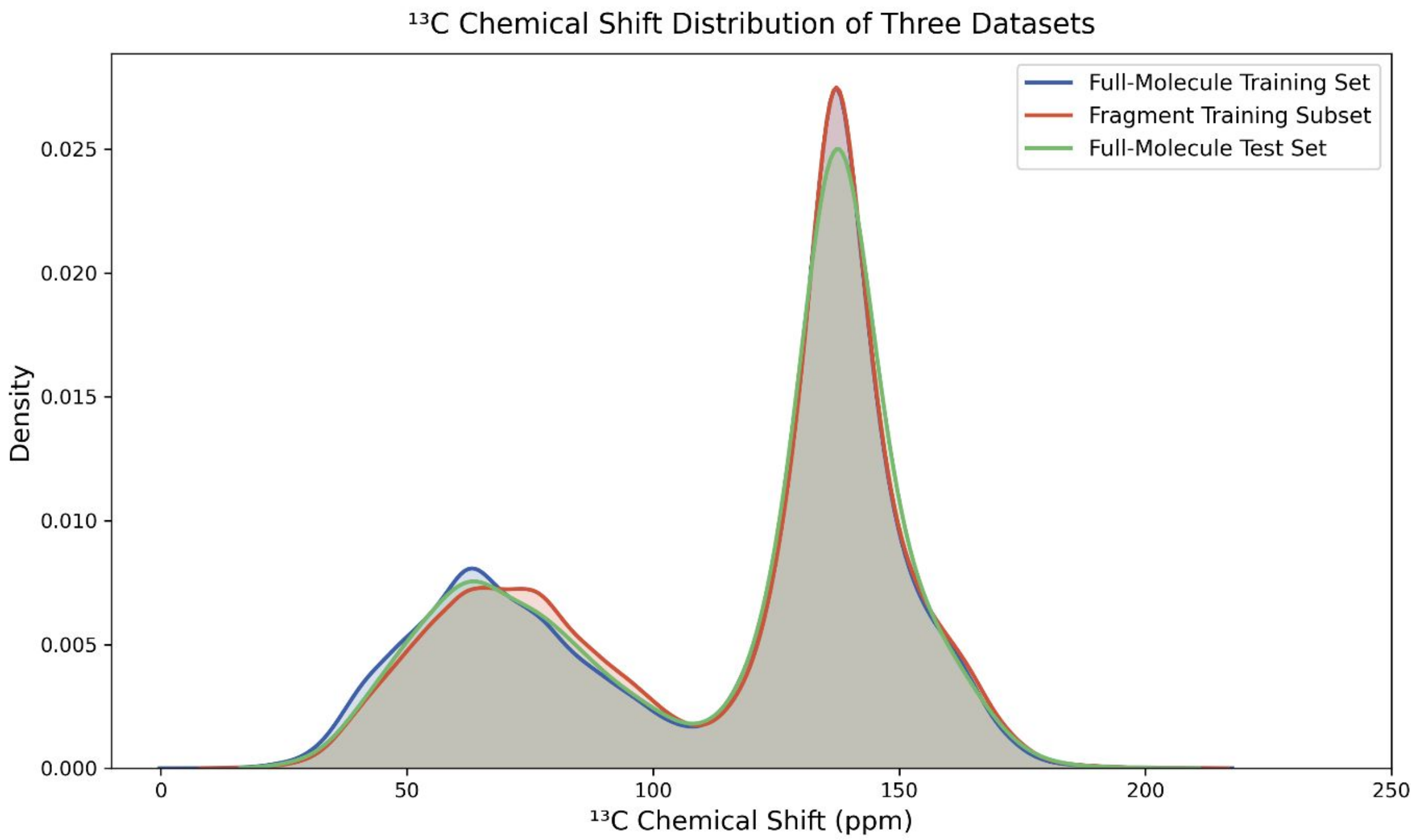
¹³C Chemical Shift Distribution of Three Datasets
Full-Molecule Training Set
Fragment Training Subset
Full-Molecule Test Set
Density
0.000
0.005
0.010
0.015
0.020
0.025
0
50
100
150
200
250
¹³C Chemical Shift (ppm)

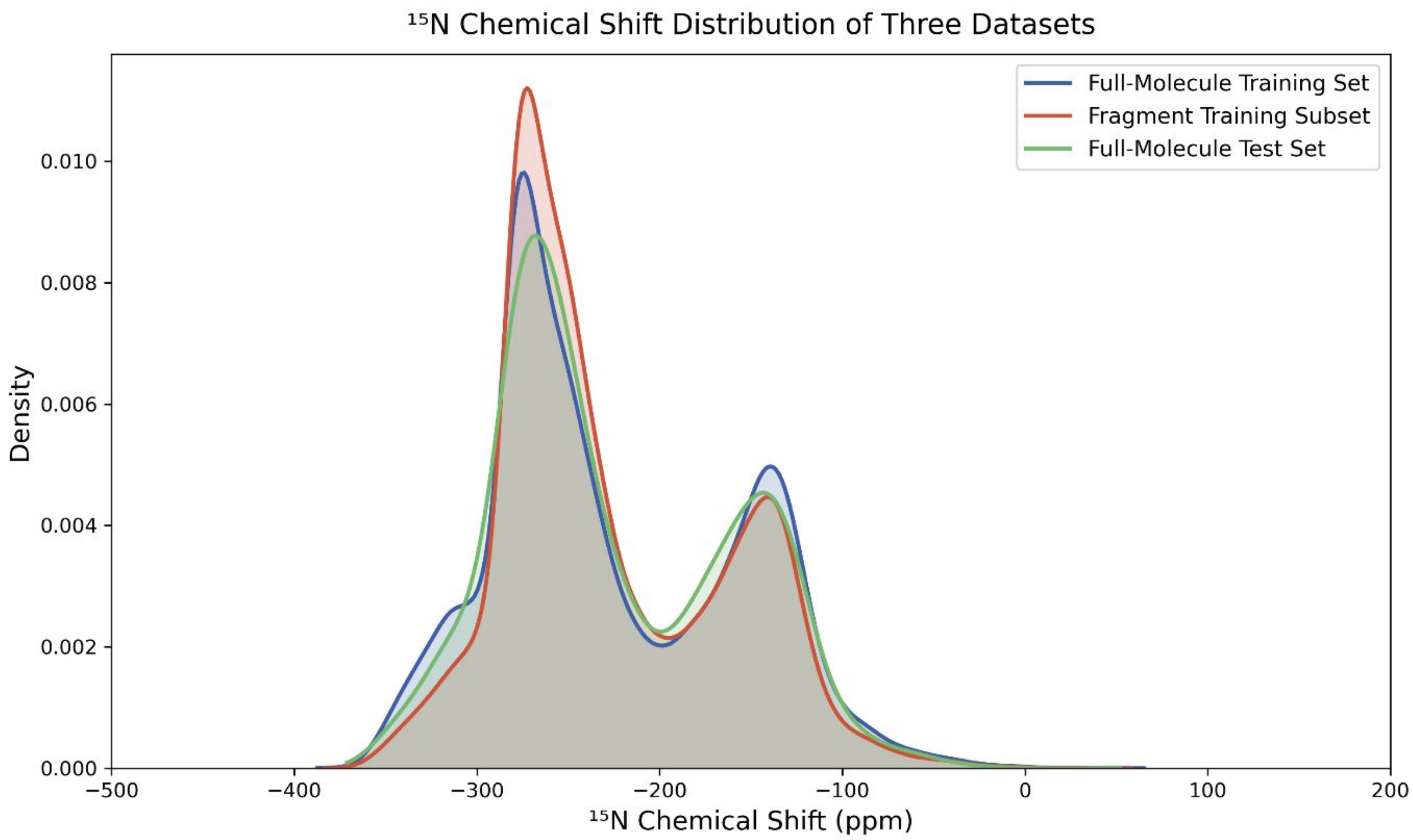
¹⁵N Chemical Shift Distribution of Three Datasets
Full-Molecule Training Set
Fragment Training Subset
Full-Molecule Test Set
Density
0.000
0.002
0.004
0.006
0.008
0.010
−500
−400
−300
−200
−100
0
100
200
¹⁵N Chemical Shift (ppm)

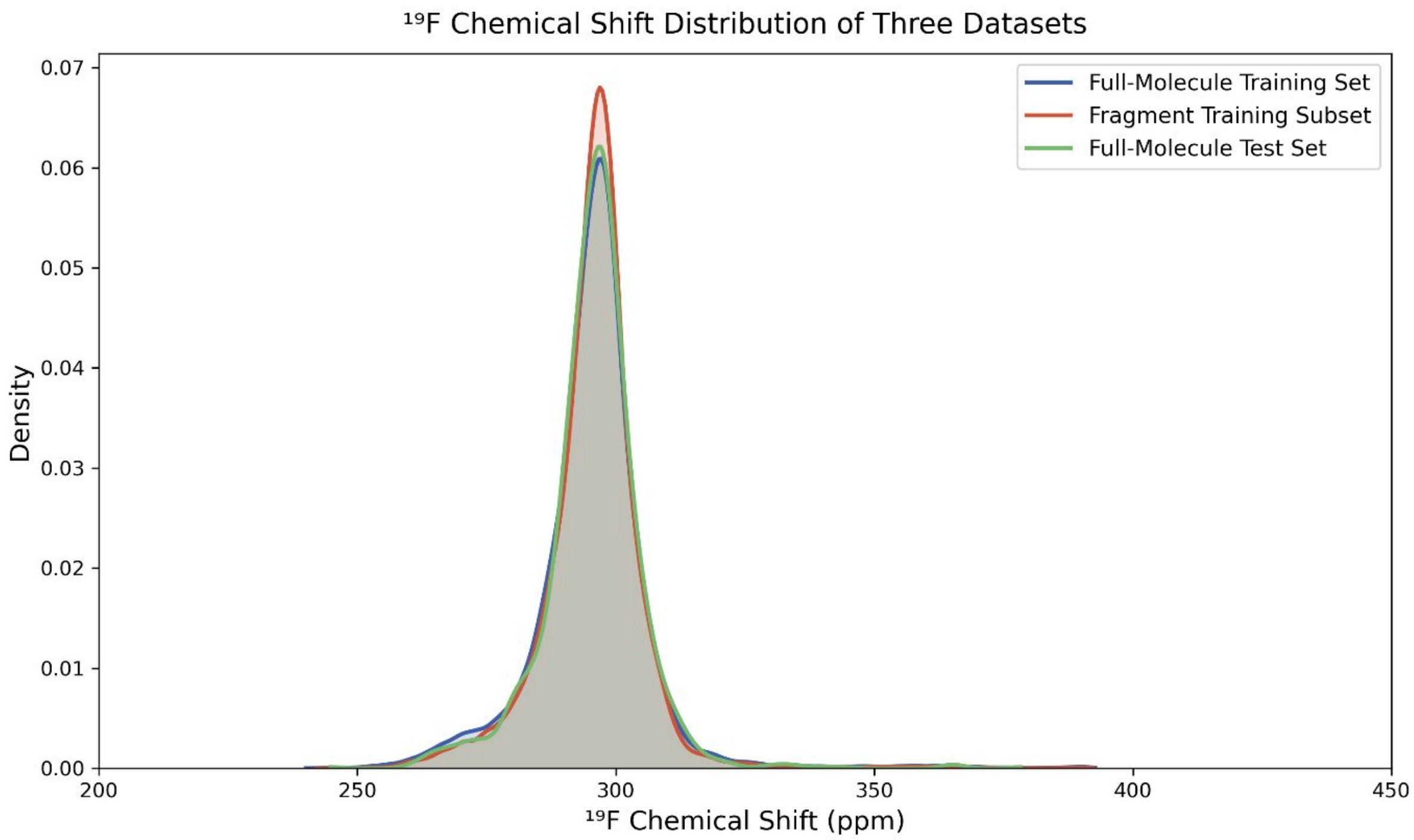
¹⁹F Chemical Shift Distribution of Three Datasets
Full-Molecule Training Set
Fragment Training Subset
Full-Molecule Test Set
Density
0.07
0.06
0.05
0.04
0.03
0.02
0.01
0.00
200
250
300
350
400
450
¹⁹F Chemical Shift (ppm)

4 Experimental NMR Spectra

4.1 1-(4-ethylphenyl)ethenone

^{1}H NMR

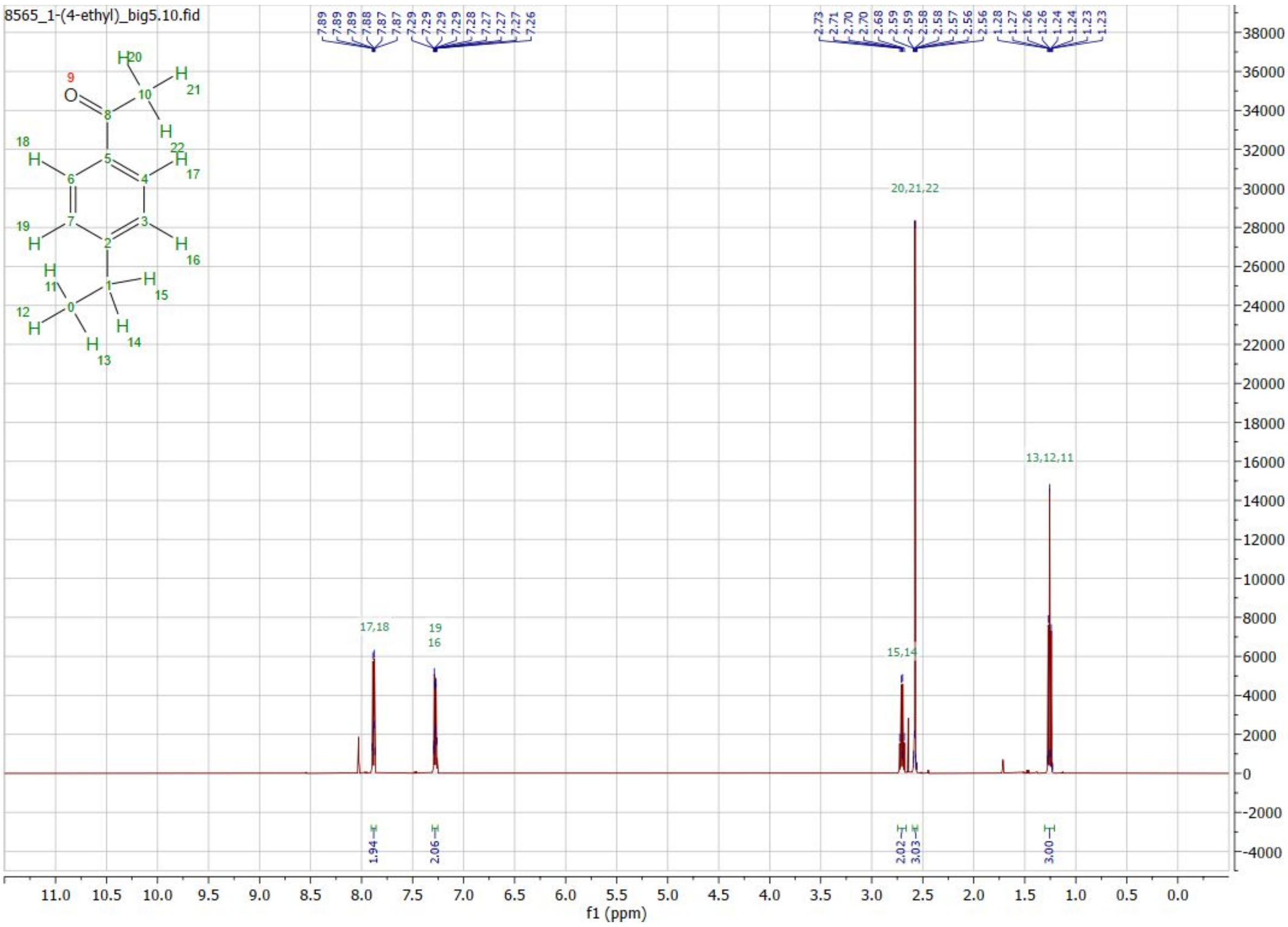

^{13}C NMR

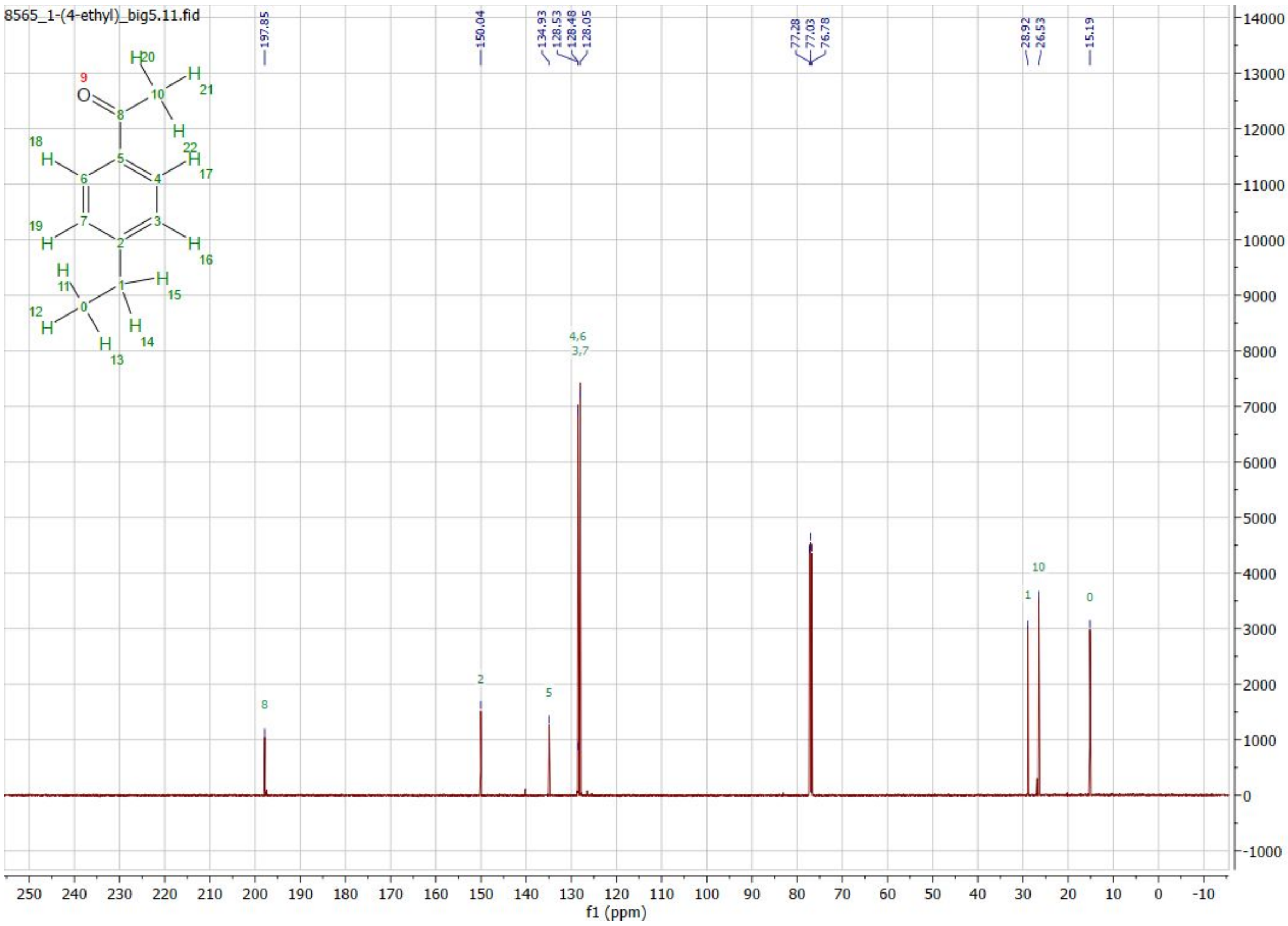

^{1}H-^{1}H COSY

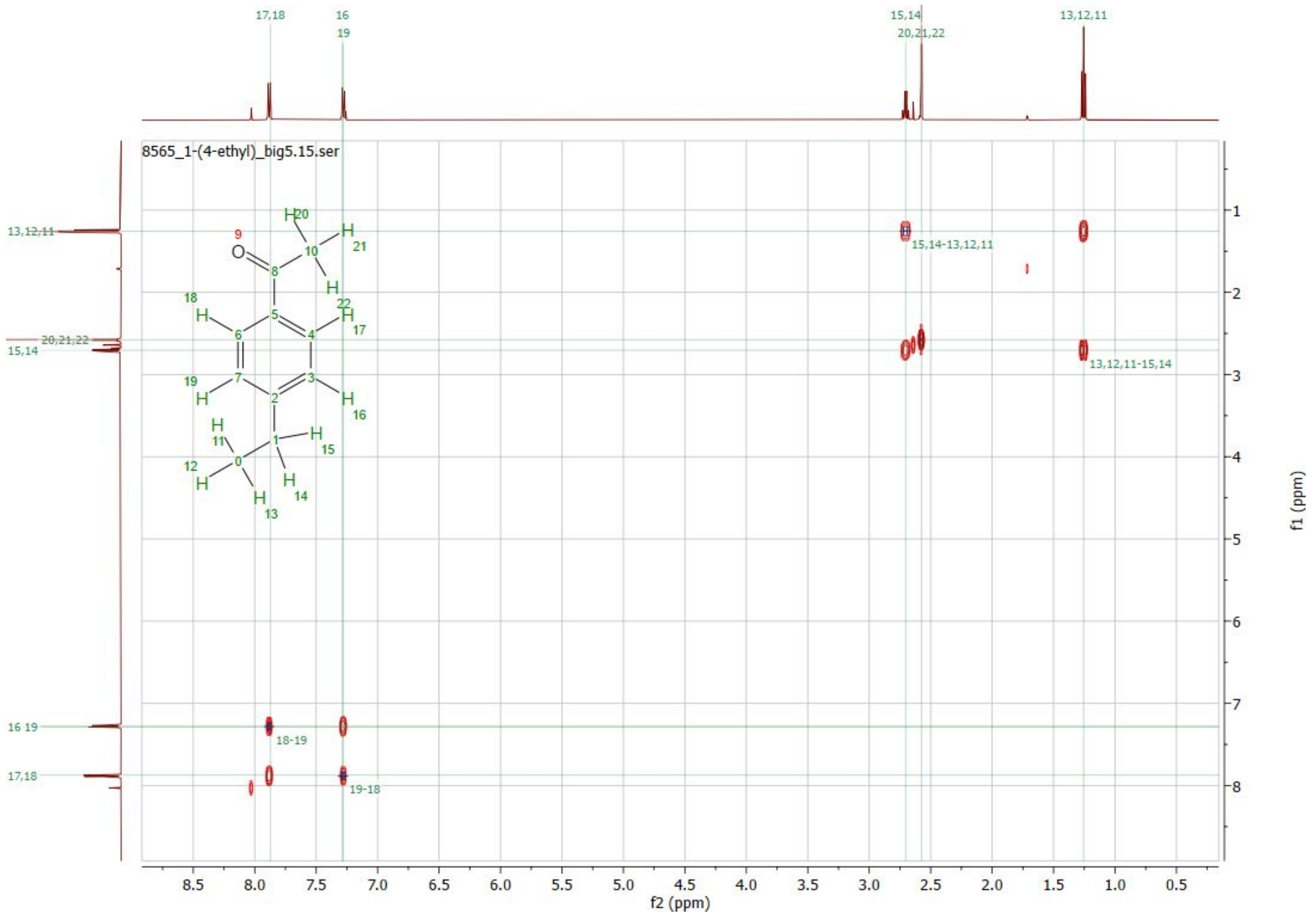

Pure shift ^1H-^{13}C HSQC

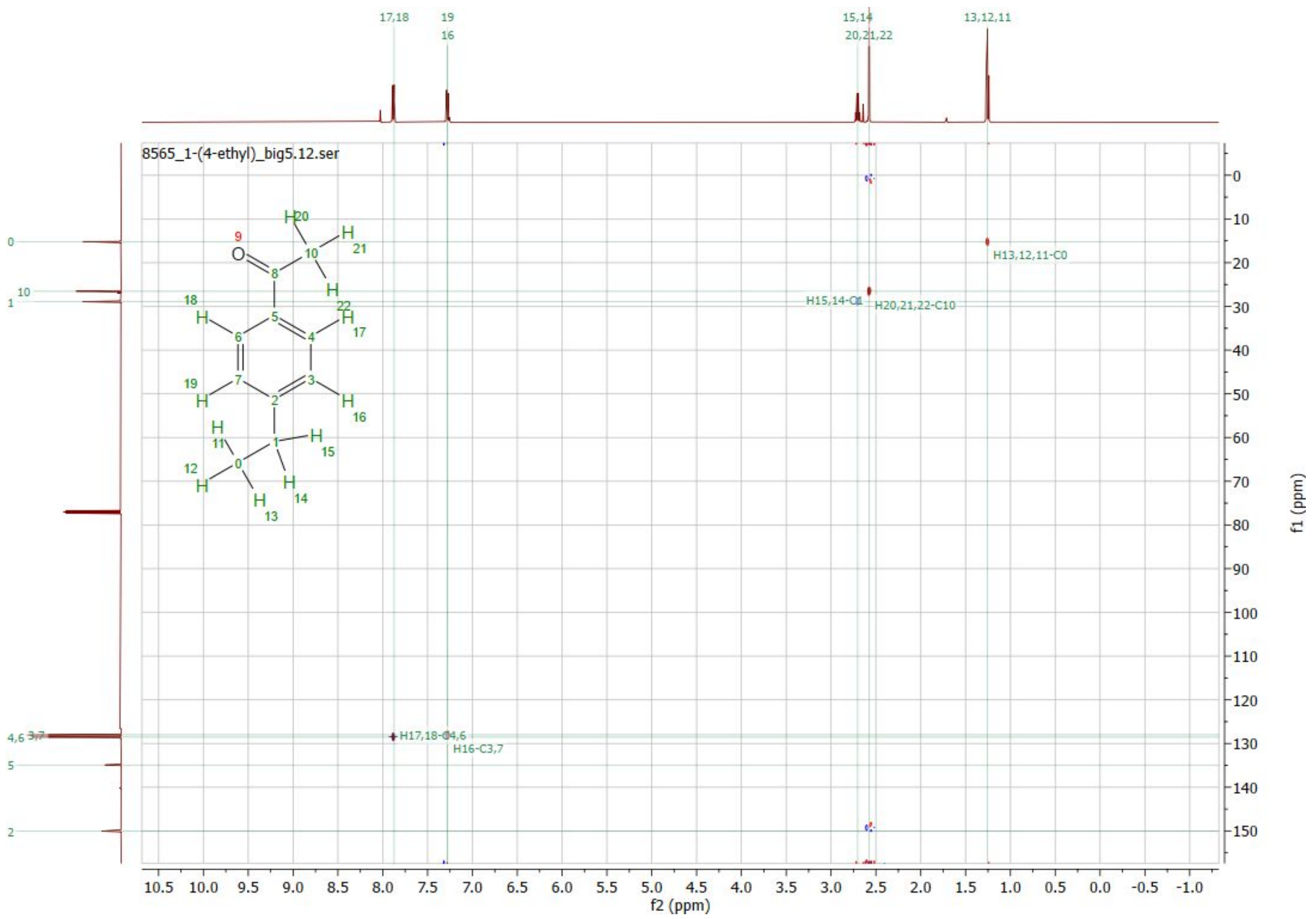

^1H-^{13}C HMBC

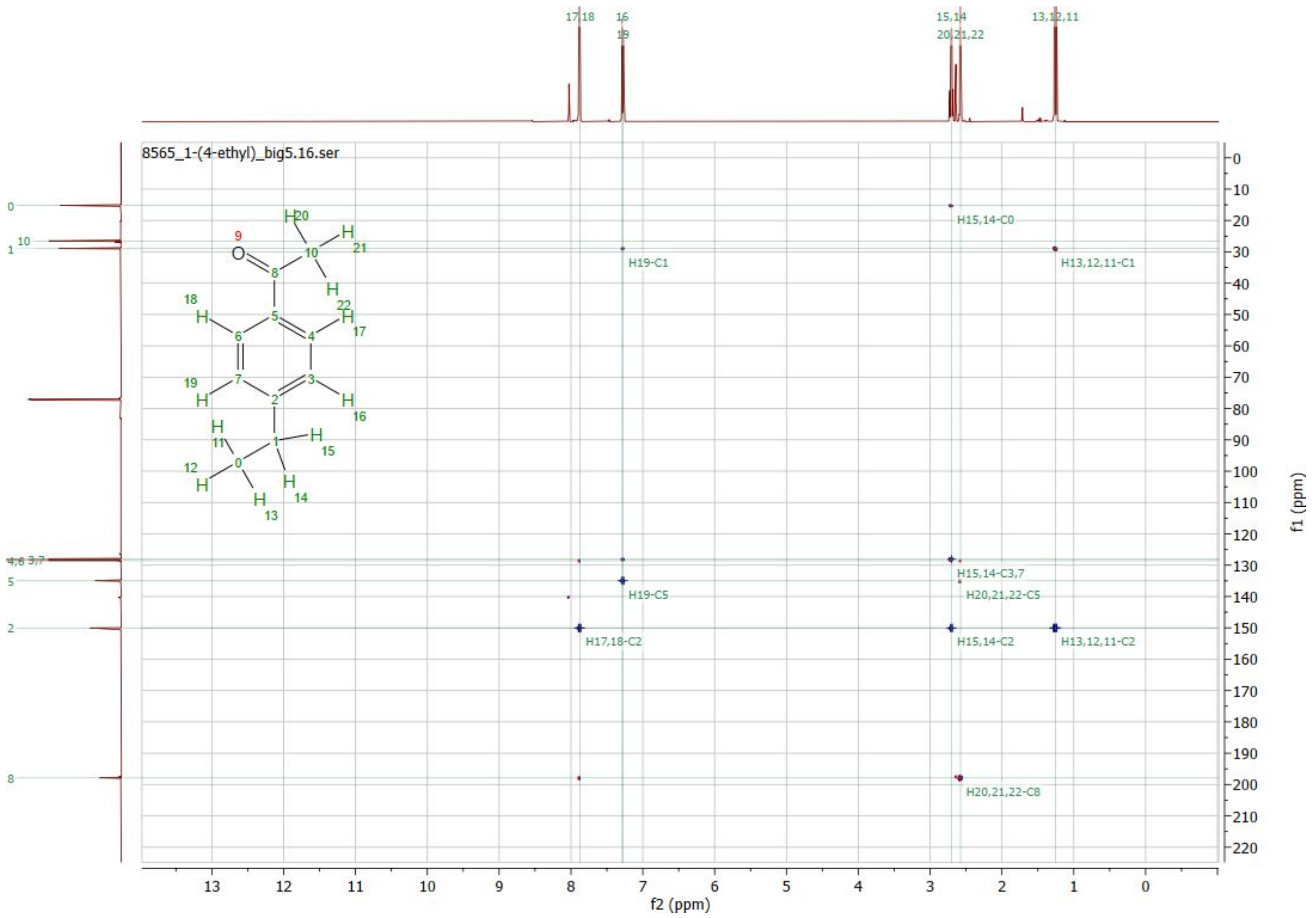

4.2 4-piperidone ethylene acetal

^{1}H NMR

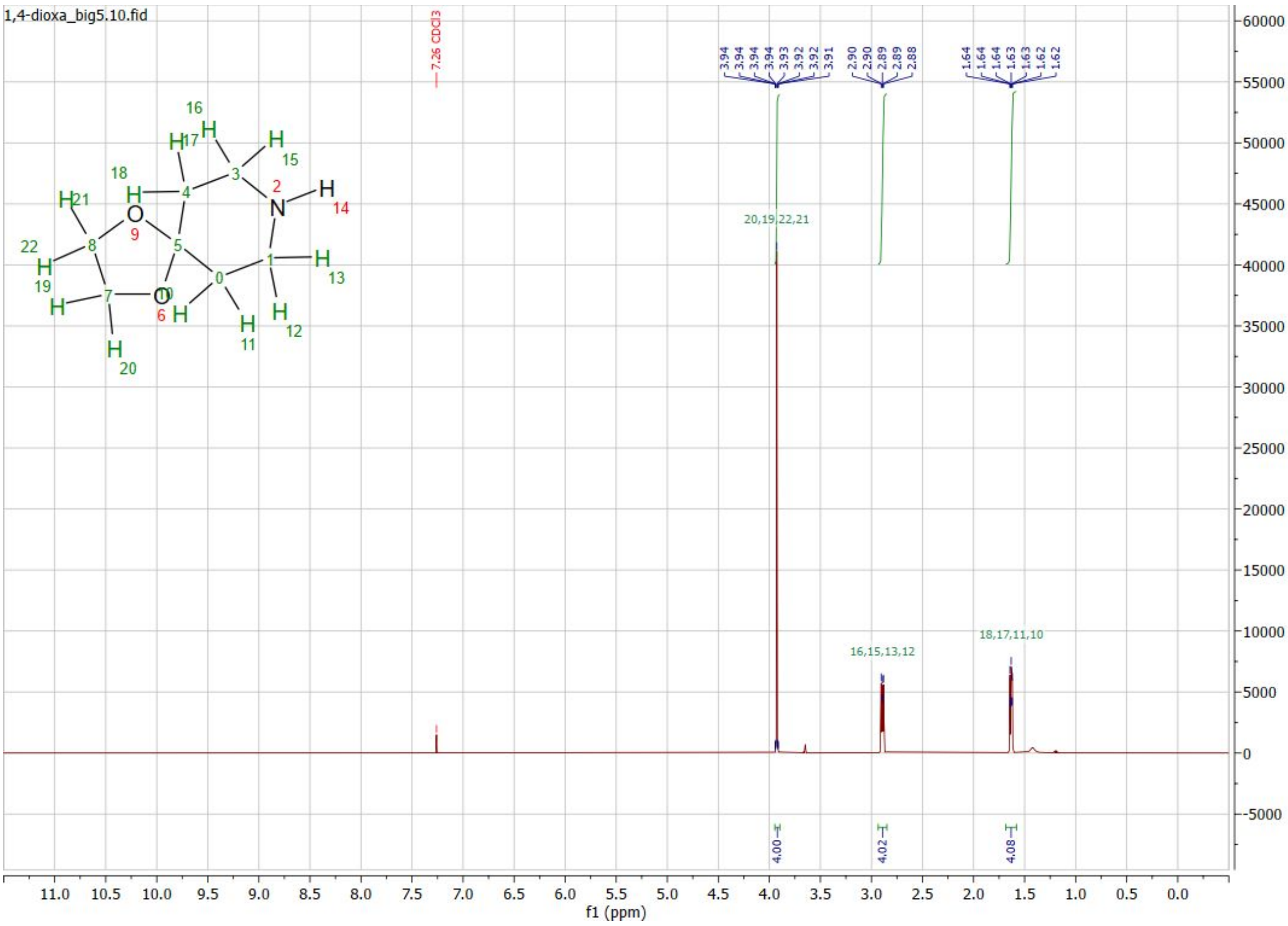

^{13}C NMR

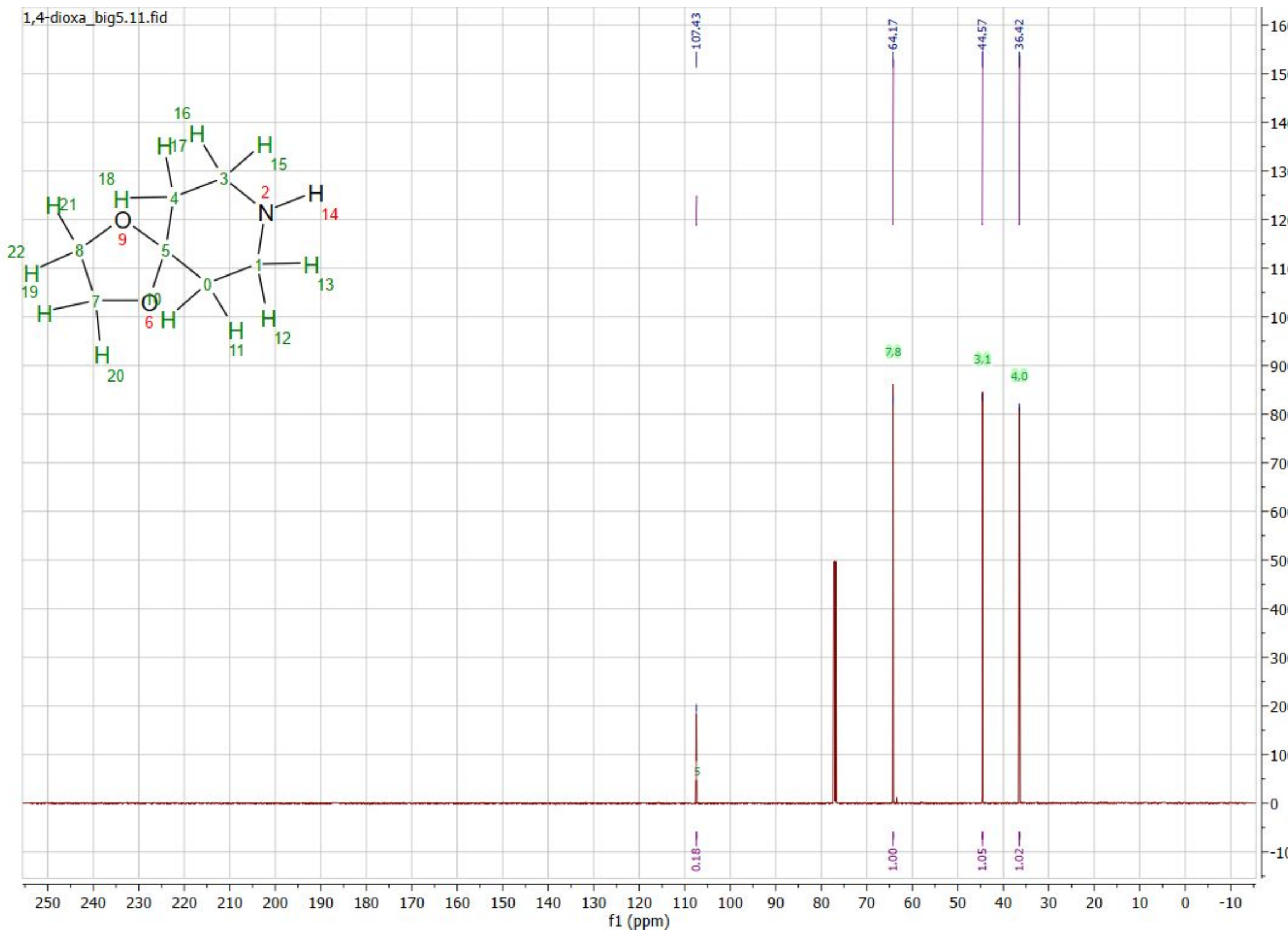

^{1}H-^{1}H COSY

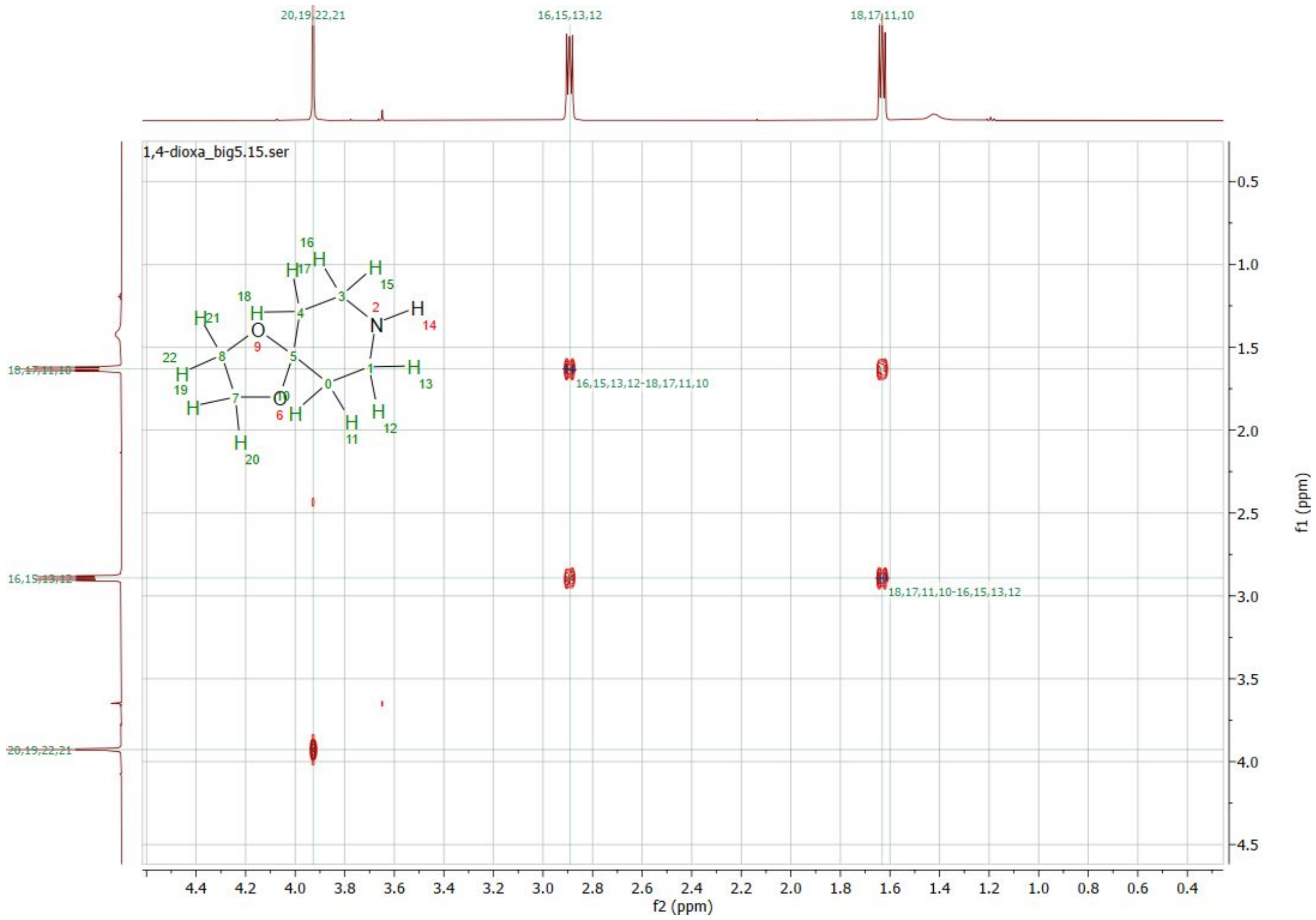

Pure shift ^{1}H-^{13}C HSQC

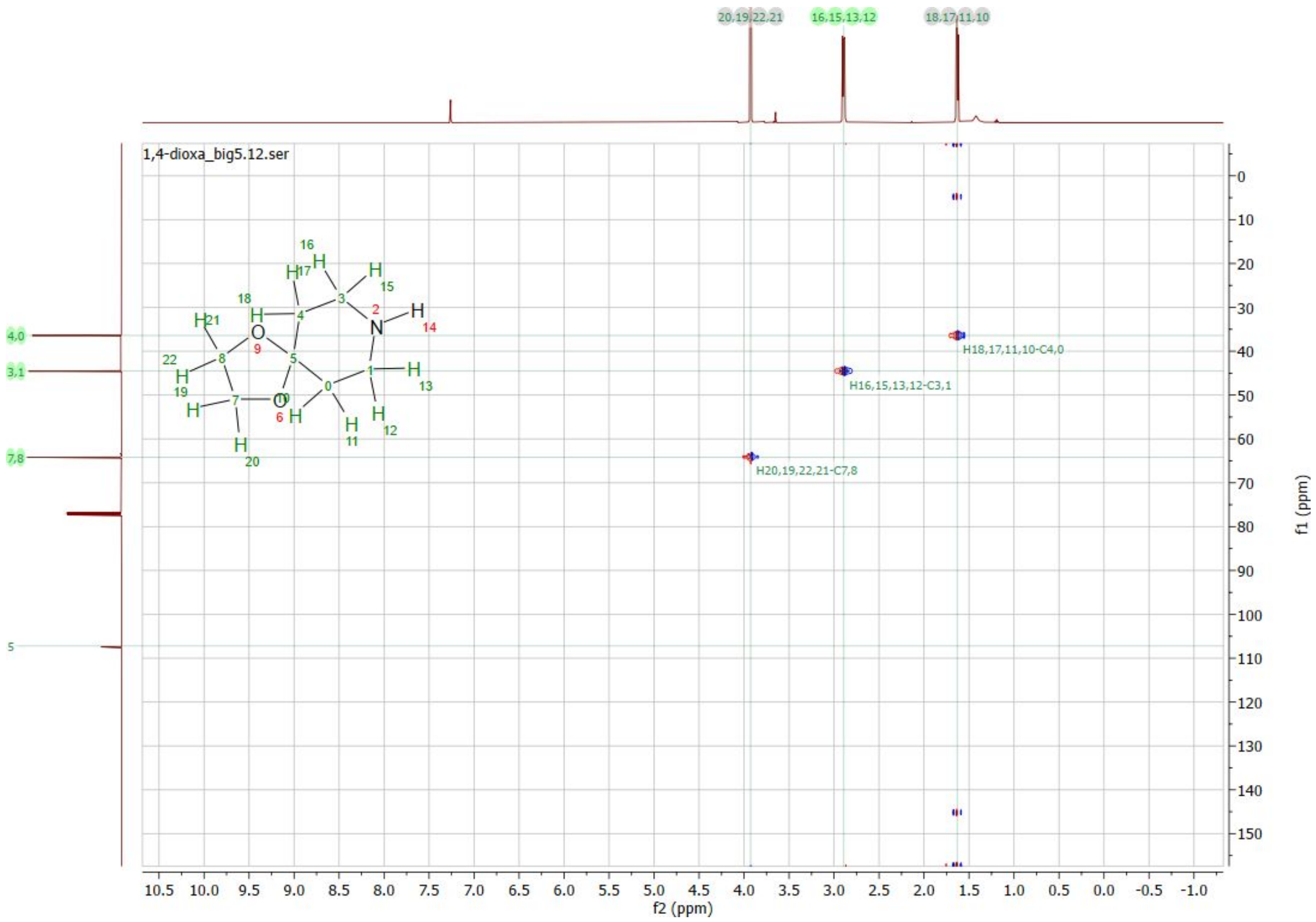

^{1}H-^{13}C HMBC

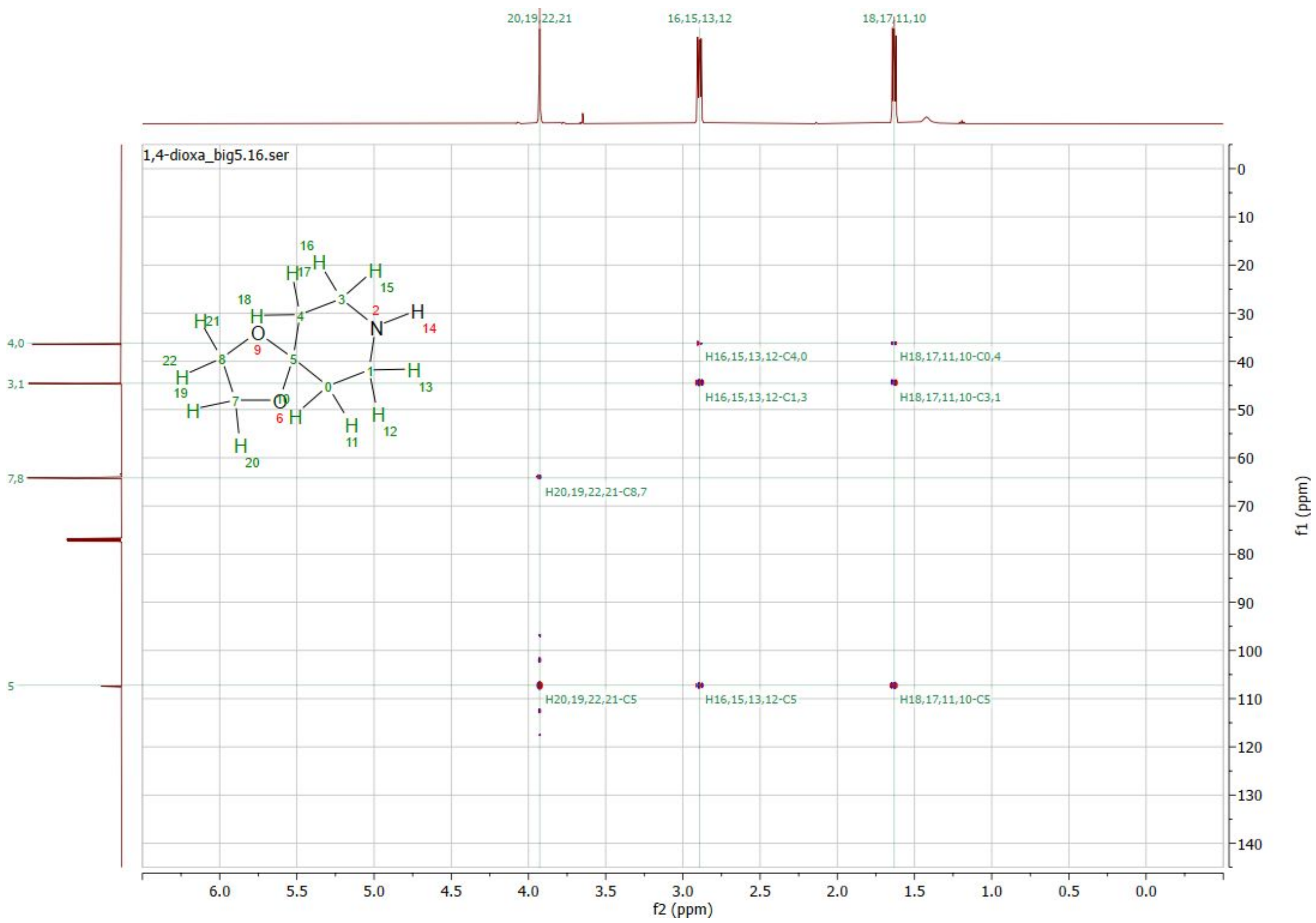

4.3 2-ethoxy-5-methylpyridin-4-amine

^{1}H NMR

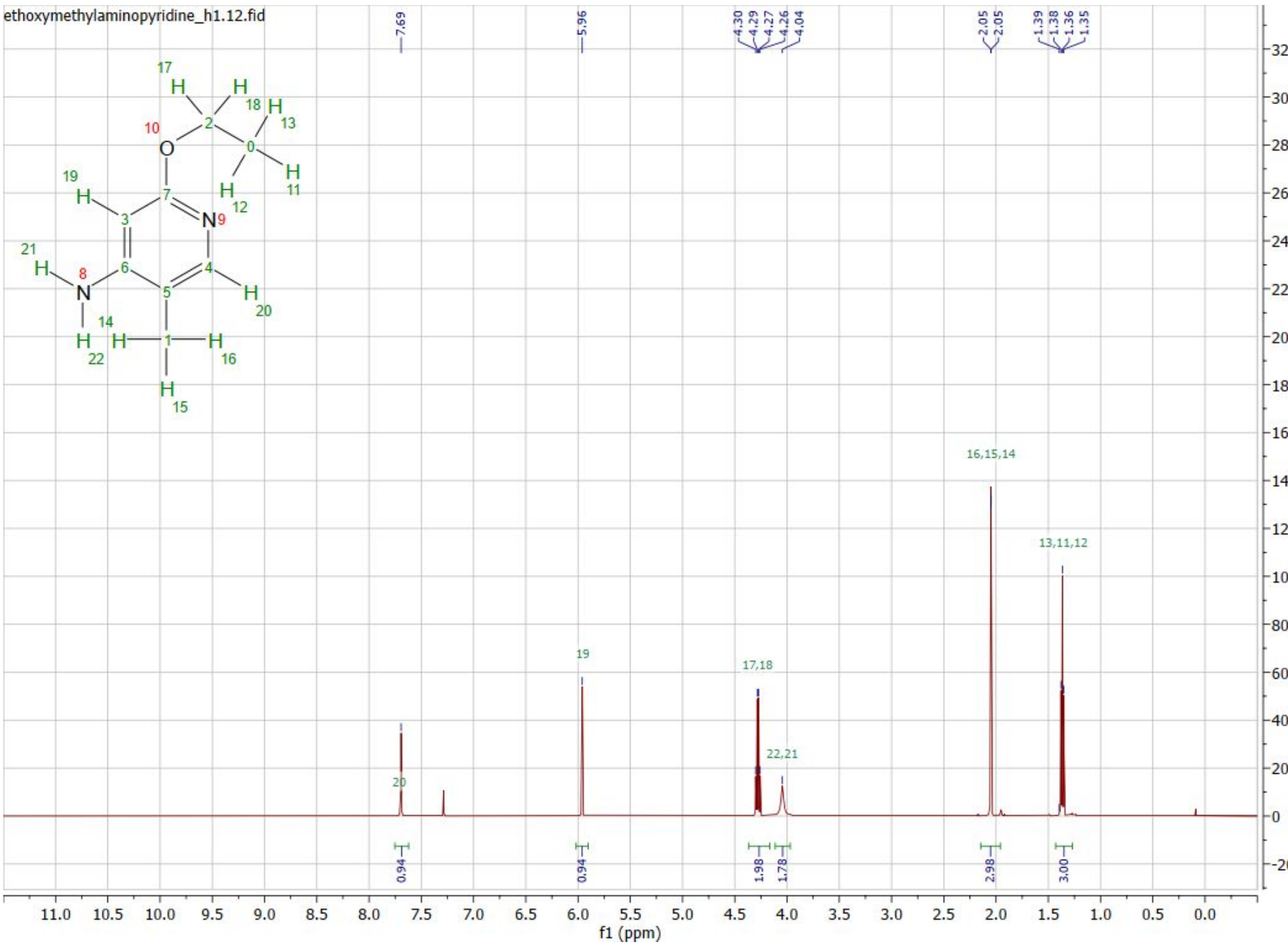

^{13}C NMR

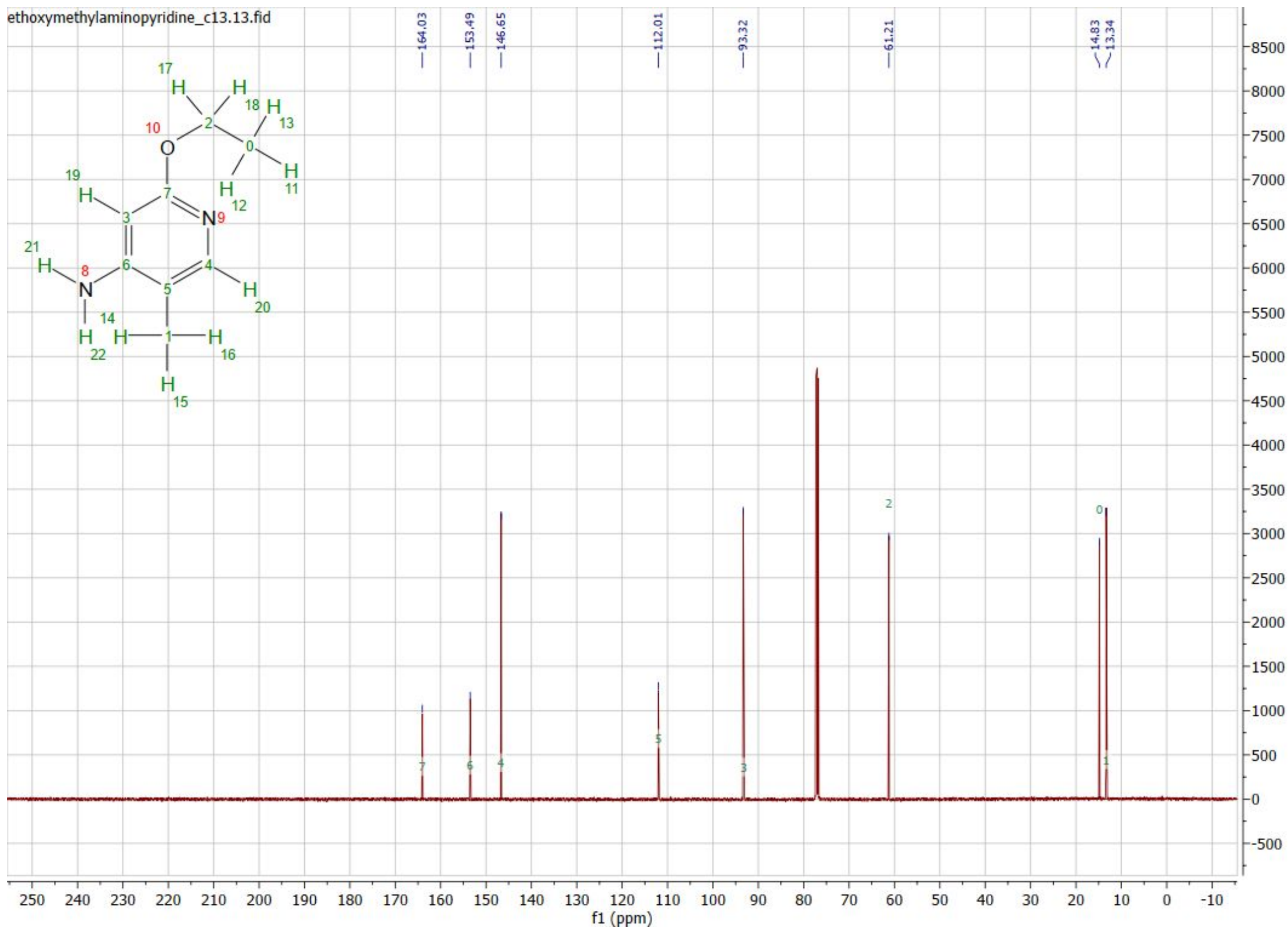

^{1}H-^{1}H COSY

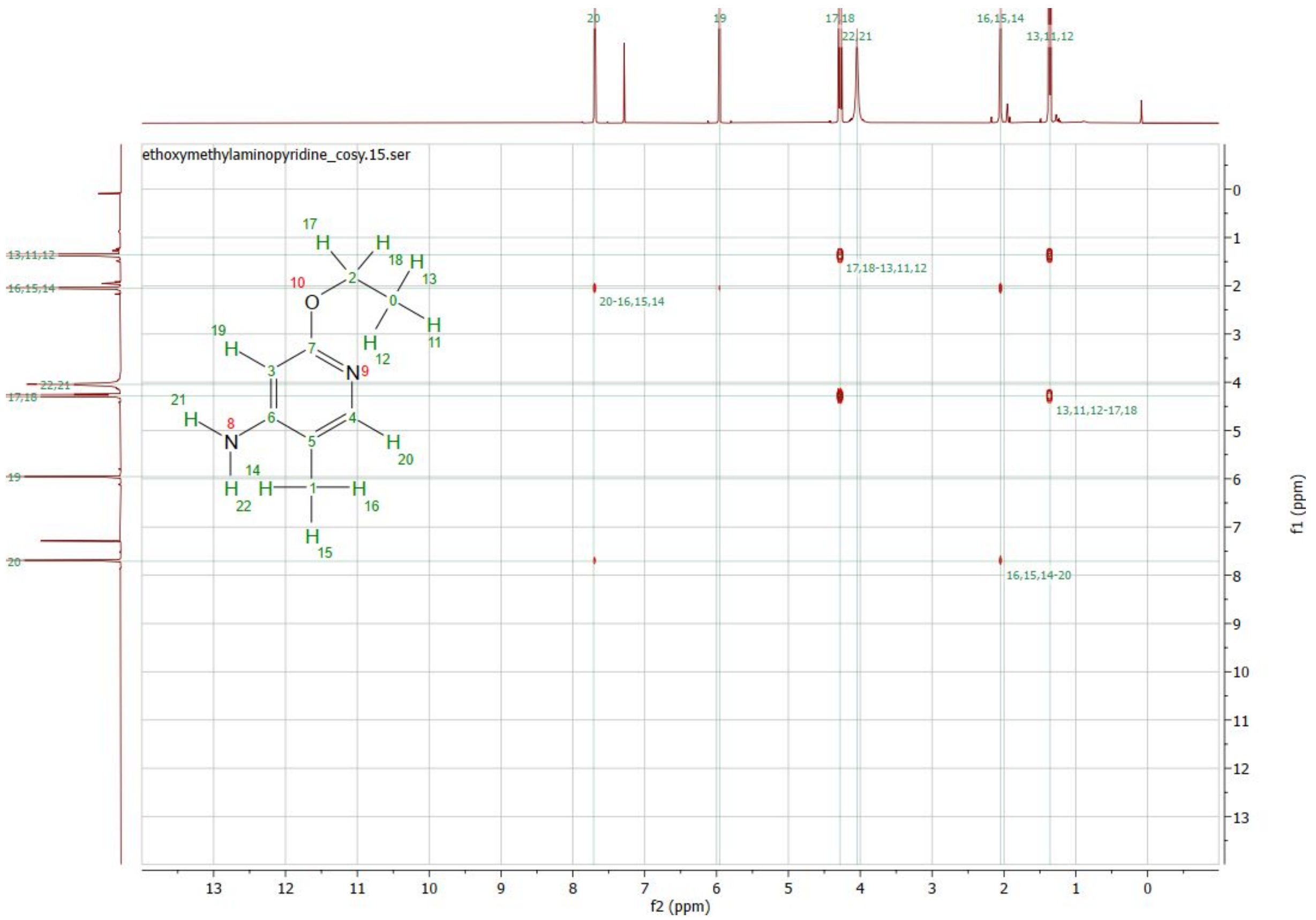

Pure shift ¹H-¹³C HSQC

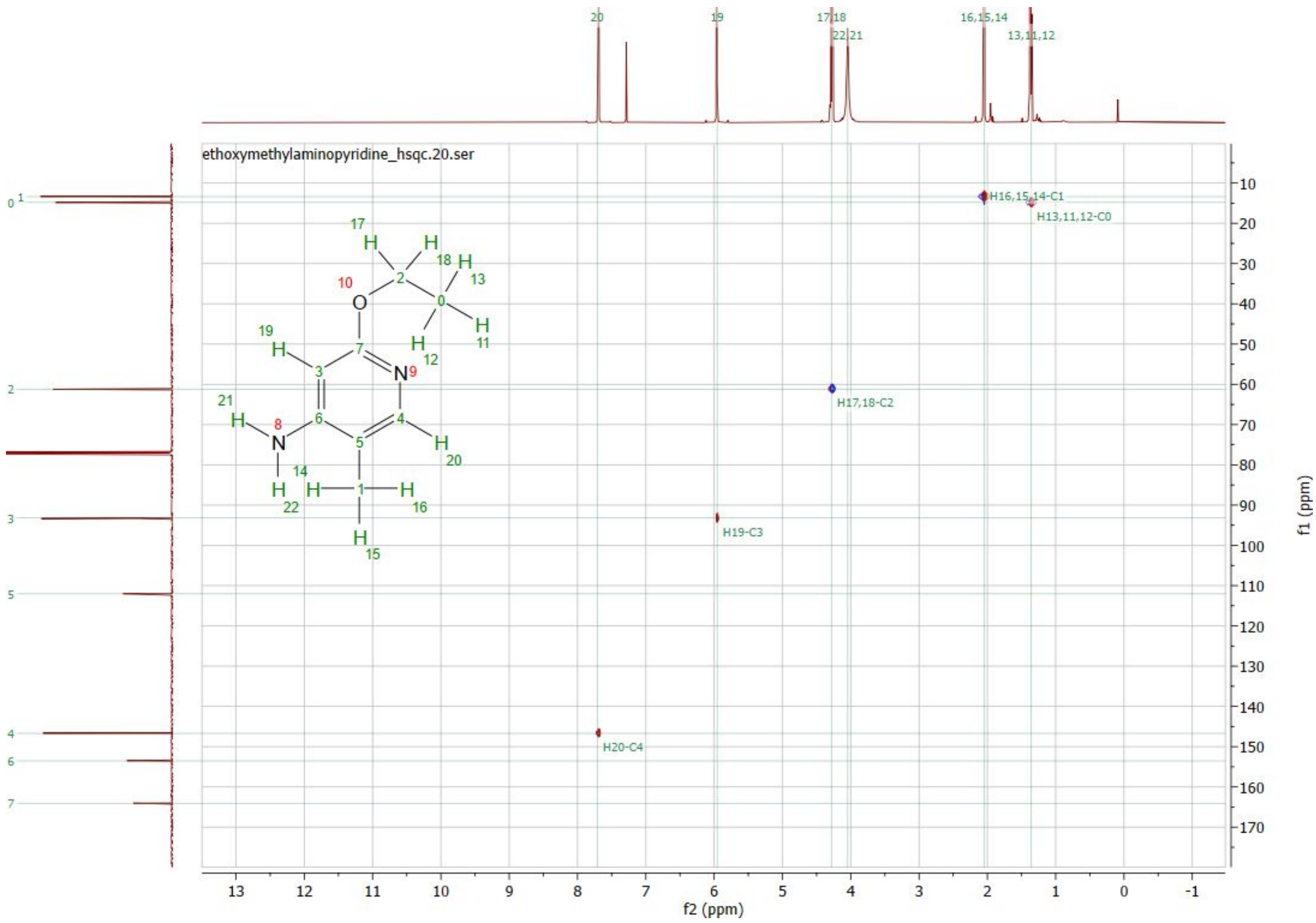

¹H-¹³C HMBC

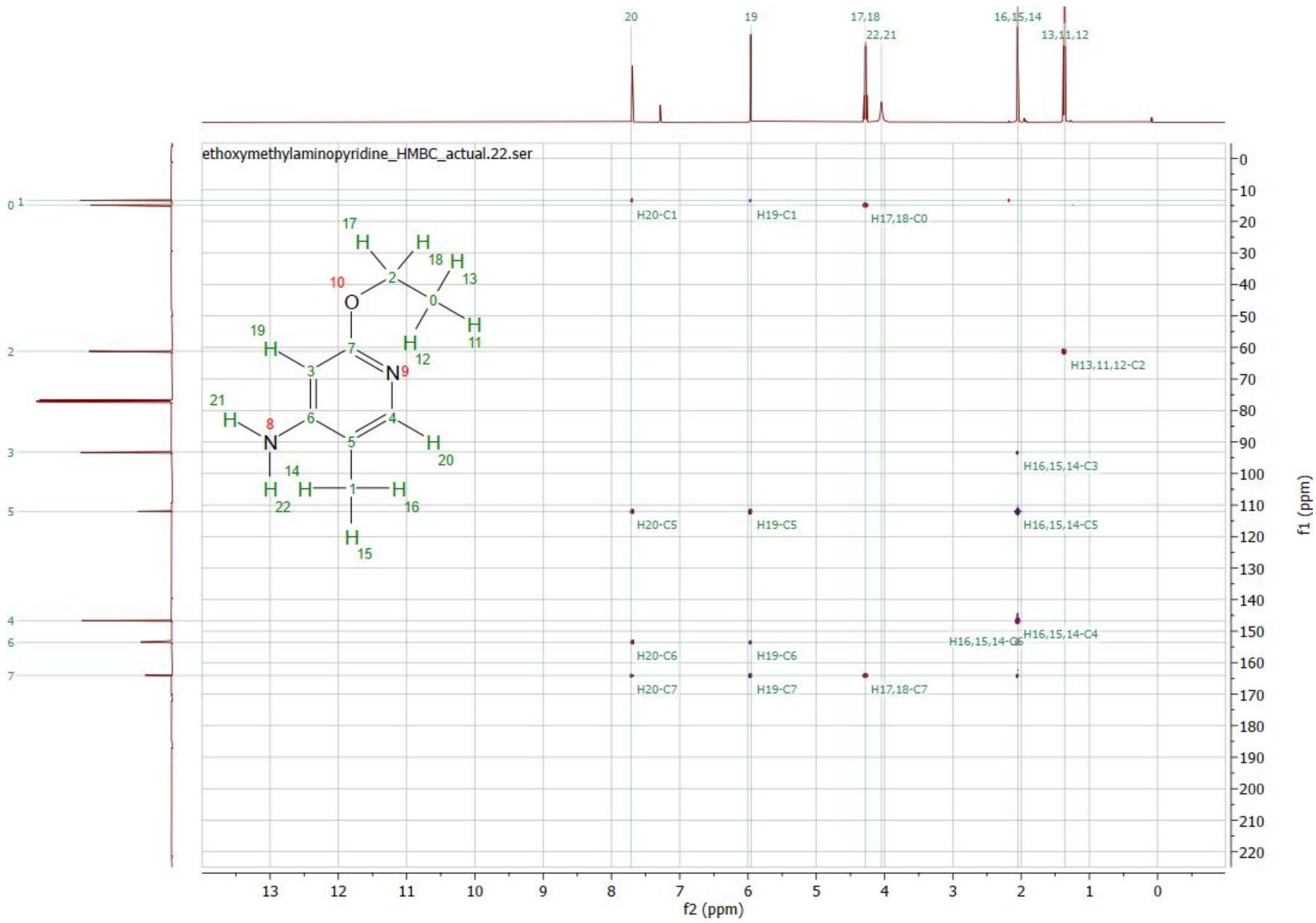

4.4 1-Boc-4-(4-aminophenyl)piperazine

^{1}H NMR

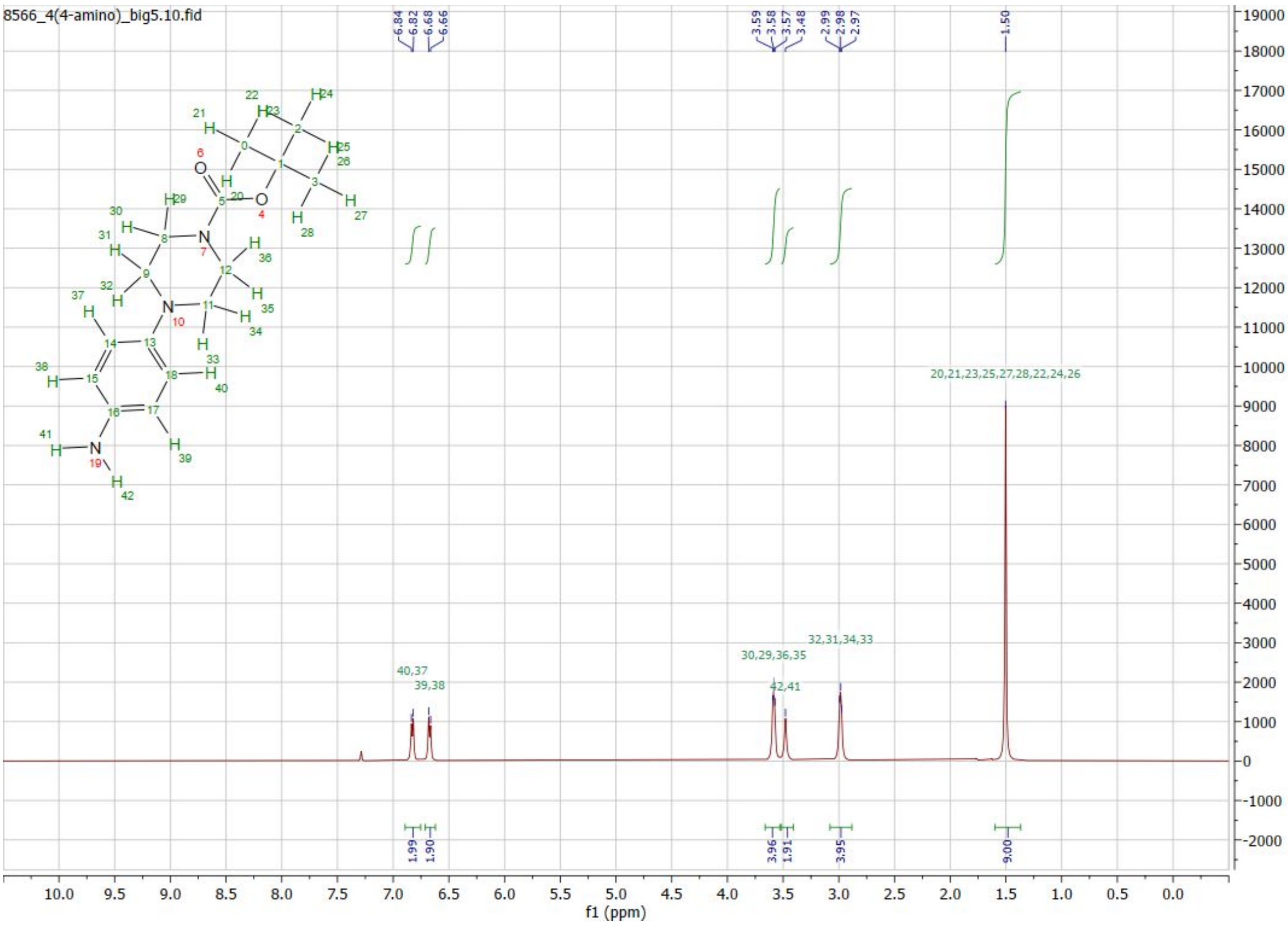

^{13}C NMR

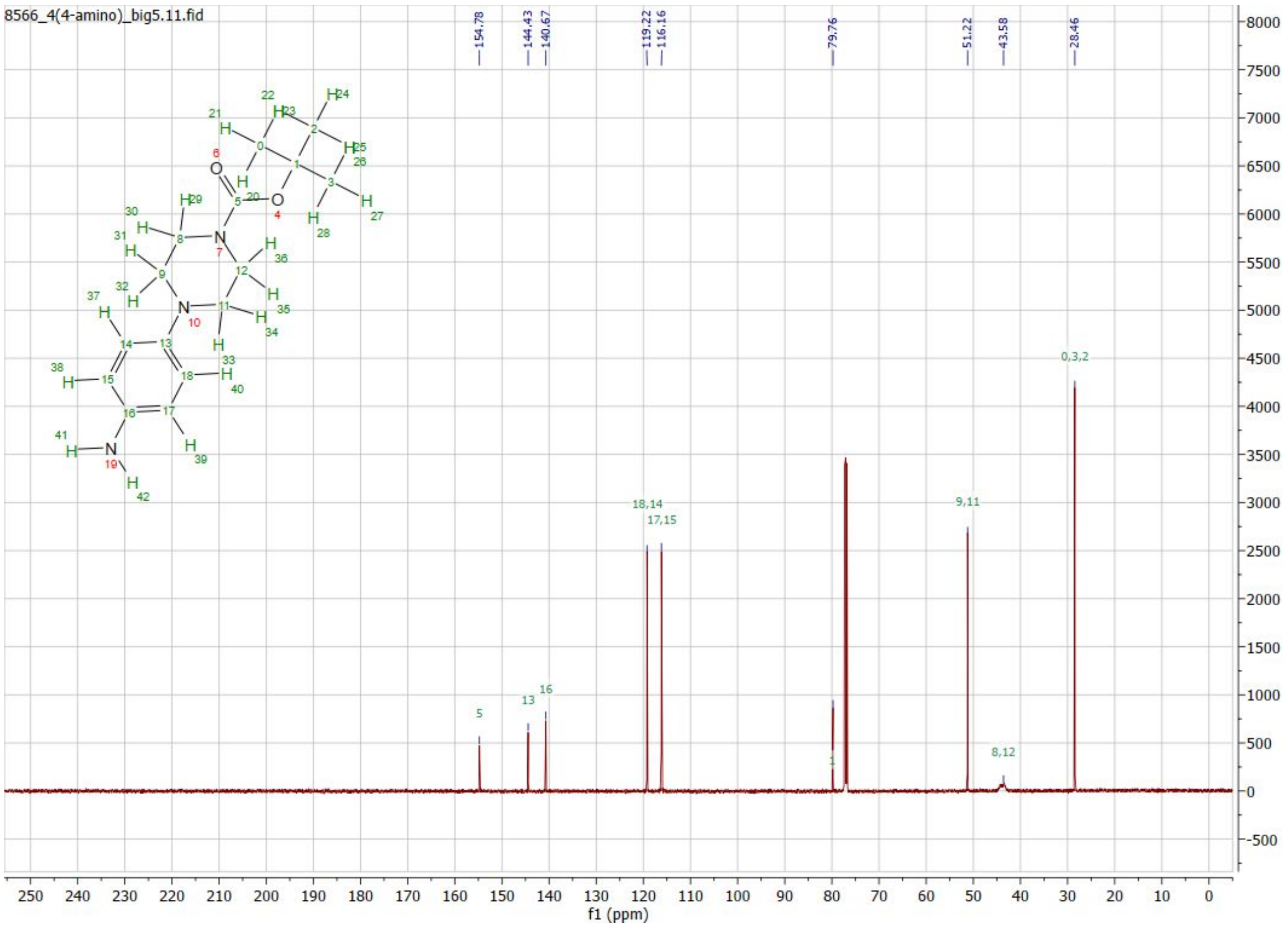

^{1}H-^{1}H COSY

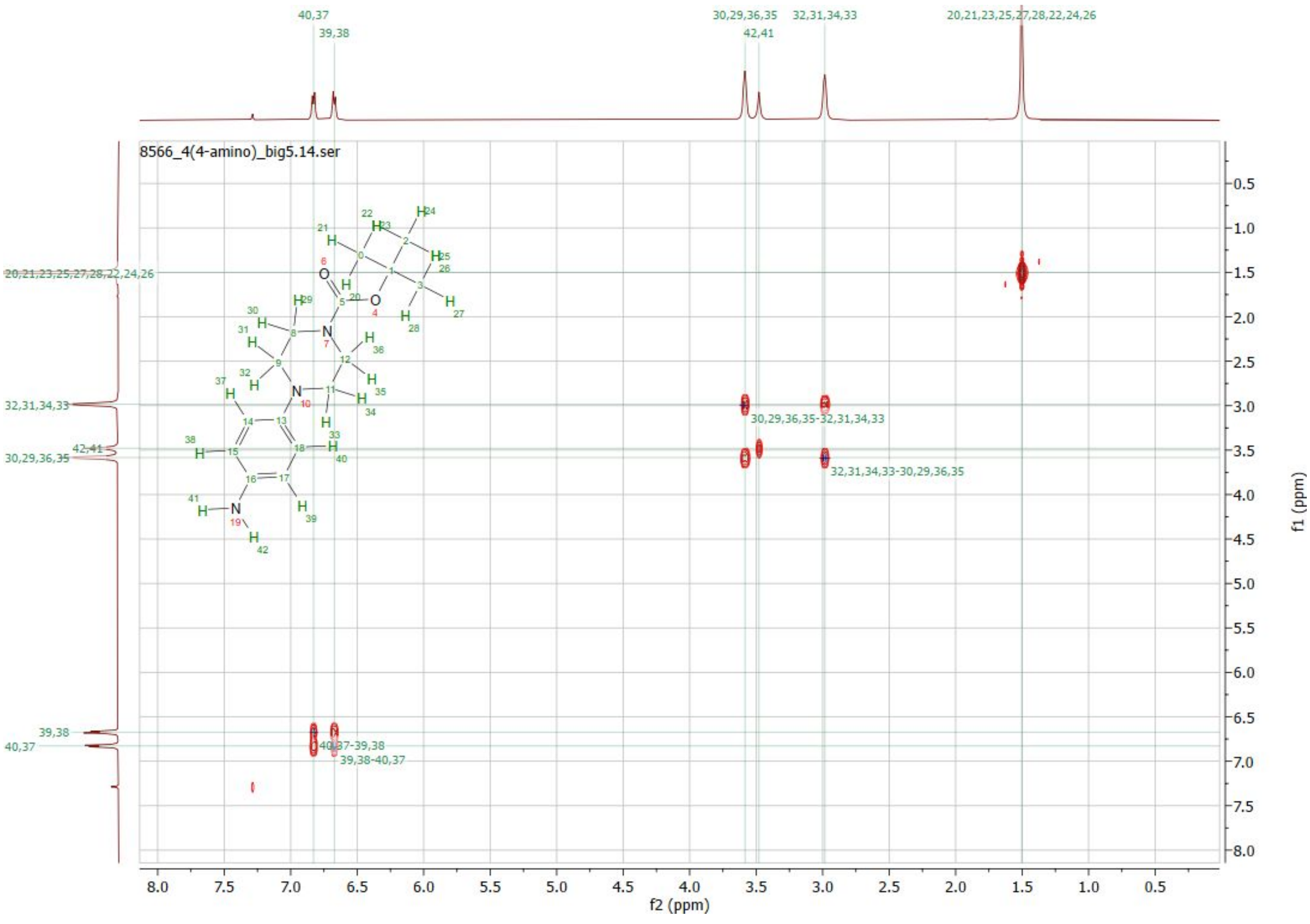

Pure shift ^{1}H-^{13}C HSQC

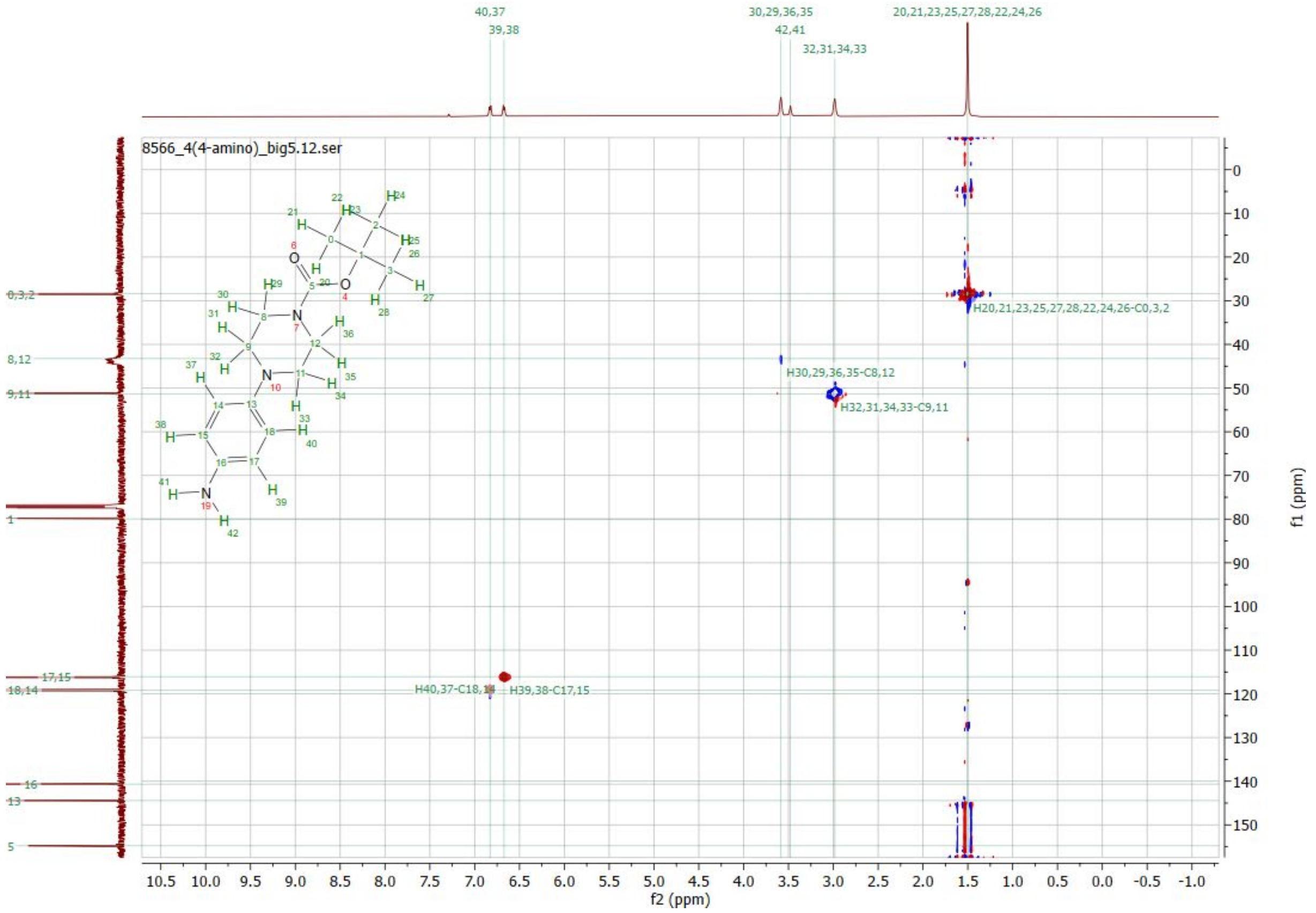

^{1}H-^{13}C HMBC

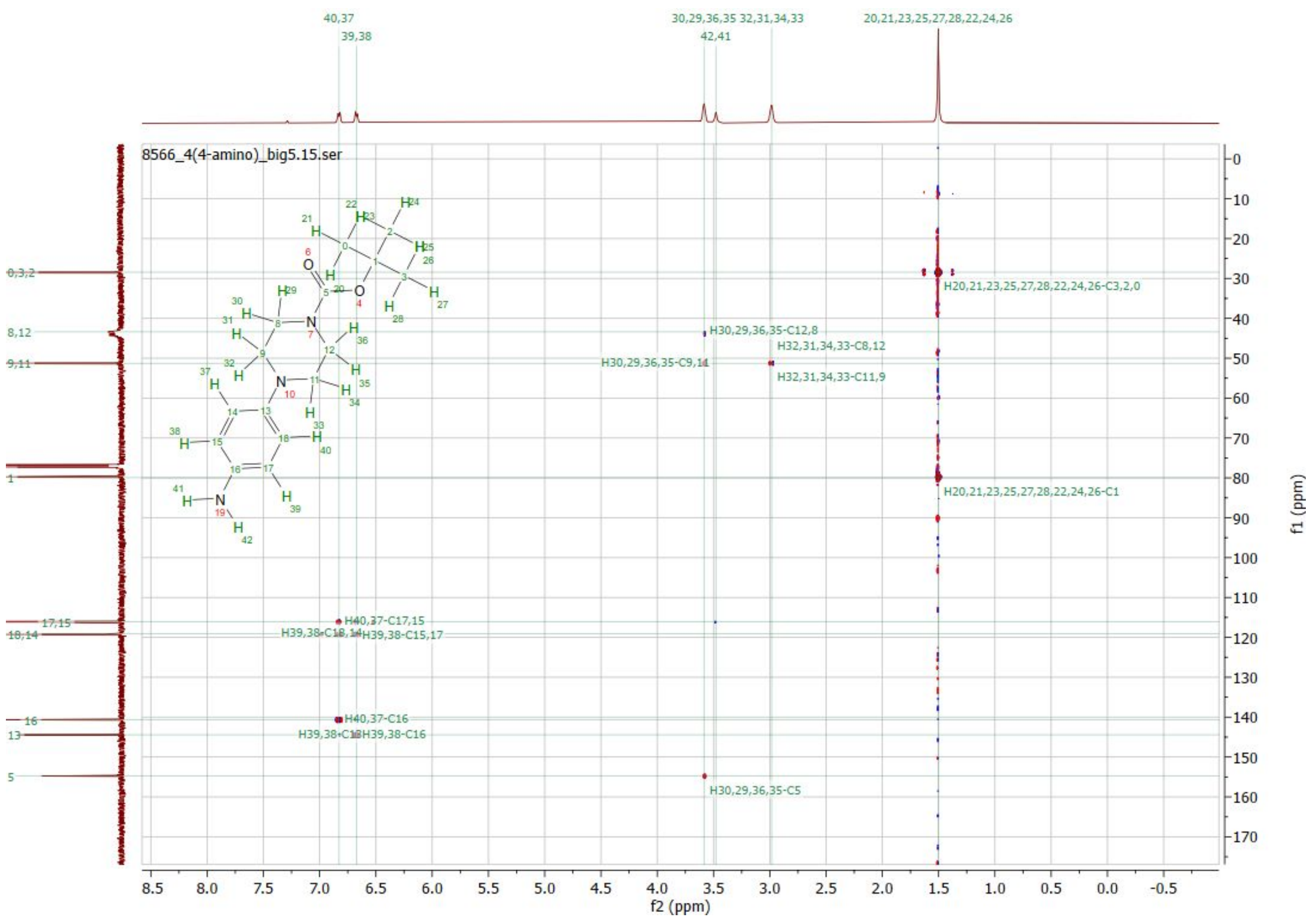

4.5 (R)-(+)-N-(1-phenylethyl)succinamic acid

^{1}H NMR

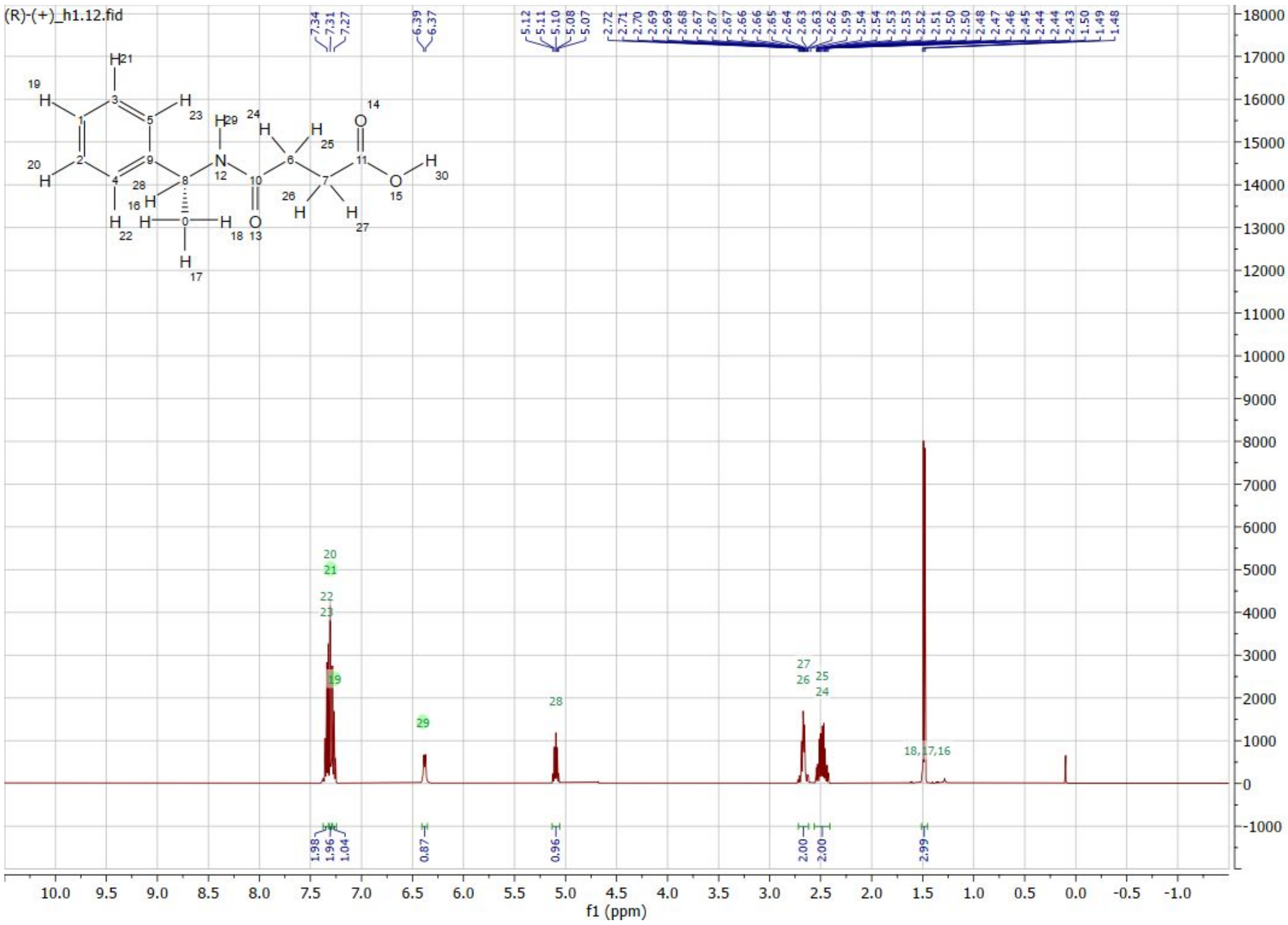

^{13}C NMR

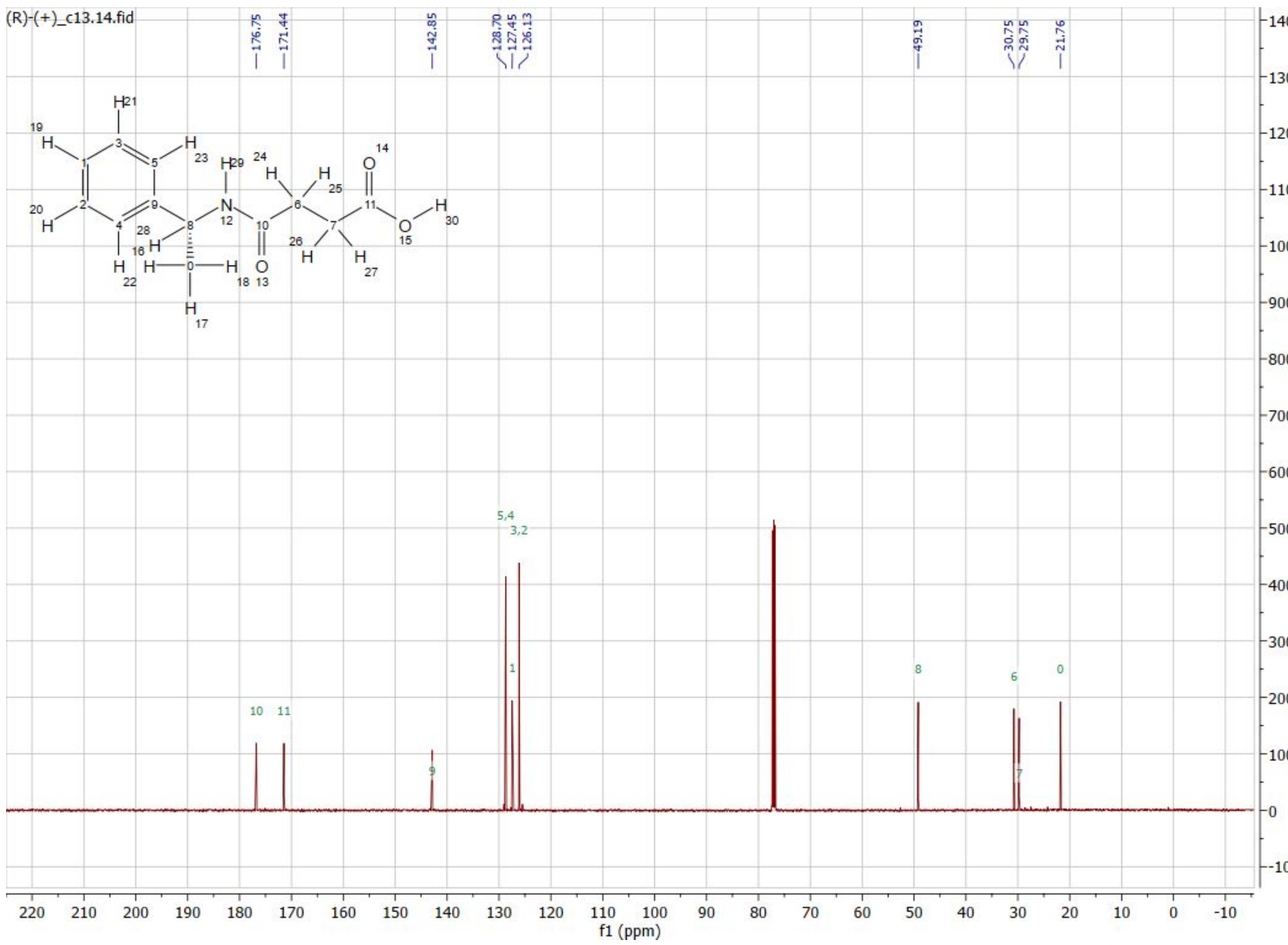

^{1}H-^{1}H COSY

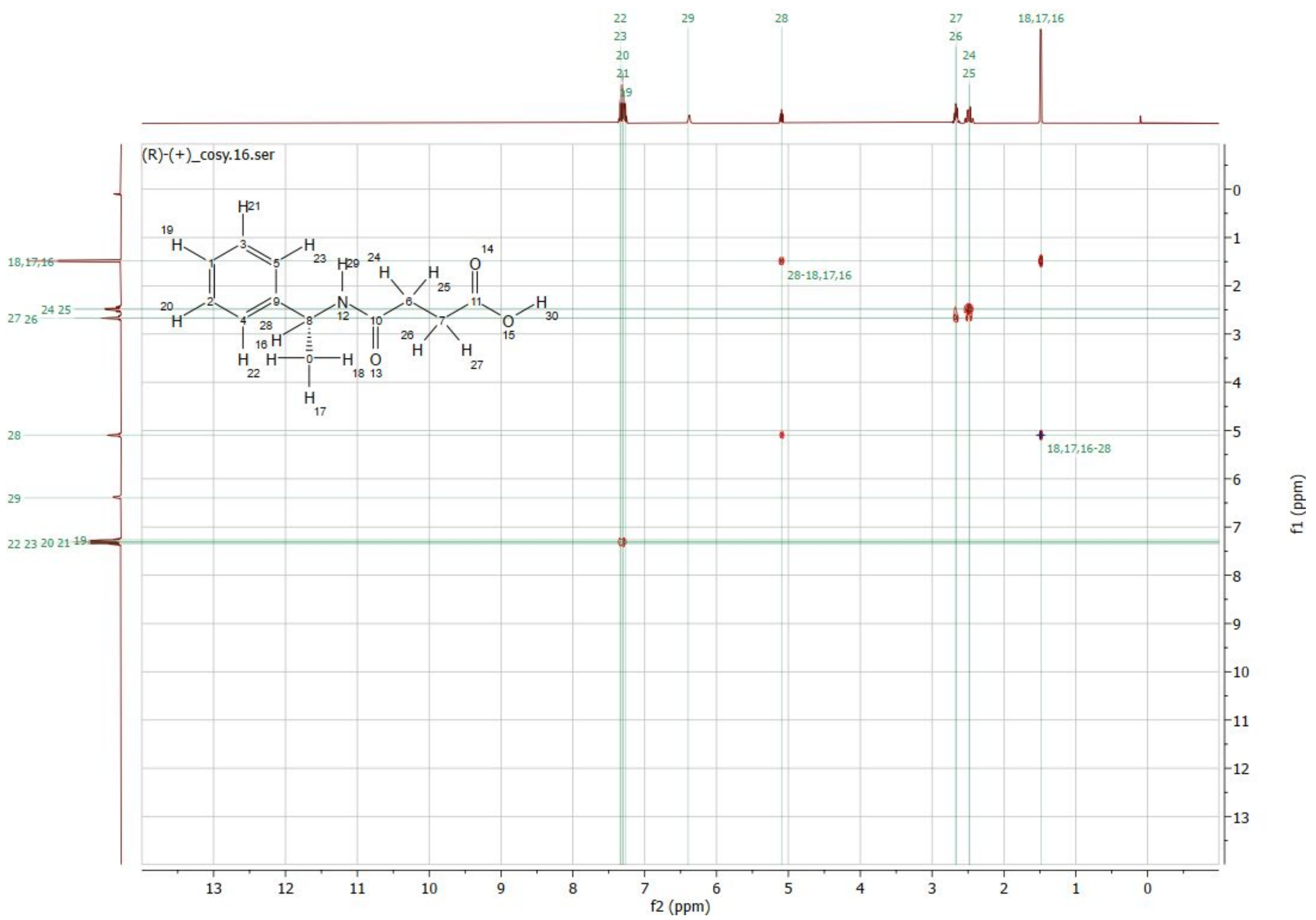

Pure shift ^{1}H-^{13}C HSQC

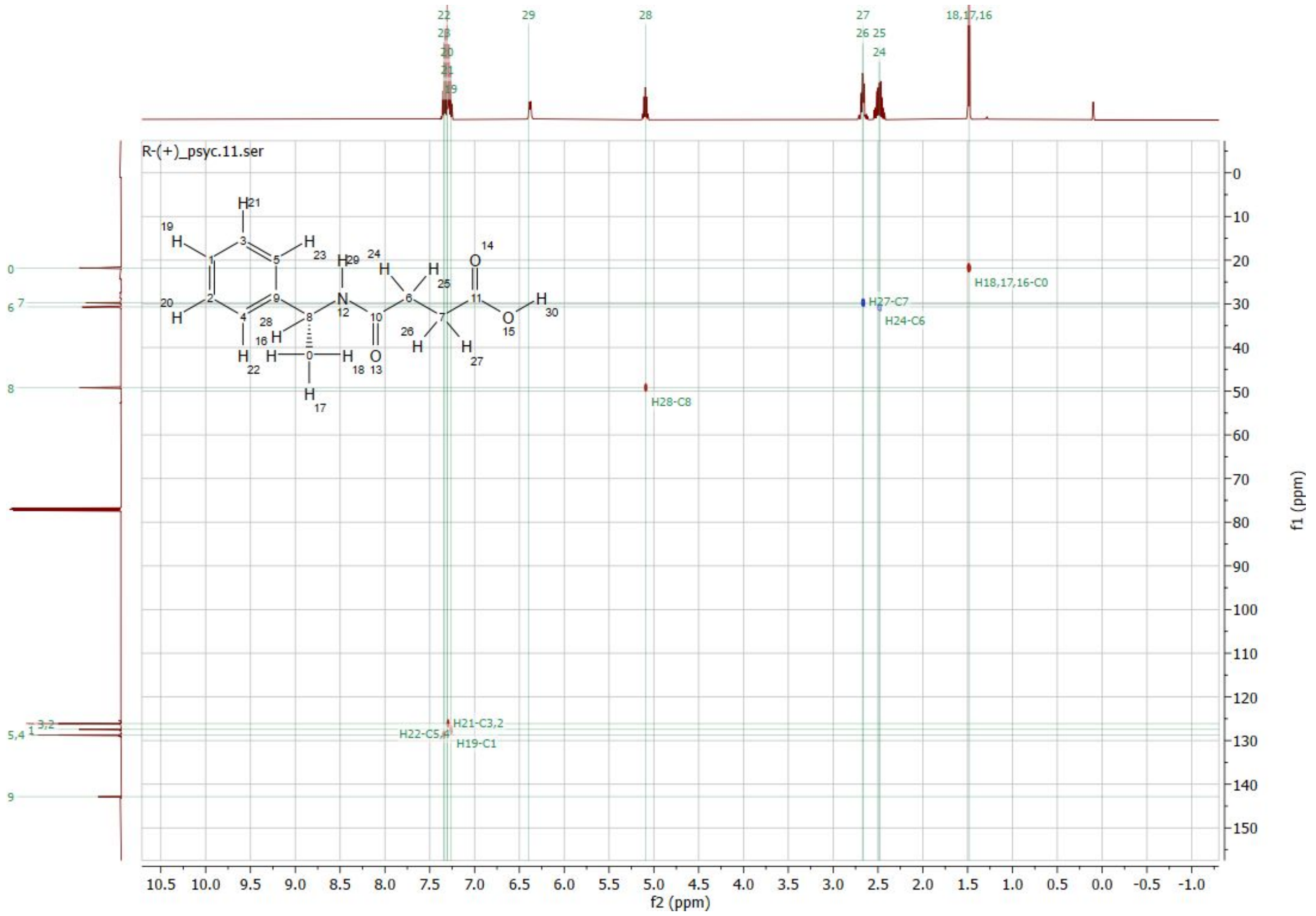

^{1}H-^{13}C HMBC

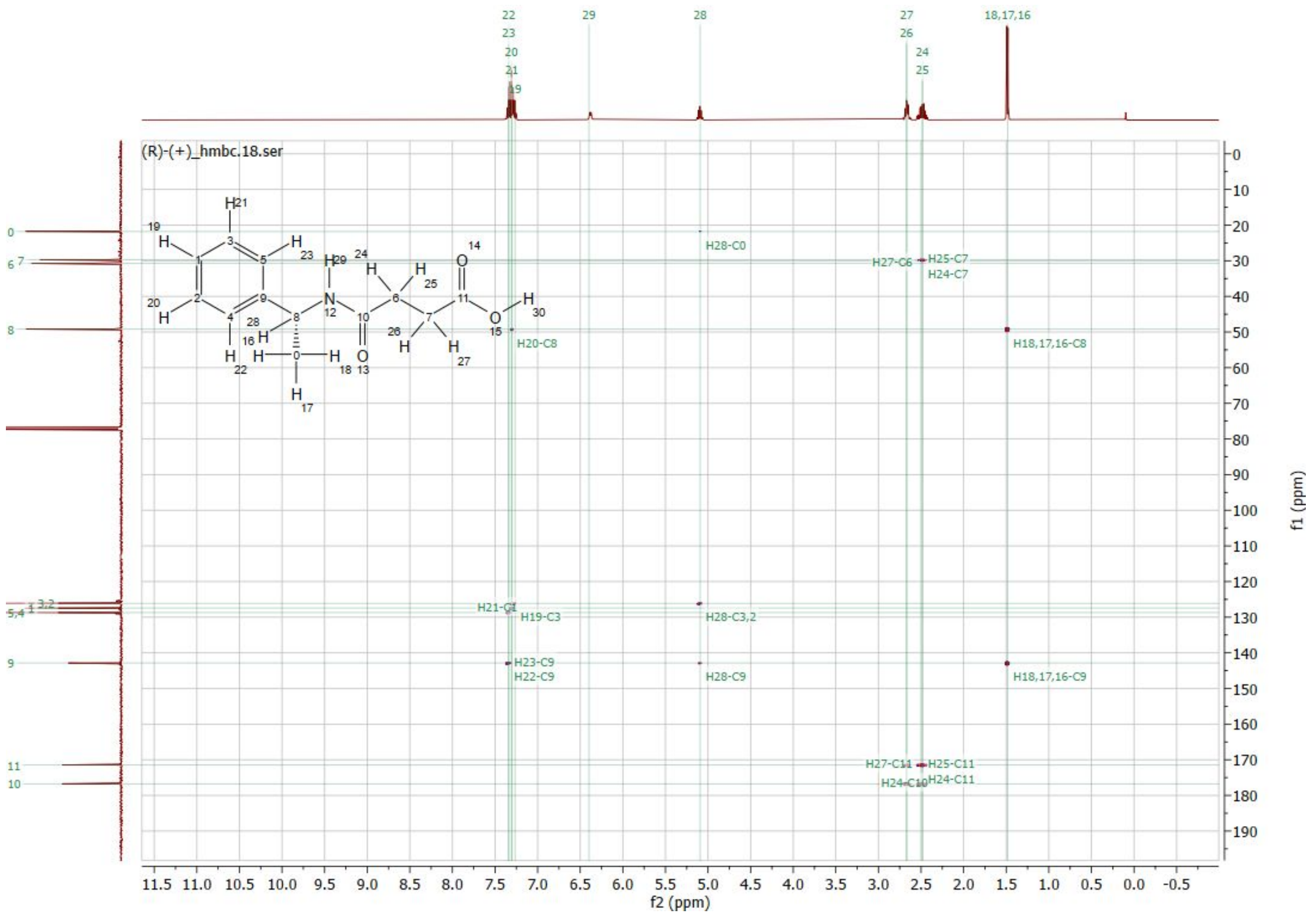

References

1. Yiu, C.; Honoré, B.; Gerrard, W.; Napolitano-Farina, J.; Russell, D.; Trist, I. M. L.; Dooley, R.; Butts, C. P. IMPRESSION Generation 2 – Accurate, Fast and Generalised Neural Network Model for Predicting NMR Parameters in Place of DFT. *Chem. Sci.* **2025**, *16*, 8377–8382.

2. Landrum, G.; et al. *RDKit: Open-Source Cheminformatics*, Ver. 2025.03.6. https://doi.org/10.5281/zenodo.16996017

3. Bratholm, L. A.; Gerrard, W.; Anderson, B.; Bai, S.; Choi, S.; Dang, L.; Hanchar, P.; Howard, A.; Kim, S.; Kolter, Z.; Kondor, R.; Kornbluth, M.; Lee, Y.; Lee, Y.; Mailoa, J. P.; Nguyen, T. T.; Popovic, M.; Rakocevic, G.; Reade, W.; Song, W.; Stojanovic, L.; Thiede, E. H.; Tijanic, N.; Torrubia, A.; Willmott, D.; Butts, C. P.; Glowacki, D. R. A Community-Powered Search of Machine Learning Strategy Space to Find NMR Property Prediction Models. *PLoS One* **2021**, *16*, e0253612.

4. He, K.; Zhang, X.; Ren, S.; Sun, J. Delving Deep into Rectifiers: Surpassing Human-Level Performance on ImageNet Classification. *arXiv* 2015. https://doi.org/10.48550/arXiv.1502.01852

5. Cho, K.; van Merrienboer, B.; Gulcehre, C.; Bahdanau, D.; Bougares, F.; Schwenk, H.; Bengio, Y. Learning Phrase Representations Using RNN Encoder-Decoder for Statistical Machine Translation. *arXiv* 2014. https://doi.org/10.48550/arXiv.1406.1078

6. Yun, S.; Jeong, M.; Kim, R.; Kang, J.; Kim, H. J. Graph Transformer Networks. *arXiv* 2019. https://doi.org/10.48550/arXiv.1911.06455

7. Vaswani, A.; Shazeer, N.; Parmar, N.; Uszkoreit, J.; Jones, L.; Gomez, A. N.; Kaiser, L.; Polosukhin, I. Attention Is All You Need. *arXiv* 2017. https://doi.org/10.48550/arXiv.1706.03762

8. You, Y.; Li, J.; Reddi, S.; Hseu, J.; Kumar, S.; Bhojanapalli, S.; Song, X.; Demmel, J.; Keutzer, K.; Hsieh, C.-J. Large Batch Optimization for Deep Learning: Training BERT in 76 Minutes. *arXiv* 2019. https://doi.org/10.48550/arXiv.1904.00962

9. Smith, L. N. Cyclical Learning Rates for Training Neural Networks. *arXiv* 2015. https://doi.org/10.48550/arXiv.1506.01186

10. Kim, S.; Chen, J.; Cheng, T.; Gindulyte, A.; He, J.; He, S.; Li, Q.; Shoemaker, B. A.; Thiessen, P. A.; Yu, B.; Zaslavsky, L.; Zhang, J.; Bolton, E. E. PubChem 2025 Update. *Nucleic Acids Res.* **2025**, *53*, D1516–D1525.

11. Zdrazil, B.; Felix, E.; Hunter, F.; Manners, E. J.; Blackshaw, J.; Corbett, S.; de Veij, M.; Ioannidis, H.; Mendez Lopez, D.; Mosquera, J. F.; Magarinos, M. P.; Bosc, N.; Arcila, R.; Kizilören, T.; Gaulton, A.; Bento, A. P.; Adasme, M. F.; Monecke, P.; Landrum, G. A.; Leach, A. R. The ChEMBL Database in 2023: A Drug Discovery Platform Spanning Multiple Bioactivity Data Types and Time Periods. *Nucleic Acids Res.* **2024**, *52*, D1180–D1192.

12. Halgren, T. A. Merck Molecular Force Field. I. Basis, Form, Scope, Parameterization, and Performance of MMFF94. *J. Comput. Chem.* **1996**, *17*, 490–519.

13. The pandas Development Team. *pandas* (version 2.3.2); Zenodo, 2025. https://doi.org/10.5281/zenodo.16918803

14. Fey, M.; Lenssen, J. E. Fast Graph Representation Learning with PyTorch Geometric. *arXiv* 2019. https://doi.org/10.48550/arXiv.1903.02428

15. Wildman, S. A.; Crippen, G. M. Prediction of Physicochemical Parameters by Atomic Contributions. *J. Chem. Inf. Comput. Sci.* **1999**, *39*, 868–873.

16. Krzyzanowski, A.; Pahl, A.; Grigalunas, M.; Waldmann, H. Spacial Score – A Comprehensive Topological Indicator for Small-Molecule Complexity. *J. Med. Chem.* **2023**, *66*, 12739–12750.
